# ITGBL1 inhibits obesity and osteoporosis by maintaining adipogenic and osteogenic differentiation balance via the NEDD4–PPARγ–RUNX2 axis

**DOI:** 10.1038/s41419-026-09220-z

**Published:** 2026-09-02

**Authors:** Hao Tan, Jia-Shuai Zhong, Lin-Sen Li, Rui Zhang, Wen-Xin Yan, Tian-Hao Zhou, Qing-Shan Wang, Yu-Mei Feng

**Affiliations:** 1 Department of Biochemistry and Molecular Biology, Tianjin Medical University Cancer Institute and Hospital, National Clinical Research Center of Cancer, Tianjin, 300060 China; 2 Key Laboratory of Cancer Immunology and Biotherapy of Tianjin, Tianjin Medical University Cancer Institute and Hospital, Tianjin, 300060 China

**Keywords:** Obesity, Mesenchymal stem cells, Osteoporosis

## Abstract

Obesity and osteoporosis are two major public health concerns. Dysregulation of mesenchymal stem cell (MSC) lineage commitment has been closely linked to an imbalance between adipogenic and osteogenic differentiation, which leads to abnormal lipid accumulation and impaired bone homoeostasis. However, as the underlying mechanisms remain unclear, effective treatment options for these two tightly linked diseases are still lacking. Herein, integrin subunit β-like 1 (ITGBL1) is identified as a key regulator of the adipogenic and osteogenic lineage commitment of MSCs. ITGBL1 is downregulated in MSCs from mouse models of high-fat diet (HFD)-induced obesity and ovariectomy (OVX)-induced osteoporosis. ITGBL1 deficiency promotes the adipogenic differentiation of bone marrow-derived MSCs, adipose-derived MSCs and adipogenic precursor cells while impairing the osteogenic differentiation of bone marrow-derived MSCs and osteogenic precursor cells. Mechanistically, ITGBL1 promotes the NEDD4-mediated ubiquitination and proteasomal degradation of PPARγ. ITGBL1 deficiency stabilizes PPARγ to activate adipogenic transcriptional programs. PPARγ further undergoes reciprocal transrepression with RUNX2 in specific cellular contexts, and these transcription factors transrepress and transactivate *ITGBL1* in osteogenic and adipogenic lineage precursor cells, respectively. Thus, ITGBL1, PPARγ and RUNX2 constitute an interconnected regulatory network‌ that governs the lineage commitment of MSCs. Therapeutically, adeno-associated virus serotype 9-mediated ITGBL1 supplementation significantly alleviates obesity and/or osteoporosis in HFD-fed mice, OVX mice and systemic ITGBL1-deficient mice. Our findings reveal that ITGBL1 is crucial for maintaining the balance between the adipogenic and osteogenic differentiation of MSCs and has notable medicinal effects for the prevention and treatment of obesity and osteoporosis.

**Figure:**
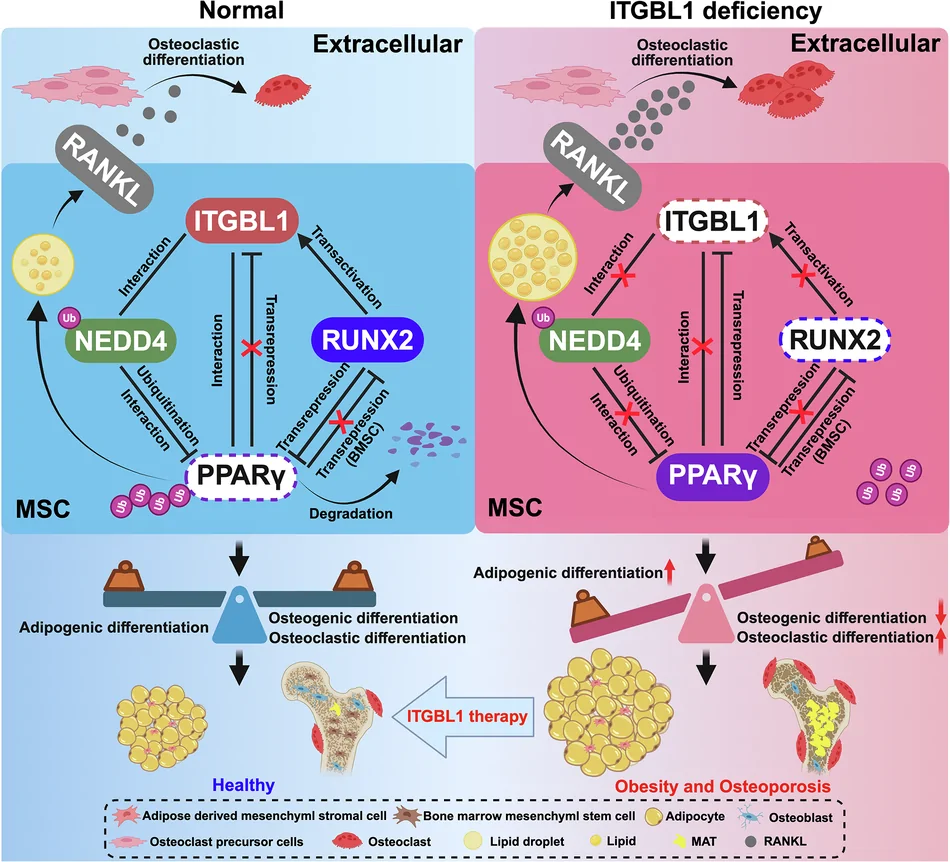

## Introduction

Obesity and osteoporosis are two major metabolic diseases that have become global public health concerns. Obesity is characterized by the excessive accumulation of body fat and is defined as a body mass index (BMI) ≥ 30 kg/m^2^ in adults and a BMI > 2 standard deviations (SD) above the median in school-aged children and adolescents [1]. Obesity substantially increases the risk of metabolic diseases, cardiovascular diseases, nervous system diseases, skeletal system diseases, respiratory system diseases, and various cancers, all of which lead to a reduced quality of life and a shortened life expectancy [2, 3]. In recent decades, the prevalence of obesity has increased, and the average age of onset has decreased in most countries [1]. Osteoporosis is defined by low bone mineral density (BMD) and is characterized by reduced bone mass and deterioration of bone microarchitecture, leading to increased bone fragility and a greater risk of fractures [4]. Globally, one in three women and one in five men older than 50 years have experienced osteoporotic fractures during their lifetime [4]. Therefore, effective prevention and management strategies for both obesity and osteoporosis are urgently needed.

An inverse relationship between bone mass and fat mass has long been noted [5–7]. Excessive fat mass is an independent risk factor for osteoporosis and osteoporotic fractures in adults [8, 9], children and adolescents [10], regardless of age or sex. In turn, bone marrow adipose tissue (BMAT) is increased markedly with aging and in patients with osteoporosis [11, 12]. Evidence from in vivo and in vitro studies has demonstrated that obesity and osteoporosis occur simultaneously in a high-fat diet (HFD)-induced mouse model [13] and that fat mass negatively affects bone mass [8]. The reciprocal negative regulation between adipogenesis and osteogenesis is driven primarily by the lineage selection bias of mesenchymal stem cells (MSCs), the common progenitors of both osteoblasts and adipocytes [14, 15]. Additionally, lipid oxidation products hinder osteogenic differentiation [16, 17].

Peroxisome proliferator-activated receptor gamma (PPARγ) and runt-related transcription factor 2 (RUNX2) are lineage-determining transcription factors that regulate the adipogenic and osteogenic differentiation of MSCs, respectively [18, 19]. PPARγ not only promotes the adipogenic differentiation of MSCs but also inhibits the osteogenic differentiation of MSCs [20]. Moreover, bone marrow adipogenic lineage precursor cells can promote osteoclast differentiation and bone resorption through interacting with monocyte‒macrophage lineage cells and producing large amounts of receptor activator of nuclear factor κB ligand (RANKL) [21, 22]. Therefore, in addition to the inherent reduction in bone formation due to impaired osteogenesis, increased BMAT mass further supports osteoclast differentiation [23–25]. Although the inverse relationship between bone mass and fat mass is known to arise from MSC lineage commitment toward either adipocytes or osteoblasts, the key regulator that coordinates the reciprocal suppression between PPARγ and RUNX2 to maintain the delicate balance between adipogenic and osteogenic differentiation remains unclear.

In this study, we identified integrin subunit beta-like 1 (ITGBL1) as a crucial regulator of the balance between the adipogenic and osteogenic differentiation of MSCs, with an underlying mechanism of biasing MSC lineage commitment towards osteogenic differentiation. We further elucidated the regulatory mechanisms among ITGBL1, PPARγ and RUNX2. Importantly, we found that ITGBL1 is downregulated in MSCs from mouse models of obesity induced by an HFD and mouse models of osteoporosis caused by ovariectomy (OVX). Adeno-associated virus serotype 9 (AAV9)-mediated ITGBL1 supplementation effectively alleviated obesity and osteoporosis in mice fed an HFD and in mice with systemic ITGBL1 deficiency and attenuated bone loss in OVX-induced osteoporotic mice. Therefore, ITGBL1 has notable medicinal effects for the prevention and treatment of obesity and osteoporosis.

## Materials and methods

### Animal experiments

To generate a diet-induced obesity (DIO) mouse model, 4-week-old male C57BL/6 J mice were fed an HFD (60% kcal from fat; Cat# D12492; Jiangsu Xietong Pharmaceutical Bio-engineering Co., Ltd.) for 12 or 16 weeks. Mice fed a chow diet (CD; 10% kcal from fat; Cat# XTC01FZ-001; Jiangsu Xietong Pharmaceutical Bio-engineering Co., Ltd., China) served as controls. To establish an osteoporosis mouse model, 12-week-old female C57BL/6 J mice underwent bilateral ovariectomy (OVX) to mimic postmenopausal osteoporosis, the mice underwent sham operation (sham) served as control, followed by CD feeding for 16 weeks. *Itgbl1* homozygous knockout (*Itgbl1*^–/–^) C57BL/6 J mice were generated by Cyagen Biosciences (China). Briefly, a ribonucleoprotein composed of two guide RNAs targeting regions upstream and downstream of *Itgbl1* exon 2 and Cas9 protein was injected into the cytoplasm of single-celled zygotes from C57BL/6 J mice via microinjection. Surviving zygotes were then transferred into the fallopian tubes of pseudopregnant female mice to generate F0 mice. Genomic DNA was extracted from tail tip tissue, and F0 mice with the desired genotype were identified by PCR of tail genomic DNA, with primers listed in Supplementary Table 1. F0 chimeric mice confirmed to harbour the targeted deletion mutation were then mated with wild-type (WT) C57BL/6 J mice to select F1 *Itgbl1*^+/–^ mice that stably inherited the knockout allele. Heterozygous pairs were subsequently interbred to generate F2 *Itgbl1*^–/–^ mice, which served as homozygous systemic knockout mice for the experiments. All the mice were housed under specific pathogen-free conditions.

For the treatment of obese and osteoporosis mice, AAV9-delivered *Itgbl1* (AAV9-Itgbl1) or vector control (AAV9-Vector; Beijing Tsingke Biotech Co., Ltd., China) was administered to 4-week-old (for obese) or 12-week-old mice (for osteoporosis) via tail vein injection (2 × 10^11^ vg) or intra-articular injection into the knee joint cavity (5 × 10^10^ vg) for 12 or 16 weeks. The AAV9 dose used in this study was selected on the basis of preliminary experiments and previously reported AAV9 studies showing efficient tissue transduction within a comparable dosing range [26–28]. Following sacrifice, eyeball blood, adipose tissue, lower limbs and other organs were harvested for serological or histological analyses. The protocol for animal experiments were approved and performed according to the Animal Ethics Committee of Tianjin Medical University Cancer Institute & Hospital (Approval Number: AE-2022065).

### Micro-computed tomography (micro-CT) analysis

After anaesthesia, the mice were scanned longitudinally with continuous rotation using an IRIS CT scanner (Inviscan SAS, Strasbourg, France). The scan parameters included tube voltage of 80 kV, tube current of 1 mA, 2000 full-scan projections, exposure time of 56 ms, voxel size of 60 μm, mouse field of view (FOV) of 40 mm, and axial FOV of 90 mm. The scanned images were analysed using Imalytics Preclinical 3.1 software (Gremse-IT GmbH, Aachen, Germany). For trabecular bone analysis, the region of interest (ROI) was defined as extending 3 mm proximally from the distal femoral growth plate, and the values of BMD, bone volume/tissue volume (BV/TV), trabecular number (Tb.N), bone surface/tissue volume (BS/TV), trabecular separation (Tb.Sp), and trabecular thickness (Tb.Th) were collected and calculated. For cortical bone analysis, the ROI was defined as the area between 2 mm and 3 mm proximal to the distal femoral growth plate, and the values of cortical BMD (Cor-BMD) and cortical bone thickness (Cb.Th) were collected and calculated.

For three-dimensional reconstruction of the trabecular and cortical bone, femurs were dissected and fixed in 10% neutral buffered formalin for 24–48 h and then stored in 70% ethanol. The femur was placed in a sample tube, with its long axis fixed perpendicular to the X-ray source to minimize artefacts caused by shaking during scanning. Scanning was performed using a SCANCO Medical micro-CT100 scanner (Scanco Medical AG, Basserdorf, Switzerland) at 55 kVp and 200 μA. Volumetric reconstructions of global and local trabecular and cortical bone of the femur were generated using the built-in software of the scanner. The reconstructed images were then adjusted and exported using micro-CT Ray V4.2 software (Scanco Medical AG, Basserdorf, Switzerland). The ROI for local trabecular and cortical bone reconstruction was defined as the area between 2 mm and 3 mm proximal to the distal femoral growth plate.

### Enzyme-linked immunosorbent assay (ELISA)

Mouse eyeball blood was collected, and serum was obtained by centrifuging the blood at 3,000 rpm for 10 min at 4 °C. The serum levels of RANKL, osteoprotegerin (OPG), and the N-terminal propeptide of type I procollagen (PINP) were quantified using ELISA kits (ABclonal Biotechnology, Wuhan, China; Cat# RK00149, RK04789 and RK09130) according to the manufacturer’s instructions.

### Histology analysis

Tissues dissected from mice were rinsed with pre-chilled phosphate-buffered saline (PBS) and fixed in 10% formalin. Bone specimens were decalcified in a decalcification solution containing 10% EDTA (pH 7.4; Sangon Biotech, Shanghai, China; Cat# E671001-0500) for 2 weeks. Formalin-fixed and paraffin-embedded (FFPE) tissue sections were prepared. Haematoxylin and eosin (H&E) and immunohistochemistry (IHC) staining were performed as described previously [29]. All antibodies and their dilutions are listed in Supplementary Table 2. To evaluate osteoclast differentiation in mouse bone tissues, tartrate-resistant acid phosphatase (TRAP) staining of FFPE tissue sections was performed using a TRAP Stain Kit (Solarbio, Beijing, China; Cat# G1492) according to the manufacturer’s instructions. The number of TRAP-positive osteoclasts on the bone surface (BS) was counted from three random fields at 200× magnification.

### Isolation and culture of MSCs and bone marrow macrophages (BMMs)

Primary adipose-derived MSCs (ADMSCs) were isolated from the white adipose tissue (WAT) of mice using 10 g/L type I collagenase (Sigma‒Aldrich, Germany; Cat# C5138). Bone marrow cells were isolated by flushing the femoral bone marrow cavity, and erythrocytes were removed using erythrocyte lysis buffer (Servicebio, Wuhan, China; Cat# G2015). Primary bone marrow MSCs (BMSCs) were isolated by negative selection with CD11b magnetic beads (Miltenyi Biotec, Germany; Cat# 130-049-601), while primary BMMs were isolated by positive selection using the same beads. MSCs were cultured in Dulbecco’s Modified Eagle Medium (DMEM; Thermo Fisher Scientific/Gibco, Gaithersburg, MD, USA) supplemented with 10% foetal bovine serum (FBS; Thermo Fisher Scientific/Gibco) and 1% penicillin-streptomycin solution (Thermo Fisher Scientific/Gibco). BMMs were cultured in minimum essential medium-alpha (α-MEM; Thermo Fisher Scientific/Gibco) supplemented with 50 ng/mL RANKL (Peprotech, USA; Cat# 315-11), 50 ng/mL macrophage colony-stimulating factor (M-CSF; Peprotech; Cat# 315-02), 10% FBS and 1% penicillin-streptomycin solution. The cells were maintained at 37 °C in a humidified incubator with 5% CO_2_. MSCs at passages 2–5 were used for subsequent experiments.

### Flow cytometry analysis

After trypsin digestion, centrifugation, and resuspension, the cultured MSCs were equally divided into tubes, and fluorescein-labelled antibodies were added, followed by incubation in the dark at 37 °C for 10 min. Pre-chilled PBS was then added to each tube, and the samples were loaded into a CytoFLEX LX Flow Cytometer and analysed using CytExpert Software (Beckman Coulter, USA; RRID: SCR_017217). Detailed information about the antibodies is listed in Supplementary Table 2.

### Induction and identification of the adipogenic and osteogenic differentiation of MSCs and precursor cells

To induce the adipogenic differentiation of primary MSCs and the adipogenic precursor cell line 3T3-L1 (RRID: CVCL_0123), cells were cultured in adipogenic induction medium supplemented with 1 μM dexamethasone (Sigma‒Aldrich, Cat# D4902), 10 μM insulin (Selleck Chemicals, Cat# S6955), 0.5 mM 3-isobutyl-1-methylxanthine (MedChemExpress, Cat# HY-12318), 1 μM indomethacin (MedChemExpress, Cat# HY-14397), 10% FBS, and 1% penicillin-streptomycin solution. After 2–3 weeks of adipogenic induction, Oil Red O staining was performed using an Oil Red O Stain Kit (Solarbio, Cat# G1261) according to the manufacturer’s instructions, and the number of Oil Red O-positive cells was quantified using ImageJ software (NIH, Bethesda, USA; RRID: SCR_003070). For Nile red staining, cells were fixed with 4% paraformaldehyde and then stained with Nile red solution (MedChemExpress, Cat# HY-D0718) at 37 °C for 15 min. After 4′,6-diamidino-2-phenylindole (DAPI; Sigma‒Aldrich, Cat# D9542) staining, images were acquired by using fluorescence microscopy, and the stained area was analysed with ImageJ software.

To induce the osteogenic differentiation of primary BMSCs and the osteogenic precursor cell line MC3T3-E1 (RRID: CVCL_0409), cells were cultured in osteogenic induction medium supplemented with 50 μM ascorbic acid (Sigma‒Aldrich, Cat# A4403), 10 mM β-glycerophosphate (Sigma‒Aldrich, Cat# G9422), 10 nM dexamethasone (MedChemExpress, Cat# HY-14648), 10% FBS, and 1% penicillin-streptomycin solution. After 7 days of osteogenic induction, the cells were stained with an Alkaline Phosphatase (ALP) Stain Kit (Solarbio, Cat# G1481) according to the manufacturer’s instructions, and the stained area was quantified using ImageJ software. For Alizarin Red S (ARS) staining, after 2–3 weeks of induction, the cells were stained with an ARS Staining Quantitative Detection Kit (Solarbio, Cat# G3283) according to the manufacturer’s instructions, and the stained cultures were then destained and quantified. The calcium salt content was determined by ARS concentration on the basis of a cetylpyridinium chloride standard curve. All the cell lines were authenticated by short tandem repeat profiling and routinely tested for mycoplasma contamination.

### Lentiviral infection

3T3-L1 and MC3T3-E1 cells were infected with recombinant lentiviruses carrying enhanced green fluorescent protein (GFP)-tagged short hairpin RNA (shRNA) targeting mouse *Itgbl1* (3T3-L1-shItgbl1-GFP and MC3T3-E1-shItgbl1-GFP) or the corresponding negative controls (3T3-L1-shControl-GFP and MC3T3-E1-shControl-GFP), and the targeted sequences are listed in Supplementary Table 3. ADMSCs, BMSCs, 3T3-L1 cells and MC3T3-E1 cells were also infected with recombinant lentiviruses carrying GFP and Flag-tagged full-length (FL) mouse *Itgbl1* cDNA (CCDS27354.1; ADMSC-Itgbl1-Flag-GFP, BMSC-Itgbl1-Flag-GFP, 3T3-L1-Itgbl1-Flag-GFP, and MC3T3-E1-Itgbl1-Flag-GFP) or vector controls (ADMSC-Vector-Flag-GFP, BMSC-Vector-Flag-GFP, 3T3-L1-Vector-Flag-GFP, and MC3T3-E1-Vector-Flag-GFP). Cells were selected with 2 μg/mL puromycin for 2 weeks to stably silence endogenous ITGBL1 or express exogenous ITGBL1, and successful modification was confirmed by reverse transcription‒quantitative PCR (RT‒qPCR). All lentiviruses were constructed by GeneChem Co., Ltd. (Shanghai, China).

### Plasmids and small interfering RNA (siRNA) transfection

The siRNAs targeting *Itgbl1* and *Nedd4* were designed and synthesized by Tsingke Biotech Co., Ltd. The siRNA sequences used to effectively knock down ITGBL1 and NEDD4 are listed in Supplementary Table 3, with siControl used as a negative control. FL human *ITGBL1* and *PPARG* cDNAs (CCDS9499.1, CCDS2609.1) and FL mouse *Pparg* and *Runx2* cDNAs (CCDS20439.1, CCDS37624.2) were cloned and inserted into pCDH-Flag, pCDH-HA or pCDH-His plasmids. The transfection of siRNAs and plasmids was performed using Lipofectamine 2000 (Thermo Fisher Scientific) according to the manufacturer’s instructions.

### RNA extraction and RT‒qPCR

Total RNA was extracted from cells or adipose tissue using TRIzol reagent (Thermo Fisher Scientific, Cat# 15596018CN). A total of 1 × 10^6^ cells or 200 mg of adipose tissue, disrupted by freezing in liquid nitrogen, was suspended in 1 mL of TRIzol and lysed at room temperature (RT) for 5 min. After 0.2 mL of chloroform was added, the solution was thoroughly mixed, incubated at RT for 5 min, and then centrifuged at 12,000 × g for 15 min at 4 °C. The supernatant was collected and mixed with 0.5 mL of isopropanol at RT for 5 min to precipitate the RNA. RNA pellets were harvested by centrifugation at 7000 × g for 15 min at 4 °C and then washed twice with 75% ethanol. Finally, the RNA pellet was dissolved in 50 μL of nuclease-free water at 60 °C for 10 min. Reverse transcription of the total RNA was performed using the SuperScript™ First-Strand Synthesis System (Thermo Fisher Scientific). qPCR was carried out using Taq Pro Universal SYBR qPCR Master Mix (Vazyme Medical Technology, Nanjing, China; Cat# Q712-03) with the primers listed in Supplementary Table 1. The expression level of the target gene was calculated by normalizing the average cycle threshold (Ct) values of the target gene to those of the housekeeping gene glyceraldehyde-3-phosphate dehydrogenase (*GAPDH*; ΔCt) and determined using the 2^−ΔCt^ method.

### RNA sequencing (RNA-seq) and data analysis

For RNA-seq, ADMSCs or BMSCs from WT or *Itgbl1*^–/–^ mice were seeded on 10 cm Petri dishes at a density of 1 × 10^6^ cells per dish and collected after 3 days of culture. Three biological replicates were obtained from different mice in each group. Total RNA was extracted from MSCs using TRIzol reagent (Invitrogen) and assessed with an Agilent 2100 BioAnalyzer (Agilent Technologies, Santa Clara, CA, USA) and a Qubit fluorometer (Thermo Fisher Scientific). The mRNA library was constructed using the Optimal Dual-mode mRNA Library Prep Kit (CapitalBio Technology) according to the manufacturer’s instructions. Briefly, the NEBNext Poly (A) mRNA Magnetic Isolation Module Kit (NEB, Cat# E7490) was used to enrich poly(A)-tailed mRNA molecules from 1 μg of total RNA. The mRNA was then fragmented into approximately 200 base pair pieces. First-strand cDNA was synthesized from the mRNA fragments using reverse transcriptase and random hexamer primers, followed by second-strand cDNA synthesis using DNA polymerase I and RNase H. The cDNA fragment ends were subjected to end repair, including the addition of a single “A” base, followed by adapter ligation. The cDNA fragments with adapters were amplified by PCR, and the PCR products were subsequently purified using Agencourt AMPure XP Beads (Beckman Coulter) and dissolved in EB buffer. The purified PCR products were quantified using the KAPA Library Quantification Kit (KAPA Biosystems, South Africa, Cat# KK4824) and analysed on an Agilent 2100 Bioanalyzer. Paired-end sequencing was performed to generate 150-bp paired-end reads on an Illumina NovaSeq sequencer (Illumina). Sequencing quality was assessed using FastQC (v0.11.5, https://www.bioinfo rmatics.babraham.ac.uk/projects/fastqc/), and low-quality reads were filtered out using NGSQC (v2.3.3). Clean reads were aligned to the reference genome using HISAT2 (v2.1.0) with default parameters. Processed reads from each sample were aligned against the mouse genome assembly GRCm38 using HISAT2. Gene expression analyses were performed with StringTie (v1.3.3b). Differentially expressed genes (DEGs) were identified using thresholds of |log_2_Fold change (Fc)| ≥1 and *P* < 0.05. Gene Ontology (GO) and Kyoto Encyclopedia of Genes and Genomes (KEGG) analyses were performed to identify significantly enriched biological processes and pathways among the DEGs. Additionally, gene set enrichment analysis (GSEA) was conducted using GSEA 4.3.3 software (Broad Institute, Cambridge, USA; RRID: SCR_003199) to investigate changes in pathways of interest.

### Single-cell sequencing data analysis

For human adipose tissue analysis, integrated single-nucleus RNA sequencing (snRNA–seq) datasets comprising 145,452 cells from 87 human adipose tissue samples were obtained from the Single Cell Portal (SCP1376) [30]. *ITGBL1* expression patterns were visualized across the annotated cell populations identified in the original dataset. For human skeletal tissue analysis, processed snRNA–seq datasets comprising 12,095 nuclei from intramembranous bone-forming niches and 12,219 nuclei from endochondral bone-forming niches derived from 12 human embryonic donors spanning 5–11 post-conception weeks were downloaded from the human embryonic skeletal development atlas (https://developmental.cellatlas.io/skeleton-development) [31]. *ITGBL1* expression was visualized across cell populations associated with intramembranous or endochondral osteogenesis according to the original cell annotations provided by the authors.

### Chromatin immunoprecipitation‒qPCR (ChIP‒qPCR)

In 3T3-L1 or MC3T3-E1 cells transfected with the *Pparg*-expressing plasmid, the *Runx2* proximal promoter region (**‒**252 to **‒**95 bp) and *Itgbl1* proximal promoter region (**‒**613 to **‒**503 bp), which contain putative PPARγ-binding elements, were enriched using an anti-PPARγ antibody. The *Pparg* proximal promoter region (**‒**493 to **‒**331 bp) and *Itgbl1* proximal promoter region (**‒**1665 to **‒**1492 bp), which contain putative RUNX2-binding elements, were enriched using an anti-RUNX2 antibody in 3T3-L1 or MC3T3-E1 cells transfected with a *Runx2*-expressing plasmid. ChIP assays were performed using the SimpleChIP® Enzymatic Chromatin IP Kit (magnetic beads; Cell Signalling Technology, USA; Cat# 9003S). Detailed information on the antibodies used in these assays is listed in Supplementary Table 2. The enriched DNA fragments were amplified by qPCR using the primers listed in Supplementary Table 1. The PCR products were visualized by electrophoresis on a 2% agarose gel.

### Dual luciferase reporter assay

The PGL3-Basic plasmids containing five copies of PPARγ response elements or five copies of RUNX2 response elements were constructed (Tsingke Biotech Co., Ltd.). The proximal promoter regions of *Runx2*, *Itgbl1* or *Pparg*, with or without PPARγ or RUNX2 binding elements, were synthesized and inserted into the respective PPARγ or RUNX2 luciferase reporter plasmids. The constructs were then cotransfected with PPARγ*-*expressing, RUNX2*-*expressing, or vector control plasmids, along with pRL-TK plasmids, into 3T3-L1 and MC3T3-E1 cells. After 48 h of incubation, the cells were lysed, and luciferase activity was measured using the Dual-Luciferase Reporter System (Promega, Madison, WI, USA) and quantified by normalization to the Renilla luciferase signal.

### Immunoblot (IB)

Cells were lysed using protein lysis buffer (20 mM Tris-HCl, 5 mM EDTA, 1% Triton X-100, 150 mM NaCl, and 1% DTT; pH 7.4) supplemented with 1% protease inhibitor cocktail (TransGen Biotech, Beijing, China; Cat# DL101-02). The lysates were separated by sodium dodecyl sulphate‒polyacrylamide gel electrophoresis (SDS‒PAGE) and transferred onto a nitrocellulose membrane (Millipore, Billerica, MA, USA; Cat# A30760431). The membrane was blocked with 5% skim milk in Tris-buffered saline with Tween 20 (TBST; 10 mM Tris, 150 mM NaCl, 0.05% Tween 20; pH 8.3) for 2 h at RT and then incubated overnight at 4 °C with a primary antibody diluted in 5% skim milk in TBST. The following day, the membrane was washed with TBST and incubated with horseradish peroxidase (HRP)-conjugated secondary antibody for 2 h at RT. After washing again with TBST, the immunoreactive protein bands were visualized using the SuperFemto ECL Chemiluminescence Kit (Vazyme Medical Technology, Cat# E423). Detailed information of the primary and secondary antibodies is listed in Supplementary Table 2. Uncropped blot images are presented in the supplementary material.

### Co-Immunoprecipitation (Co-IP) and ubiquitination assays

Cells were cultured on 10-cm dishes to the logarithmic growth phase. The cells were washed with pre-chilled PBS and lysed in IP lysis buffer (50 mM Tris-HCl, 1.5 mM EDTA, 100 mM NaCl, 10 mM NaF, 10% glycerol, and 1% NP-40; pH 8.0) supplemented with 1% protease inhibitor cocktail (TransGen Biotech, Cat# DL101-02). After centrifugation, the total protein content in the lysates was quantified, and 500 μg of protein was incubated with a primary antibody or an isotype-matched IgG control antibody (Proteintech, Cat# 30000-0-AP) overnight at 4 °C. The protein A/G magnetic beads (MedChemExpress, Cat# HY-K0202A) were pre-washed and incubated with the antigen-antibody complexes for 2 h at 4 °C. Alternatively, 500 μg of protein in the lysate was incubated with anti-Flag magnetic beads (MedChemExpress, Cat# HY-K0207) overnight at 4 °C. The antigen‒antibody‒bead complexes were washed five times with IP buffer, resuspended in SDS‒PAGE loading buffer, and boiled at 95 °C for 5 min. The samples were subsequently subjected to IB. Detailed information on the primary and secondary antibodies is provided in Supplementary Table 2. For detection of protein ubiquitination, cells were treated with 10 μM MG132 for 6 h prior to IP.

### GST pull-down (GST-PD)

PPARγ fused to glutathione S-transferase (PPARγ-GST) was purified from *E*. *coli* BL21 and bound to GSTSep Glutathione MagBeads (Yeasen, Shanghai, China; Cat# 20562ES08), followed by incubation with lysates containing the purified ITGBL1-His protein overnight at 4 °C. After the beads were washed three times with GST binding buffer (140 mM NaCl, 2.7 mM KCl, 10 mM Na_2_HPO_4_, and 1.8 mM KH_2_PO4; pH 7.4), the proteins bound to the beads were boiled in SDS‒PAGE loading buffer at 95 °C for 5 min and analysed by IB.

### Liquid chromatography‒tandem mass spectrometry (LC‒MS/MS)

Cells expressing Flag-tagged protein were harvested and lysed with IP lysis buffer supplemented with 1% protease inhibitor cocktail. After centrifugation, the protein lysate was incubated with a primary antibody or an isotype-matched IgG control antibody overnight at 4 °C. The protein A/G magnetic beads were pre-washed and then incubated with antigen-antibody complexes for 2 h at 4 °C. The antigen‒antibody‒bead complexes were washed five times with IP buffer, resuspended in SDS‒PAGE loading buffer, and boiled at 95 °C for 5 min. The immune complexes enriched with anti-Flag antibody or IgG antibody were subjected to SDS‒PAGE and stained with Coomassie Brilliant Blue. Protein bands were excised and analysed by LC‒MS/MS (GeneChem Co., Ltd), followed by IB for verification. Detailed information on the primary and secondary antibodies is provided in Supplementary Table 2.

### Statistical analysis

All experiments were performed at least two independent times in triplicate, and the data are presented as the mean ± SD. Details of the data presentation and statistical methods are described in the figure legends. Differences with *P* < 0.05 were considered statistically significant. All analyses were performed using GraphPad Prism 9.0 Software (GraphPad, La Jolla, CA, USA; RRID: SCR_002798).

## Results

### ITGBL1 is associated with adipogenic and osteogenic differentiation and is downregulated in obesity- and osteoporosis-related lineage imbalance

**Fig. 1:**
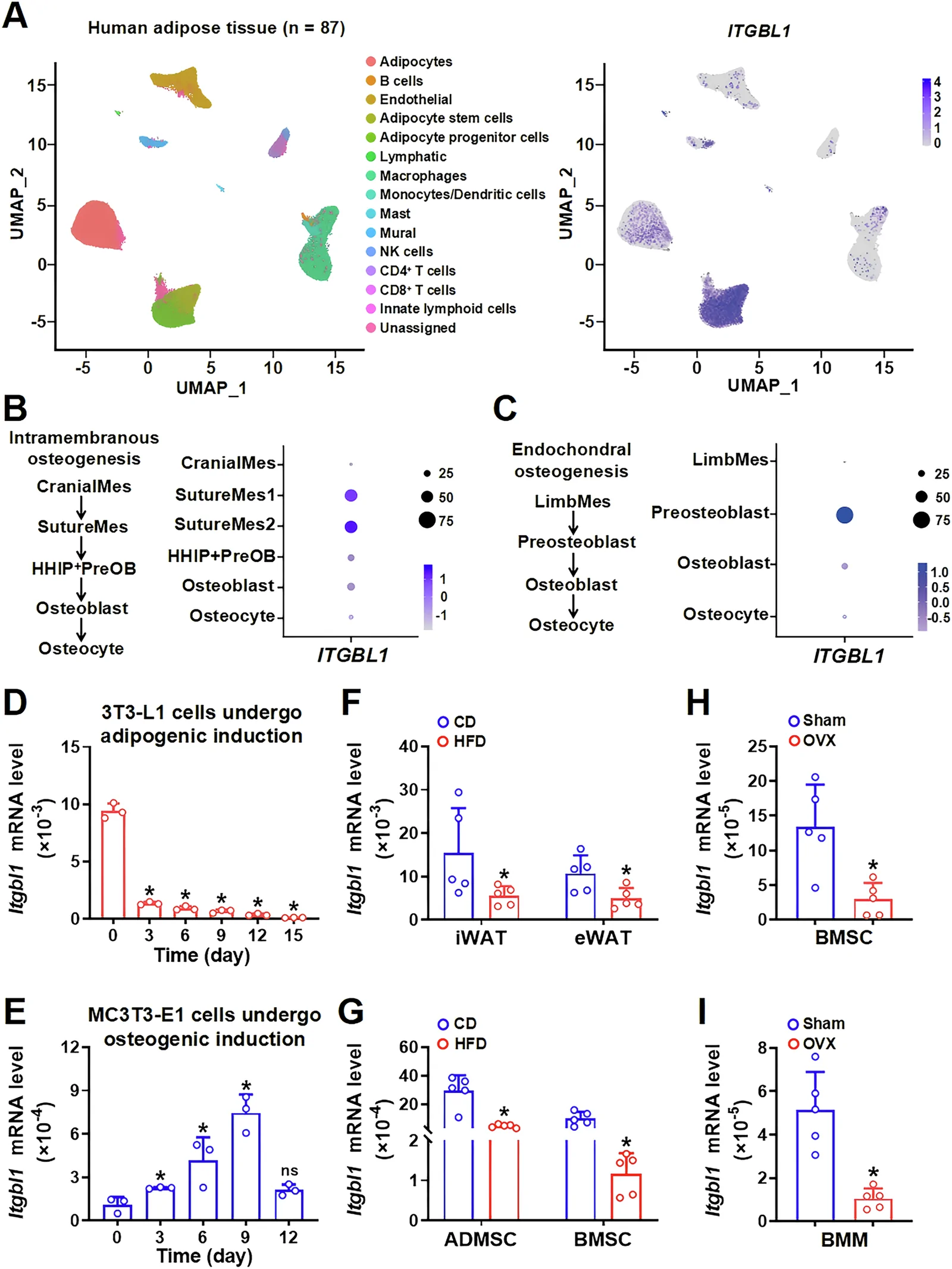
ITGBL1 is associated with adipogenic and osteogenic differentiation and is downregulated in obesity- and osteoporosis-related lineage imbalance. **A** The cellular composition of human adipose tissue cells (145,452 single nucleus obtained 74 samples from the Miranda AMA et al. published cohort and 13 samples from the Emont MP et al. published cohort) was conducted using Uniform Manifold Approximation and Projection (UMAP) and the visualization of *ITGBL1* enrichment was performed in the indicated cell populations. The snRNA–seq datasets comprising 12,095 nuclei from intramembranous bone-forming niches and 12,219 nuclei from endochondral bone-forming niches derived from 12 human embryonic donors spanning 5–11 post-conception weeks were analysed to visualize *ITGBL1* expression across cell populations during intramembranous (**B**) and endochondral (**C**) osteogenesis. CranialMes, cranial progenitors; SutureMes, suture mesenchyme; HHIP^+^PreOB, osteoprogenitors; LimbMes, limb mesenchyme. **D** *Itgbl1* mRNA levels in 3T3-L1 cells at the indicated time points during adipogenic differentiation induction measured by RT‒qPCR. **E** *Itgbl1* mRNA levels in MC3T3-E1 cells at the indicated time points during osteogenic differentiation induction measured by RT‒qPCR. *Itgbl1* mRNA levels in iWAT and eWAT (**F**) or ADMSCs and BMSCs (**G**) derived from mice fed a CD or HFD (each sample was pooled from two mice) were measured by RT‒qPCR (*n*  =  5 per group). *Itgbl1* mRNA levels in BMSCs (**H**) and BMMs (**I**) derived from sham or OVX mice (each sample was pooled from two mice) were measured by RT‒qPCR (*n*  =  5 per group). The data are presented as the mean ± SD. Statistical analyses were performed with the unpaired Student’s *t* test. *P < 0.05 compared with the control.

To define the lineage association of ITGBL1, we first analysed public single-cell transcriptomic datasets from human adipose and skeletal tissues. The analysis of an integrated mononuclear transcriptome dataset comprising 145,452 cells from 87 human adipose tissue revealed that *ITGBL1* was predominantly enriched in adipose stem cells and adipocyte progenitor cells, with limited expression in mature adipocytes and other non-mesenchymal cell populations (Fig. 1A). The analysis of snRNA–seq datasets comprising 12,095 nuclei from intramembranous bone-forming niches and 12,219 nuclei from endochondral bone-forming niches derived from 12 human embryonic donors spanning 5–11 post-conception weeks revealed that *ITGBL1* initially increased but then decreased during both intramembranous and endochondral osteogenesis (Fig. 1B, C), suggesting that ITGBL1 is closely associated with stem/progenitor cell states involved in adipogenic and osteogenic lineage commitment. We next examined the dynamic expression of *Itgbl1* during in vitro adipogenic and osteogenic differentiation. During adipogenic induction in 3T3-L1 cells, *Itgbl1* mRNA expression progressively decreased (Fig. 1D). During osteogenic induction in MC3T3-E1 cells, *Itgbl1* mRNA expression initially increased but then decreased over time (Fig. 1E). These results indicate that ITGBL1 is closely involved in both adipogenic and osteogenic differentiation processes.

To further assess the relationship between ITGBL1 expression and lineage imbalance associated with obesity and osteoporosis, we first established a diet-induced obesity model by feeding male C57BL/6 J mice a HFD beginning at 4 weeks of age, with mice fed a CD serving as controls. After 16 weeks, we measured the mRNA levels of *Itgbl1* in adipose tissue and MSCs. Surprisingly, the *Itgbl1* mRNA level was lower in the inguinal white adipose tissue (iWAT), epididymal white adipose tissue (eWAT), ADMSCs, and BMSCs of obese mice than in those of control mice (Fig. 1F, G). We next established an osteoporosis model by performing OVX on 12-week-old female mice, with sham-operated mice serving as controls. After 16 weeks, *Itgbl1* mRNA levels in primary BMSCs and BMMs isolated from OVX mice were significantly lower than those from sham mice (Fig. 1H, I). Together, these results indicate that ITGBL1 is closely associated with the regulation of adipogenic and osteogenic lineages and that its decreased expression is strongly linked to obesity- and osteoporosis-associated disruption of mesenchymal lineage homoeostasis.

### ITGBL1 deficiency enhances‌ adipogenic differentiation and inhibits osteogenic differentiation potential of MSCs and precursor cells

**Fig. 2:**
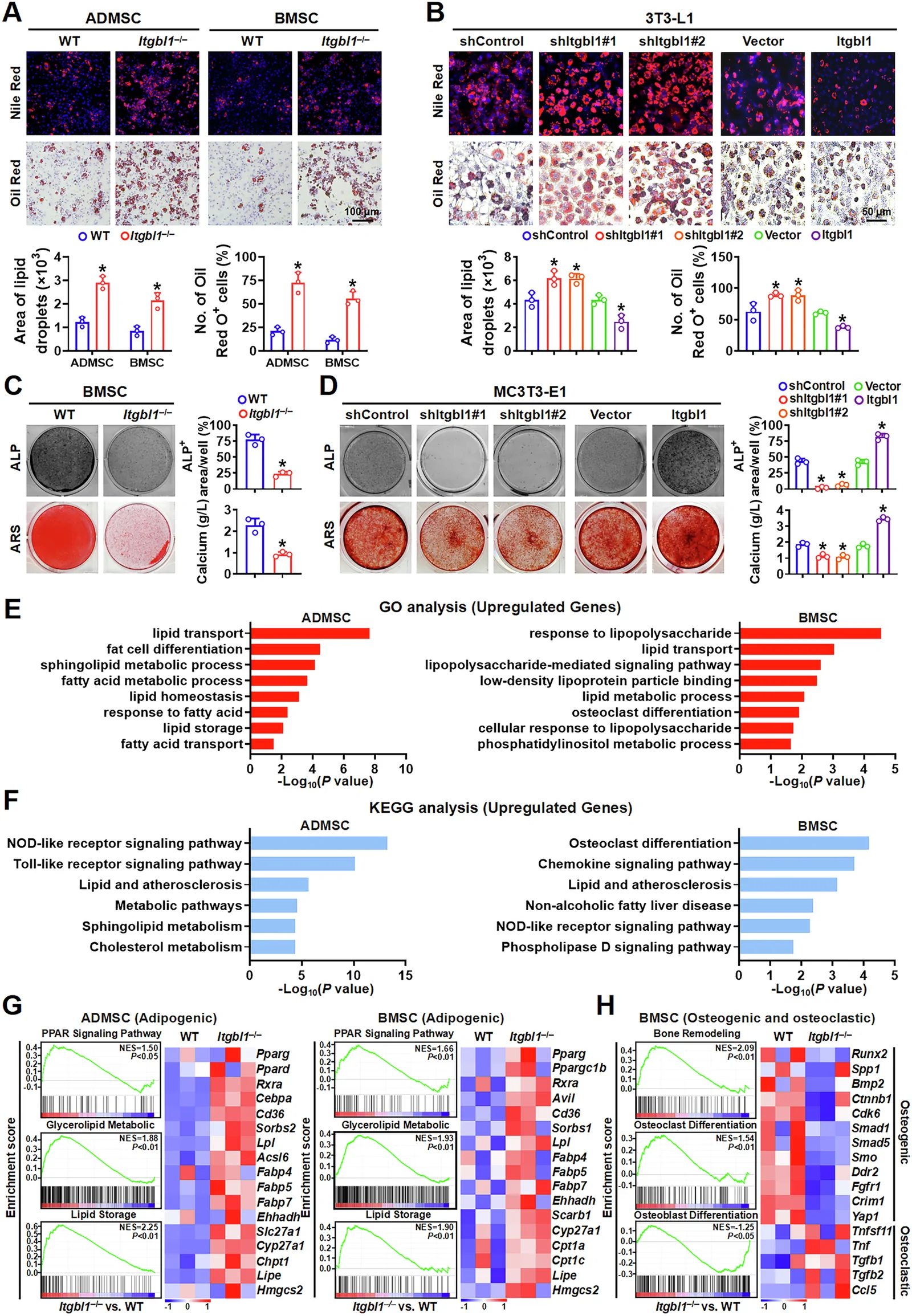
ITGBL1 deficiency enhances‌ adipogenic differentiation and inhibits osteogenic differentiation potential of MSCs and precursor cells. ADMSCs and BMSCs derived from WT and *Itgbl1*^*–/–*^ mice (**A**) and the indicated 3T3-L1 cells (**B**), were induced to undergo adipogenic differentiation. Representative images and quantitative analyses of Nile red and Oil Red O staining in MSCs and 3T3-L1 cells after adipogenic induction are shown. The lipid droplet area was quantified based on Nile Red staining, and the percentage of Oil Red O-positive cells was quantified based on Oil Red O staining. BMSCs derived from WT and *Itgbl1*^*–/–*^ mice (**C**) as well as the indicated MC3T3-E1 cells (**D**) were induced to undergo osteogenic differentiation. Representative images and quantitative analyses of ALP and ARS staining after osteogenic induction are shown. The ALP positive staining area was quantified, and calcium deposition was quantified based on ARS staining intensity. **E**, **F** ADMSCs and BMSCs derived from WT and *Itgbl1*^*–/–*^ mice were subjected to RNA-seq. The enriched functions and signalling pathways of the genes upregulated upon ITGBL1 deletion were analysed by GO (**E**) and KEGG (**F**) analyses. **G**, **H** Differentially enriched gene sets between the MSCs of WT and *Itgbl1*^–/–^ mice were identified by GSEA, and the corresponding representative genes were plotted as heatmaps. PPAR Signalling Pathway, Glycerolipid Metabolic, and Lipid Storage gene signatures were upregulated in ITGBL1-deficient ADMSCs and BMSCs (**G**). Bone Remodeling and Osteoclast Differentiation gene signatures were upregulated and Osteoblast Differentiation gene signatures were downregulated in ITGBL1-deficient BMSCs (**H**). NES: normalized enrichment score; FDR: false discovery rate. The data are presented as the mean ± SD. Statistical analyses were performed with the unpaired Student’s *t* test. *P < 0.05 compared with the control.

To further investigate the effects of ITGBL1 deficiency on the osteogenic and adipogenic differentiation balance of MSCs and precursor cells, we generated *Itgbl1*^−/−^ mice and isolated ADMSCs and BMSCs (Supplementary Fig. 1A, B) to compare adipogenic and osteogenic differentiation potential with that of cells from WT mice. After adipogenic induction, the increased Nile red staining intensity and number of Oil Red O-positive cells indicated a marked increase in lipid droplet accumulation in the ITGBL1-deficient ADMSCs and BMSCs (Fig. 2A). We then stably knocked down or overexpressed ITGBL1 in 3T3-L1 preadipocytes and MC3T3-E1 preosteoblasts (Supplementary Fig. 1C, D). Similarly, ITGBL1 depletion led to greater lipid accumulation in 3T3-L1 cells after adipogenic induction (Fig. 2B). Osteogenic differentiation and mineralization were severely impaired in the ITGBL1-deficient BMSCs and MC3T3-E1 cells after osteogenic induction (Fig. 2C, D). Conversely, ITGBL1 overexpression had the opposite effect on 3T3-L1 and MC3T3-E1 cells (Fig. 2B, D). In summary, ITGBL1 deficiency enhances the adipogenic potential of ADMSCs, BMSCs, and precursor cells while decreasing the osteogenic potential of BMSCs and precursor cells.

To elucidate the underlying mechanism by which ITGBL1 regulates osteogenic and adipogenic differentiation of MSCs, we performed RNA-seq on ADMSCs and BMSCs derived from WT and *Itgbl1*^*–/–*^ mice and analysed the differentially expressed genes using GO and KEGG enrichment analyses. GO analysis revealed that upregulated genes (log_2_FC ≥ 1 and *P* < 0.05) in ITGBL1-deficient ADMSCs and BMSCs were significantly enriched in biological processes related to adipogenic differentiation and lipid metabolism. Genes involved in osteoclastic differentiation were also enriched in the ITGBL1-deficient BMSCs (Fig. 2E). KEGG analysis revealed that the loss of ITGBL1 in ADMSCs and BMSCs activated mainly inflammatory (Toll-like receptor and NOD-like receptor) and lipid metabolism-related signalling pathways, and osteoclast differentiation-related factors were consistently enriched in ITGBL1-deficient BMSCs (Fig. 2F). GSEA confirmed that the PPAR Signalling Pathway, Glycerolipid Metabolic, and Lipid Storage gene signatures were upregulated in the ITGBL1-deficient ADMSCs and BMSCs (Fig. 2G), whereas Osteoblast Differentiation gene signatures were downregulated along with the Bone Remodeling and Osteoclast Differentiation gene signatures were upregulated in the ITGBL1-deficient BMSCs (Fig. 2H). In addition, genes involved in osteogenic and osteoclastic differentiation were not consistently altered in the ITGBL1-deficient ADMSCs (Supplementary Fig. 1E). RT**‒**qPCR validation revealed that the expression levels of markers related to adipogenic differentiation and lipid metabolism (*Pparg*, *Cebpa*, *Cd36*, *Fabp5*, *Fabp7*, and *Lpl*) were significantly upregulated in ADMSCs and BMSCs derived from *Itgbl1*^–/–^ mice (Supplementary Fig. 2A, B), confirming that the adipogenic potential of MSCs is enhanced by ITGBL1 depletion. Moreover, decreased mRNA levels of osteogenic markers (*Bmp2*, *Runx2*, and *Spp1*) and increased mRNA levels of osteoclastogenic cytokines (*Tnfsf11*, *Tgfb1*, and *Tgfb2*) in ITGBL1-deficient BMSCs confirmed that the osteogenic potential of these cells was attenuated, while their potential for promoting osteoclast differentiation was enhanced (Supplementary Fig. 2C, D). Furthermore, we confirmed that ITGBL1 negatively regulates the expression of genes related to adipogenesis in 3T3-L1 cells (Supplementary Fig. 2E) but positively regulates the expression of genes related to osteogenic differentiation in MC3T3-E1 cells (Supplementary Fig. 2F). These results demonstrate that ITGBL1 plays a critical role in suppressing the adipogenic potential of ADMSCs, BMSCs, and precursor cells while enhancing the osteogenic potential of BMSCs and precursor cells. Additionally, the modulation of BMSC differentiation by ITGBL1 can suppress osteoclast differentiation. Therefore, ITGBL1 deficiency enhances‌ adipogenic differentiation and inhibits osteogenic differentiation potential of MSCs and precursor cells.

### ITGBL1 promotes the ubiquitination and proteasomal degradation of PPARγ

**Fig. 3:**
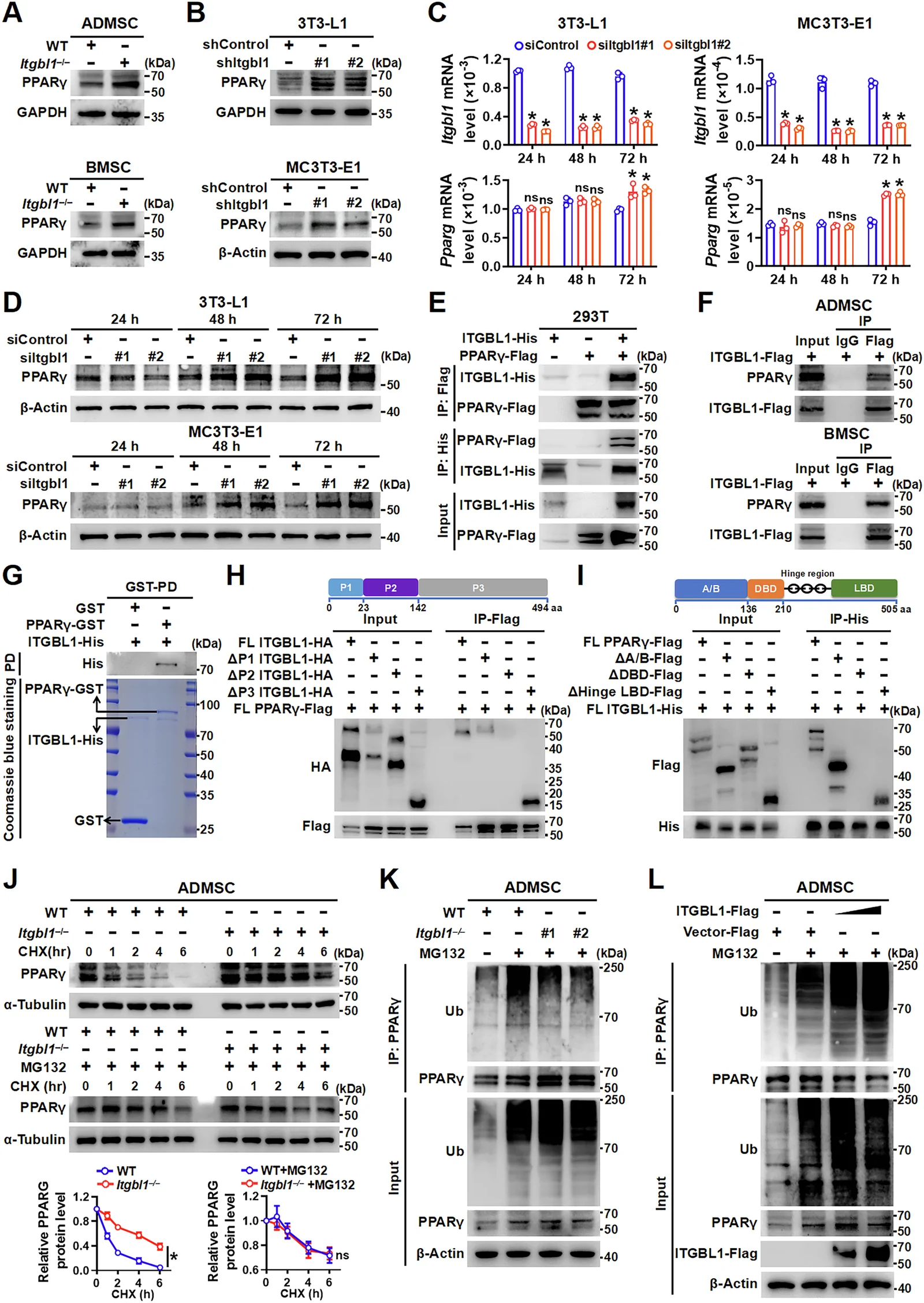
ITGBL1 facilitates the ubiquitin-mediated degradation of PPARγ through interactions with PPARγ. **A** PPARγ protein levels in ADMSCs and BMSCs from WT and *Itgbl1*^–/–^ mice were analysed by IB. **B** 3T3-L1 and MC3T3-E1 cells were stably transfected with shItgbl1 or shControl via lentivirus infection. PPARγ protein levels in the indicated cells were analysed by IB. **C**, **D** 3T3-L1 and MC3T3-E1 cells were transiently transfected with siItgbl1 or siControl and collected at 24, 48 or 72 h after transfection. The mRNA (**C**) and protein (**D**) levels of PPARγ in the indicated cells were measured by RT–qPCR and IB, respectively. **E** HEK293T cells were transiently transfected with plasmids expressing either ITGBL1-His or PPARγ-Flag alone or in combination, after which the interaction between ITGBL1 and PPARγ was detected by co-IP and IB with the indicated antibodies. **F** ADMSCs and BMSCs were infected with lentivirus carrying ITGBL1-Flag, and the interaction between ITGBL1 and PPARγ was detected by co-IP and IB with the indicated antibodies. **G** ITGBL1-His and PPARγ-GST expressed in bacteria were purified and used for a GST-PD assay. Interactions between the two proteins were detected by IB with an anti-His antibody. The expression of the input GST, PPARγ-GST, and ITGBL1-His proteins was determined by Coomassie blue staining. **H** Plasmids expressing the FL ITGBL1-HA or ITGBL-HA mutants lacking P1 (ΔP1 ITGBL1-HA), P2 (ΔP2 ITGBL1-HA), or P3 (ΔP3 ITGBL1-HA) were cotransfected with the PPARγ-Flag-expressing plasmid in HEK293T cells. The interaction between the two proteins was detected by co-IP and IB with the indicated antibodies. **I** Plasmids expressing FL PPARγ-Flag or PPARγ-Flag mutants lacking AF1 (ΔAF1 PPARγ-Flag), DBD (ΔDBD PPARγ-Flag), or Hin-LBD (ΔHin-LBD PPARγ-Flag) were cotransfected with a plasmid expressing ITGBL1-His in HEK293T cells. The interaction between the two proteins was detected by co-IP and IB with the indicated antibodies. **J** WT and *Itgbl1*^–/–^ ADMSCs were treated with 20 μM cycloheximide (CHX) for the indicated times, with or without 10 μM MG132 for the first 6 h. PPARγ protein levels in the indicated cells were analysed by IB with an anti-PPARγ antibody. WT and *Itgbl1*^–/–^ ADMSCs (**K**) or WT ADMSCs transfected with different amounts of ITGBL1-Flag (**L**) were treated with 10 μM MG132 for 6 h. The ubiquitination of PPARγ in the indicated cells was analysed by IP with an anti-PPARγ antibody and IB with an anti-ubiquitin antibody. The data are shown as the mean ± SD. Statistical analyses were performed with the unpaired Student’s *t* test (**C**) or two-way ANOVA (**J**). *P < 0.05 compared with the control. ns, not statistically significant.

Given that GSEA of the RNA-seq data demonstrated significant activation of the PPAR signalling pathway in ITGBL1-deficient ADMSCs and BMSCs, among which the expression of PPARγ and its downstream effector molecules was upregulated (Fig. 2G, and Supplementary Fig. 2A, B), we further examined the protein expression of PPARγ and confirmed that ITGBL1 consistently modulates PPARγ protein levels in these cells as well as in precursor cells (Fig. 3A, B). To investigate the mechanism underlying the ITGBL1-mediated regulation of PPARγ, we transiently transfected 3T3-L1 and MC3T3-E1 cells with siItgbl1 and found that *Pparg* mRNA levels in both cell lines were increased beginning 72 h after ITGBL1 knockdown (Fig. 3C), whereas PPARγ protein levels were increased significantly beginning 48 h after ITGBL1 knockdown (Fig. 3D). These observations suggest that ITGBL1 regulates PPARγ expression primarily through posttranslational modifications rather than transcriptional regulation.

To investigate whether a specific interaction occurs between ITGBL1 and PPARγ, co-IP assays were performed with exogenous ITGBL1 and either endogenous or exogenous PPARγ in 293 T cells (Supplementary Fig. 3A, and Fig. 3E). Indeed, we observed a clear interaction between ITGBL1 and PPARγ. Furthermore, endogenous PPARγ was detectable in exogenous ITGBL1 immunoprecipitates originating from ADMSCs and BMSCs (Fig. 3F), and this interaction was observed in both the cytoplasm and nucleus (Supplementary Fig. 3B). An in vitro GST-PD assay using purified recombinant proteins demonstrated that ITGBL1 directly interacted with PPARγ (Fig. 3G). To further investigate the interaction domains of ITGBL1 and PPARγ, the ITGBL1 protein was divided into three domains: P1 (0–23 aa, signal peptide), P2 (24–142 aa), and P3 (143–494 aa, a protein isoform with an independent promoter), and the PPARγ protein was divided into the N-terminal A/B domain containing activation function 1, the DNA-binding domain (DBD), and the C-terminal hinge ligand binding domain (LBD). Plasmids expressing each protein with domain deletion were constructed, and co-IP assays were performed in 293 T cells via the cotransfection of FL ITGBL1 and mutant PPARγ or FL PPARγ and mutant ITGBL1 (Fig. 3H, I). The results revealed that mutant ITGBL1 lacking the P2 domain failed to bind FL PPARγ (Fig. 3H), and mutant PPARγ lacking the DBD failed to bind FL ITGBL1 (Fig. 3I), indicating that the P2 domain of ITGBL1 is required for direct interaction with the DBD of PPARγ.

To further investigate whether the stability of PPARγ protein is affected by ITGBL1 via protein‒protein interactions, we first examined PPARγ protein levels in the presence of cycloheximide (CHX), a protein translation inhibitor, in ADMSCs derived from WT and *Itgbl1*^–/–^ mice and found that the stability of endogenous PPARγ was significantly greater in *Itgbl1*^–/–^ADMSCs than in WT ADMSCs (Fig. 3J). Furthermore, when the proteasome inhibitor MG132 was added to these cells, the stability of endogenous PPARγ was no longer influenced by ITGBL1 deficiency (Fig. 3J), indicating that the effect of ITGBL1 on PPARγ stability is mediated through ubiquitination and proteasomal degradation. We further performed ubiquitination assays in 293 T cells, and the results revealed that ITGBL1 overexpression increased the levels of polyubiquitinated endogenous and exogenous PPARγ (Supplementary Fig. 3C, D). Consistently, the polyubiquitination level of PPARγ in *Itgbl1*^–/–^ ADMSCs was lower than that in WT ADMSCs (Fig. 3K), and ITGBL1 overexpression in mouse ADMSCs increased the polyubiquitination level of PPARγ (Fig. 3L). These results indicate that ITGBL1 interacts with PPARγ to promote its ubiquitination and proteasomal degradation.

**Fig. 4:**
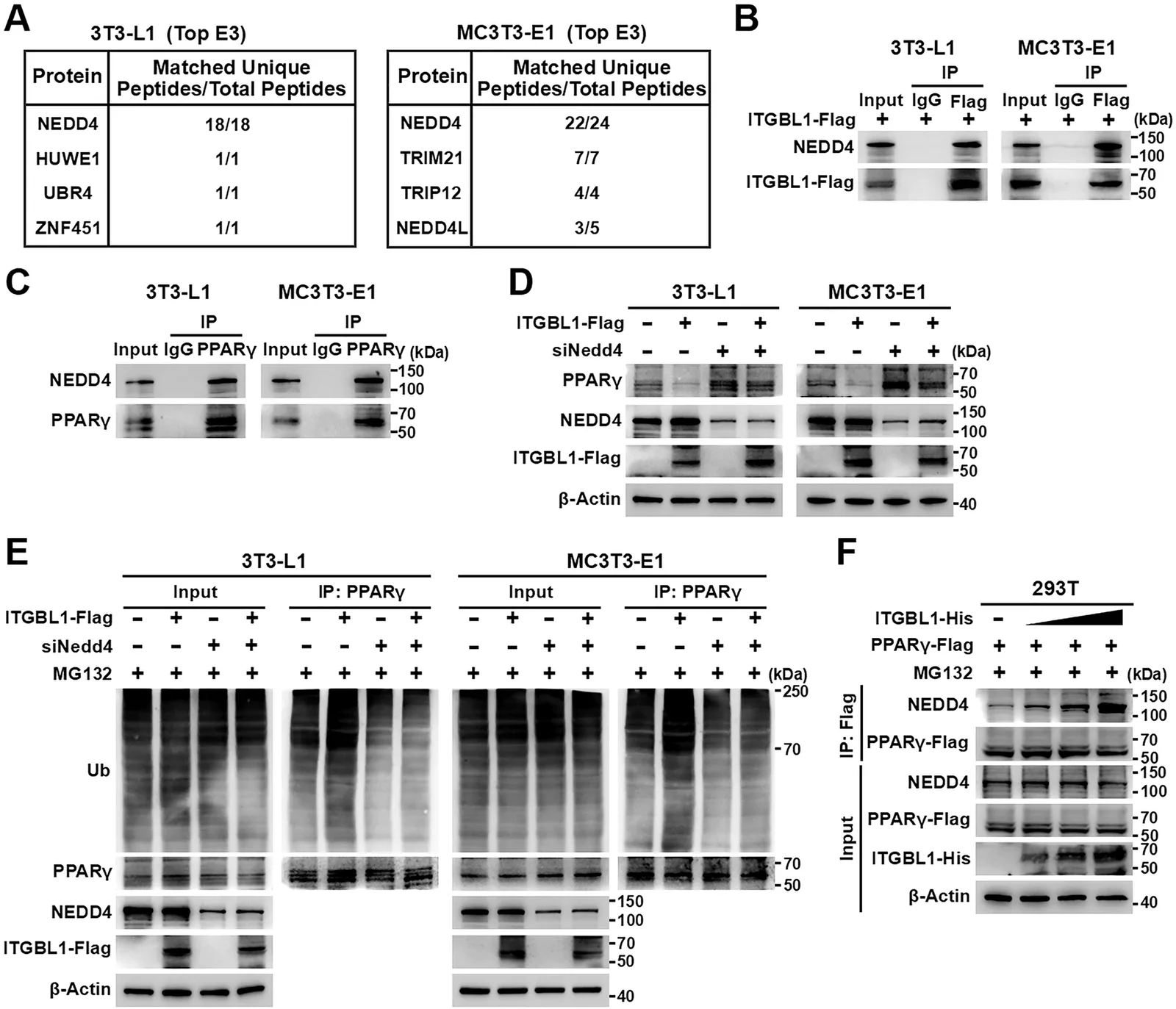
ITGBL1 interacts with NEDD4 and enhances NEDD4-mediated ubiquitination and proteasomal degradation of PPARγ. **A** E3 ubiquitin ligases interacting with ITGBL1 in 3T3-L1 and MC3T3-E1 cells were identified by co-IP followed by LC‒MS/MS analysis. **B** 3T3-L1 and MC3T3-E1 cells were infected with lentivirus carrying ITGBL1-Flag, and the interaction between ITGBL1 and NEDD4 was detected by co-IP and IB with the indicated antibodies. **C** The interaction between PPARγ and NEDD4 in the indicated cells was detected by co-IP and IB with the indicated antibodies. **D** 3T3-L1 and MC3T3-E1 cells overexpressing ITGBL1 via lentivirus infection and/or with NEDD4 knockdown by siRNA transfection were analysed for the protein levels of PPARγ, NEDD4, and ITGBL1 by IB. **E** PPARγ ubiquitination levels in the indicated cells were detected by IP and IB after MG132 treatment. **F** 293 T cells were cotransfected with Flag-tagged PPARγ and different amounts of His-tagged ITGBL1 plasmid or vector control. The protein levels of NEDD4 interacting with PPARγ were detected by co-IP and IB. The data are presented as the mean ± SD. Statistical analyses were performed with the unpaired Student’s *t* test. *P < 0.05 compared with the control.

We further identified potential ubiquitin ligases for PPARγ ubiquitination regulated by ITGBL1 through co-IP and LC‒MS/MS analyses in 3T3-L1 and MC3T3-E1 cells. Interestingly, NEDD4 was presented on the prey list in both 3T3-L1 and MC3T3-E1 cells (Fig. 4A) and that NEDD4-mediated ubiquitination and degradation of PPARγ through direct protein interaction has already been reported [32]. To investigate whether NEDD4 mediates the ITGBL1-regulated ubiquitination and degradation of PPARγ, the interactions between NEDD4 and ITGBL1 or PPARγ were validated by co-IP and IB (Fig. 4B, C), indicating that ITGBL1, PPARγ, and NEDD4 can form a complex. Further validation revealed that NEDD4 knockdown significantly reversed the ITGBL1-induced reduction in PPARγ protein levels and increase in PPARγ ubiquitination in 3T3-L1 and MC3T3-E1 cells overexpressing ITGBL1 (Fig. 4D, E, and Supplementary Fig. 4). Furthermore, co-IP experiments revealed that ITGBL1 increased binding between PPARγ and NEDD4 in a dose-dependent manner in 293 T cells (Fig. 4F). Taken together, these data indicate that ITGBL1 reduces PPARγ protein abundance by facilitating NEDD4-dependent ubiquitination of PPARγ, thereby promoting its proteasomal degradation.

### Mutual transcriptional regulation among ITGBL1, PPARγ and RUNX2 in cells of osteogenic lineage and adipogenic lineage

**Fig. 5:**
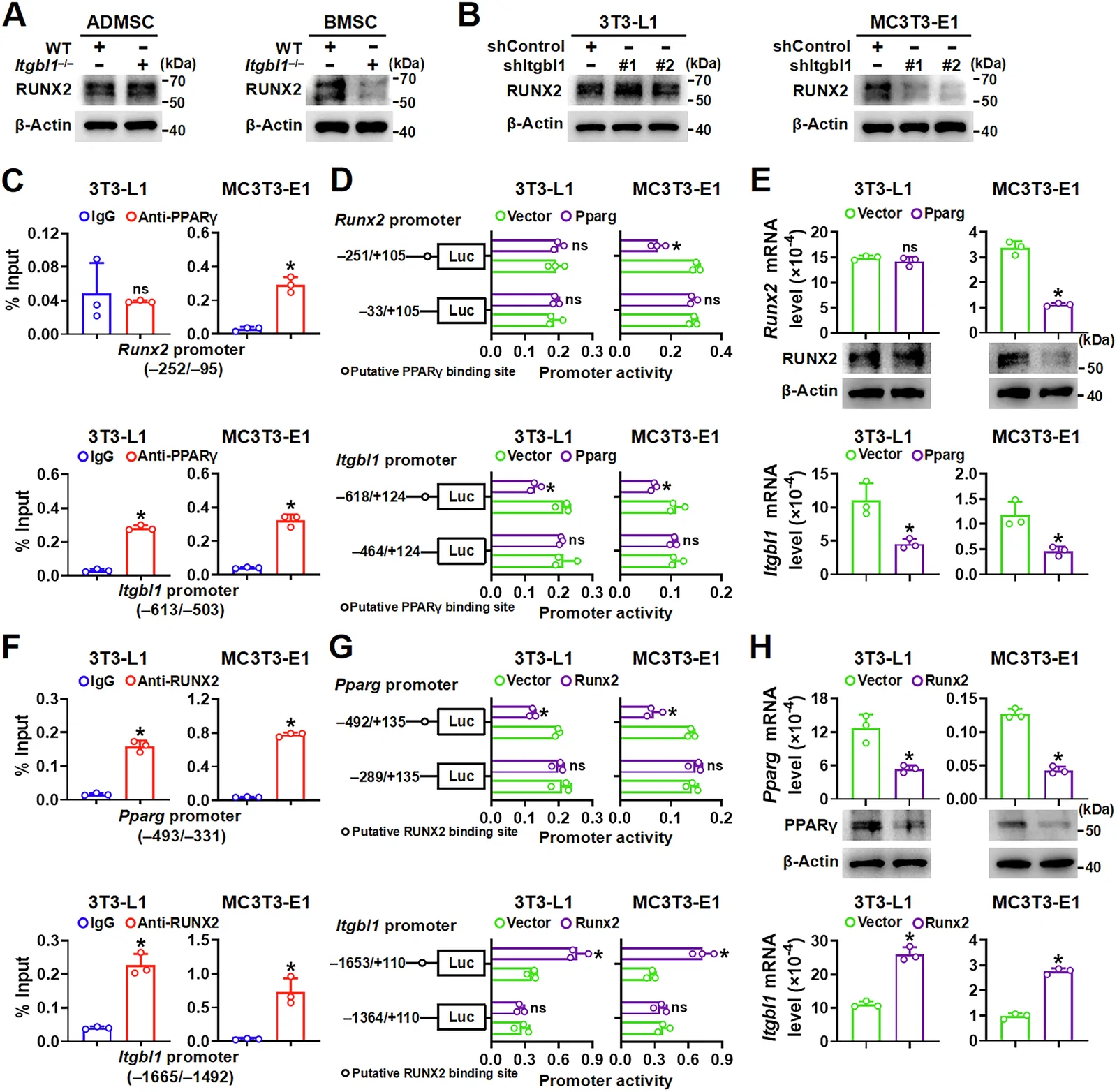
Mutual transcriptional regulation among ITGBL1, PPARγ and RUNX2 in cells of osteogenic lineage and adipogenic lineage. **A** RUNX2 protein levels in ADMSCs and BMSCs from WT and *Itgbl1*^–/–^ mice were analysed by IB. **B** RUNX2 protein levels in the indicated MC3T3-E1 and 3T3-L1 cells were analysed by IB. **C** Enrichment of PPARγ on the *Runx2* and *Itgbl1* proximal promoter regions in the indicated cells was assessed by ChIP–qPCR. **D** Activation of the *Runx2* or *Itgbl1* promoter, with or without the putative PPARγ binding site, in the indicated cells was assessed using a dual-luciferase reporter assay. **E** The mRNA and protein levels of RUNX2 and ITGBL1 in the indicated cells were detected by RT‒qPCR and IB, respectively. **F** Enrichment of RUNX2 on the *Pparg* or *Itgbl1* proximal promoter region in the indicated cells was assessed by ChIP–qPCR. **G** Activation of the *Pparg* or *Itgbl1* promoter with or without the putative RUNX2 binding site in the indicated cells was assessed using a dual-luciferase reporter assay. **H** The mRNA and protein levels of PPARγ and ITGBL1 in the indicated cells were detected by RT‒qPCR and IB, respectively. The data are presented as the mean ± SD. Statistical analyses were performed with the unpaired Student’s *t* test. *P < 0.05 compared with the control. ns, not statistically significant.

An imbalance in the differentiation of MSCs into osteogenic and adipogenic lineages is a primary contributor to obesity and osteoporosis. RUNX2 and PPARγ are the core transcription factors that regulate osteogenic and adipogenic differentiation, respectively, and their expression is inversely related during MSC differentiation [15, 19]. Indeed, we observed that RUNX2 and PPARγ expression levels were altered in opposite ways in ITGBL1-deficient BMSCs and MC3T3-E1 cells (Fig. 2G, H, and Supplementary Fig. 2B, C, and Fig. 3A, B, and Fig. 5A, B). However, in contrast to the regulation of PPAR**γ** by ITGBL1, ITGBL1 did not regulate RUNX2 in ADMSCs or 3T3-L1 cells (Supplementary Fig. 1E, and Fig. 5A, B). Owing to the presence of a putative PPARγ-binding element within the 2-kb proximal promoter region relative to the transcriptional start site (TSS) of *Runx2* and *Itgbl1* and a putative RUNX2-binding element within the 2-kb proximal promoter region relative to the TSS of *Pparg* and *Itgbl1* (Supplementary Fig. 5A, B), we further explored the transcriptional regulation among PPARγ, RUNX2, and ITGBL1 in 3T3-L1 and MC3T3-E1 cells. ChIP‒qPCR, luciferase reporter, RT–qPCR, and IB assays revealed that PPARγ bound to the proximal promoter region of *Runx2* and negatively regulated its transcription and expression in MC3T3-E1 cells but not in 3T3-L1 cells, whereas PPARγ bound to the proximal promoter region of *Itgbl1* and directly repressed its transcription and expression in both 3T3-L1 and MC3T3-E1 cells (Fig. 5C–E, and Supplementary Fig. 5C, D). RUNX2 also bound to the proximal promoter regions of *Pparg* and *Itgbl1*, negatively regulating the transcription and expression of *Pparg* but positively regulating the transcription and expression of *Itgbl1* in both 3T3-L1 and MC3T3-E1 cells (Fig. 5F–H, and Supplementary Fig. 5E, F). These results demonstrate that PPARγ and RUNX2 regulate the balance between osteogenic and adipogenic differentiation through reciprocal transrepression and that both factors have opposite effects on the transcriptional regulation of *Itgbl1* in osteogenic-lineage and adipogenic-lineage precursor cells. Therefore, ITGBL1 deficiency initiates lipid metabolism reprogramming by activating PPARγ, which in turn represses *Runx2* and *Itgbl1* in specific cellular contexts, exacerbating the imbalance between osteogenic and adipogenic differentiation.

### PPARγ mediates the gain of adipogenic differentiation and the loss of osteogenic differentiation of ITGBL1-deficient MSCs and precursor cells

**Fig. 6:**
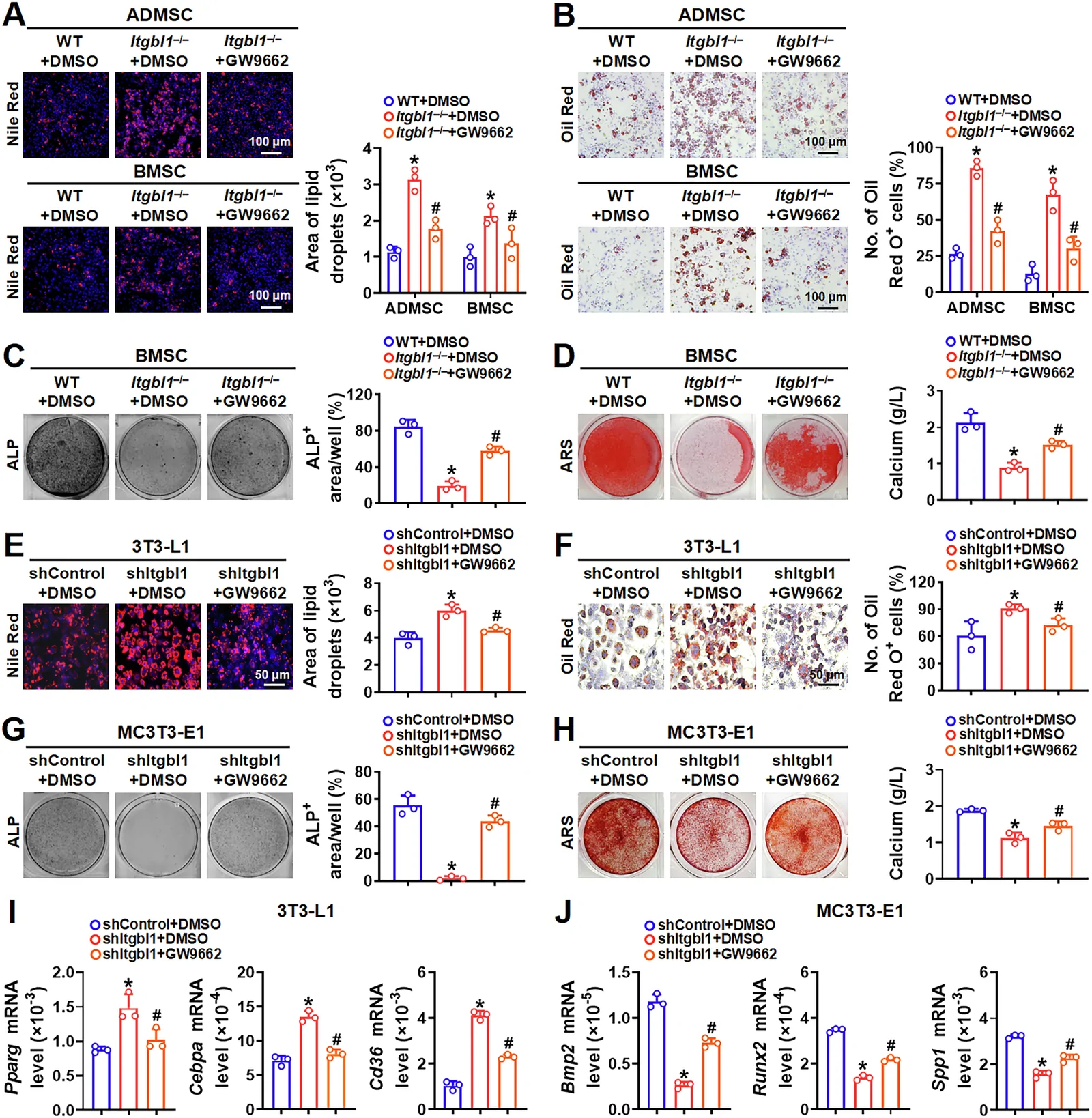
PPARγ mediates the gain of adipogenic differentiation and the loss of osteogenic differentiation of ITGBL1-deficient MSCs and precursor cells. **A**–**D** ADMSCs and BMSCs derived from *Itgbl1*^*–/–*^ mice were treated with the PPARγ-specific antagonist GW9662 at a concentration of 4 μM during adipogenic and osteogenic induction, respectively. Representative images and quantitative analyses of Nile red (**A**) and Oil Red O (**B**) staining for the indicated ADMSCs and BMSCs after adipogenic induction. Representative images and quantitative analyses of ALP (**C**) and ARS (**D**) staining for the indicated BMSCs after osteogenic induction. **E‒J** 3T3-L1 and MC3T3-E1 cells transfected with shItgbl1 or shControl were treated with 4 μM GW9662 during adipogenic or osteogenic induction. Representative images and quantitative analyses of Nile red (**E**) and Oil Red O (**F**) staining of 3T3-L1 cells after adipogenic induction. Representative images and quantitative analysis of ALP (**G**) and ARS (**H**) staining of MC3T3-E1 cells after osteogenic induction. The expression levels of *Pparg*, *Cebpa*, and *Cd36* in the indicated 3T3-L1 cells (**I**) as well as *Bmp2*, *Runx2*, and *Spp1* in the indicated MC3T3-E1 cells (**J**) were measured by RT–qPCR. The data are presented as the mean ± SD. Statistical analyses were performed with the unpaired Student’s *t* test. *P < 0.05 compared with the control cells. ^#^*P* < 0.05 compared with the cells with *Itgbl1*^*–/–*^ or shItgbl1.

To investigate whether PPARγ mediates the increased adipogenic differentiation and reduced osteogenic differentiation of ITGBL1-deficient MSCs and precursor cells, we treated ADMSCs and BMSCs derived from *Itgbl1*^*–/–*^ mice with the PPARγ-specific antagonist GW9662 during adipogenic and osteogenic induction. Nile red and Oil Red O staining revealed that the increased adipogenic differentiation of ITGBL1-deficient MSCs was inhibited by GW9662 treatment (Fig. 6A, B). ALP and ARS staining indicated that the suppressed osteogenic differentiation of the ITGBL1-deficient BMSCs was partially reversed by GW9662 treatment (Fig. 6C, D). In addition, we treated ITGBL1-knockdown 3T3-L1 and MC3T3-E1 cells with GW9662 during adipogenic and osteogenic induction, respectively. Similarly, the intensity of Nile red staining and the number of Oil red O-positive 3T3-L1 cells were decreased (Fig. 6E, F), whereas the intensity of ALP staining and the calcium salt content determined via ARS staining were increased in MC3T3-E1 cells (Fig. 6G, H). Moreover, GW9662 treatment downregulated the expression of adipogenic genes (*Pparg*, *Cebpa*, and *Cd36*) in ITGBL1-knockdown 3T3-L1 cells (Fig. 6I) and upregulated the expression of osteogenic genes (*Bmp2*, *Runx2*, and *Spp1*) in ITGBL1-knockdown MC3T3-E1 cells (Fig. 6J). These results demonstrate that increased PPARγ activity promotes the adipogenic differentiation and impairs the osteogenic differentiation of ITGBL1-deficient MSCs and precursor cells.

### ITGBL1 treatment alleviates HFD-induced obesity and osteoporosis

**Fig. 7:**
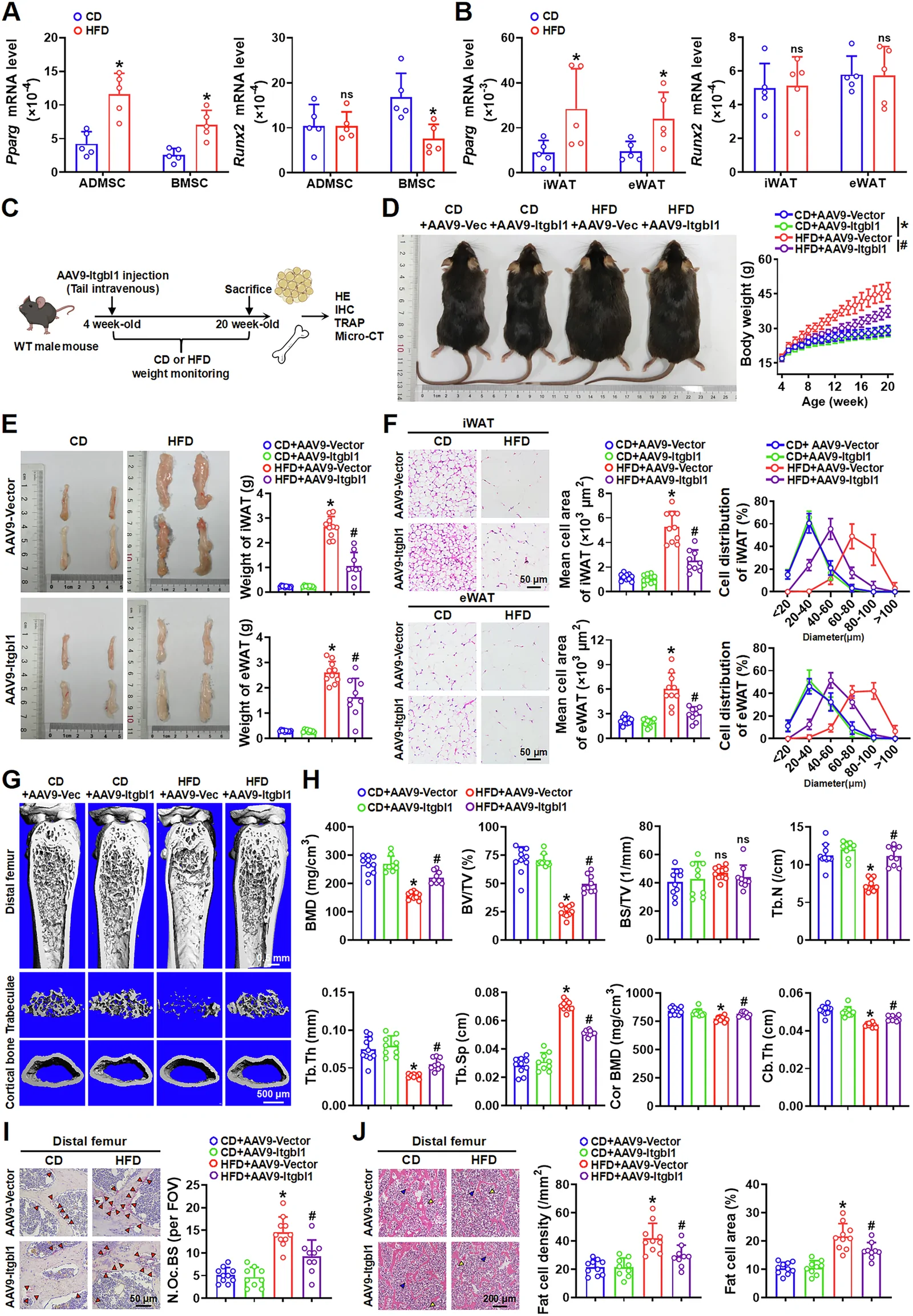
ITGBL1 treatment alleviates HFD-induced obesity and osteoporosis. WT male C57BL/6 J mice fed a CD or HFD were injected with AAV9-Itgbl1 or AAV9-Vector (2 × 10^11^ vg) via the tail vein at 4 weeks of age and allowed to grow to 20 weeks of age (*n*  =  10 per group). *Pparg* and *Runx2* mRNA levels in ADMSCs and BMSCs (**A**) or iWAT and eWAT (**B**) derived from mice fed a CD or HFD (each sample was pooled from two mice) were measured by RT‒qPCR (*n*  =  5 per group). **C** Schematic representation of the experimental design for AAV9- mediated ITGBL1 treatment in mice. **D** Representative images and body weight growth curves of the mice. **E** Representative images and weight quantification of iWAT and eWAT. **F** Representative H&E staining images and quantification of the adipocyte area and diameter distribution of iWAT and eWAT. **G** Representative micro-CT images of distal femurs, trabeculae and midshaft cortical bone. **H** Quantification of the BMD, BV/TV, Tb.N, Tb.Th, BS/TV, Tb.Sp, Cor-BMD, and Cb.Th at the distal femur and midshaft cortical bone by micro-CT analysis. **I** Representative TRAP staining images and quantification of osteoclast numbers in the femoral bone marrow. **J** Representative images and quantification of the number and area of adipocytes in the distal femoral bone marrow. The blue arrows indicate trabecular bone, and the yellow arrows indicate BMAT. The data are shown as the mean ± SD. Statistical analyses were performed with the unpaired Student’s *t* test or two-way ANOVA (**D**). *P < 0.05 compared with the control mice. ^#^*P* < 0.05 compared with the HFD-fed mice. ns, not statistically significant.

Given that reduced ITGBL1 expression was closely associated with diet-induced obesity and osteoporosis (Fig. 1F, G), we next examined the mRNA levels of *Pparg* and *Runx2* in MSCs and adipose tissue from diet-induced obese and osteoporotic mice. Notably, compared with those in the controls, the *Pparg* mRNA levels in the ADMSCs, BMSCs, iWAT, and eWAT from obese mice were increased, whereas the *Runx2* mRNA level was decreased only in the BMSCs and remained unchanged in the ADMSCs and adipose tissue of obese mice (Fig. 7A, B). These findings suggest that decreased ITGBL1 expression due to diet-induced obesity and osteoporosis is closely linked to disruption of the balance between osteogenic and adipogenic differentiation of MSCs.

We further tested whether ITGBL1 supplementation could ameliorate diet-induced obesity and its associated osteoporosis. To clarify in vivo biodistribution for systemic AAV9-mediated delivery of ITGBL1, we injected AAV9-GFP into the tail vein of 4-week-old mice, with PBS injection used as a control. Organs were collected after 2 and 16 weeks of administration, and GFP expression was assessed by IHC. After 2 and 16 weeks of AAV9-GFP injection, GFP-positive signals were observed in the iWAT, eWAT, distal femur, and bone marrow, indicating effective transduction of adipose- and bone-related tissues. In addition, weak GFP-positive signals were observed in liver and brain tissues but not in other organs (Supplementary Fig. 6).

Next, 4-week-old mice fed a CD or HFD were treated with AAV9-Itgbl1 or AAV9-Vector via tail vein injection and then allowed to grow until 20 weeks of age (Fig. 7C, and Supplementary Fig. 7A, B). As expected, ITGBL1 treatment significantly alleviated HFD-induced obesity (Fig. 7D) and accordingly reduced WAT weight (Fig. 7E) and unilocular lipid droplet size (Fig. 7F). Compared with CD-fed mice, HFD-induced obese mice exhibited marked bone loss (Fig. 7G), as evidenced by decreased trabecular BMD, BV/TV, Tb.N, and Tb.Th; increased Tb.Sp; and decreased Cor-BMD and Cb.Th (Fig. 7H), along with the accumulation of osteoclasts and BMAT in primary and secondary ossification centres (Fig. 7I, J). ITGBL1 treatment also significantly alleviated FD-induced osteoporosis (Fig. 7G), as reflected by increased trabecular BMD, BV/TV, Tb.N, and Tb.Th; decreased Tb.Sp; and increased Cor-BMD and Cb.Th (Fig. 7H). In parallel, ITGBL1 treatment reduced the accumulation of osteoclasts and BMAT in the bone marrow of HFD-fed obese mice (Fig. 7I, J). Moreover, compared with that in the CD-fed mice, ITGBL1 protein expression was decreased and PPARγ protein expression was increased in the iWAT and bone marrow tissue of the HFD-fed mice, whereas RUNX2 protein expression was decreased in the bone marrow tissue but remained unchanged in the iWAT of the HFD-fed mice (Supplementary Fig. 7A–D). ITGBL1 treatment restored the protein levels of PPARγ and RUNX2 in these tissues (Supplementary Fig. 7A, B). Importantly, ITGBL1 treatment did not significantly affect the above phenotypes in CD-fed mice (Fig. 7D–J). Collectively, these findings demonstrate that the HFD-induced downregulation of ITGBL1 in MSCs and WAT leads to obesity and osteoporosis and that ITGBL1 supplementation can effectively treat diet-induced obesity and osteoporosis.

### ITGBL1 treatment alleviates osteoporosis in OVX mice

**Fig. 8:**
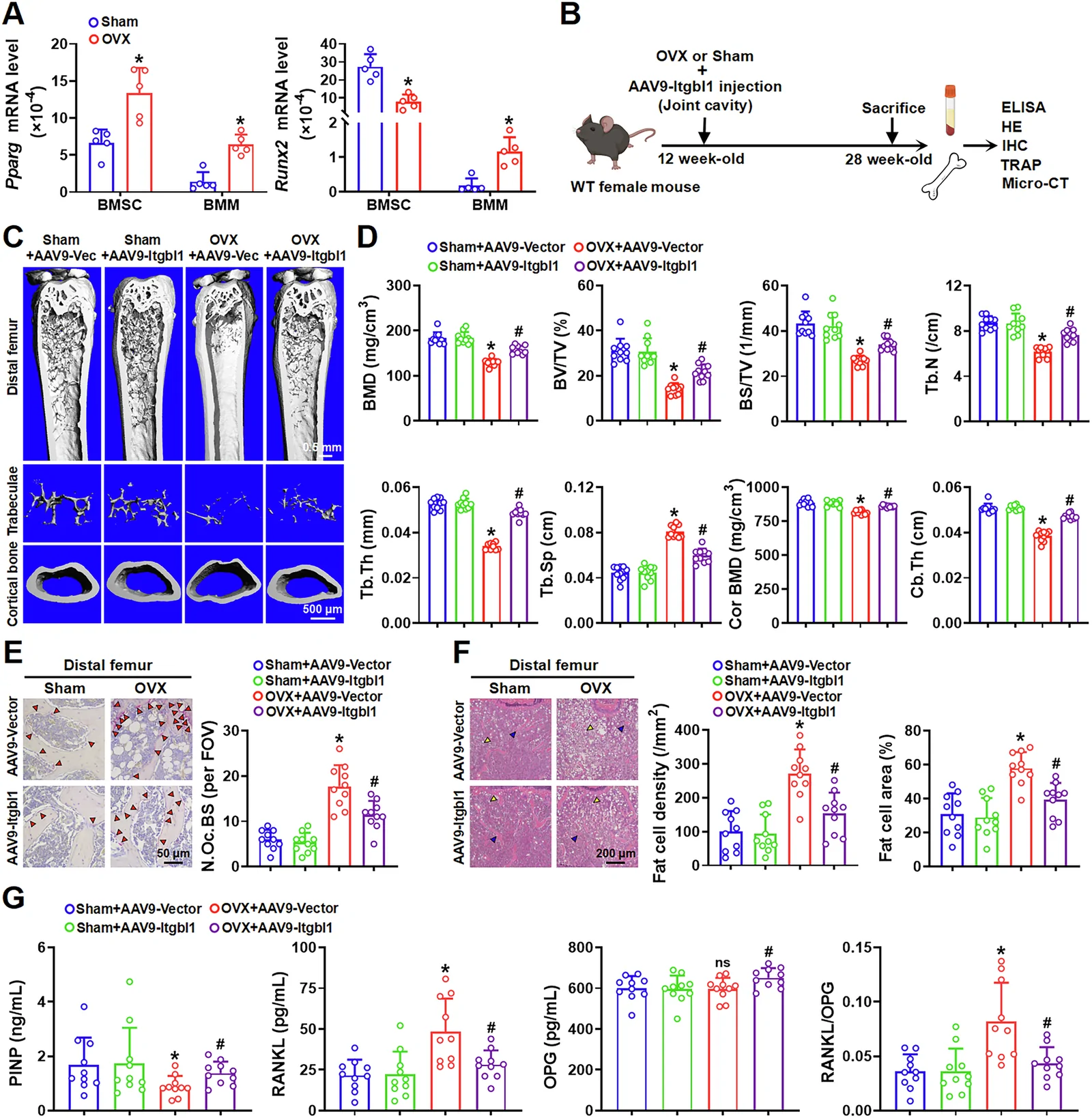
ITGBL1 treatment alleviates osteoporosis in OVX mice. WT female C57BL/6 J mice fed a CD underwent OVX or sham surgery and were injected with AAV9-Itgbl1 or AAV9-Vector (5 × 10^10^ vg) into the knee joint cavity at 12 weeks of age, then allowed to grow to 28 weeks of age (*n*  =  10 per group). **A** *Pparg* and *Runx2* mRNA levels in BMSCs and BMMs derived from the sham or OVX mice (each sample was pooled from two mice) were measured by RT‒qPCR (*n*  =  5 per group). **B** Schematic diagram of the experimental design for AAV9-Itgbl1 treatment in sham and OVX mice. **C** Representative micro-CT images of distal femurs, trabeculae and midshaft cortical bone. **D** Quantification of the BMD, BV/TV, Tb.N, Tb.Th, BS/TV, Tb.Sp, Cor-BMD, and Cb.Th at the distal femur and midshaft cortical bone by micro-CT analysis. **E** Representative TRAP staining images and quantification of osteoclast numbers in the femoral bone marrow. **F** Representative images and quantification of the number and area of adipocytes in the distal femoral bone marrow. Blue arrows indicate trabecular bone, and yellow arrows indicate BMAT. **G** Serum levels of PINP, RANKL, and OPG were measured by ELISA, and the RANKL/OPG ratio was calculated. Data are presented as the mean ± SD. Statistical analyses were performed with the unpaired Student’s *t* test. *P < 0.05 compared with the control mice. ^#^*P* < 0.05 compared with the OVX mice. ns, not statistically significant.

Given that reduced ITGBL1 expression was closely associated with oestrogen deficiency-induced osteoporosis (Fig. 1H, I), we next examined the mRNA levels of *Pparg* and *Runx2* in BMSCs and BMMs from OVX mice. Compared with the sham control group, OVX mice showed significantly lower *Runx2* mRNA levels and higher *Pparg* mRNA levels in BMSCs (Fig. 8A). Interestingly, the mRNA levels of *Pparg* and *Runx2* were increased in BMMs from OVX mice (Fig. 8A). These results suggest that ITGBL1 downregulation in oestrogen deficiency-induced osteoporosis is closely linked to disruption of the osteogenic and adipogenic differentiation balance of BMSCs and enhanced bone resorption mediated by BMMs.

We next tested whether ITGBL1 supplementation could effectively alleviate osteoporosis caused by oestrogen deficiency. Mice with OVX-induced osteoporosis were prophylactically treated by intra-articular injection of AAV9-Itgbl1 into the knee joints, with AAV9-Vector injection serving as a control (Fig. 8B, and Supplementary Fig. 8A). Although the uteri of OVX mice were atrophic compared with those of sham mice and AAV9-Itgbl1 treatment did not improve uterine atrophy (Supplementary Fig. 8B), the reduced bone mass in OVX mice was significantly rescued after AAV9-Itgbl1 treatment (Fig. 8C), as evidenced by the restoration of trabecular BMD, BV/TV, BS/TV, Tb.N, and Tb.Th, the reduction of trabecular Tb.Sp, and the recovery of the cortical bone parameters Cor-BMD and Cb.Th (Fig. 8D). Along with the increase in ITGBL1 expression, the increase in the number of osteoclasts and the accumulation of BMAT in the bone marrow of mice were reduced (Fig. 8E, F). Consistently, the serum RANKL concentration and the RANKL/OPG ratio were decreased, while the PINP and OPG concentrations were increased (Fig. 8G). Notably, AAV9-Itgbl1 treatment did not significantly affect bone mass, osteoclast number, or BMAT accumulation in the bone marrow of sham-treated mice (Fig. 8C–F). Furthermore, ITGBL1 and RUNX2 expression levels were decreased in the bone marrow tissues of OVX mice but were restored after ITGBL1 treatment, whereas PPARγ expression showed the opposite trend, which is consistent with the reciprocal regulation among ITGBL1, PPARγ, and RUNX2 (Supplementary Fig. 8A). Taken together, these results indicate that oestrogen deficiency-induced osteoporosis is associated with ITGBL1 downregulation in BMSCs and BMMs and that ITGBL1 treatment substantially prevents oestrogen deficiency-induced bone loss and alleviates osteoporosis severity.

### ITGBL1 treatment inhibits obesity and osteoporosis caused by ITGBL1 deficiency

**Fig. 9:**
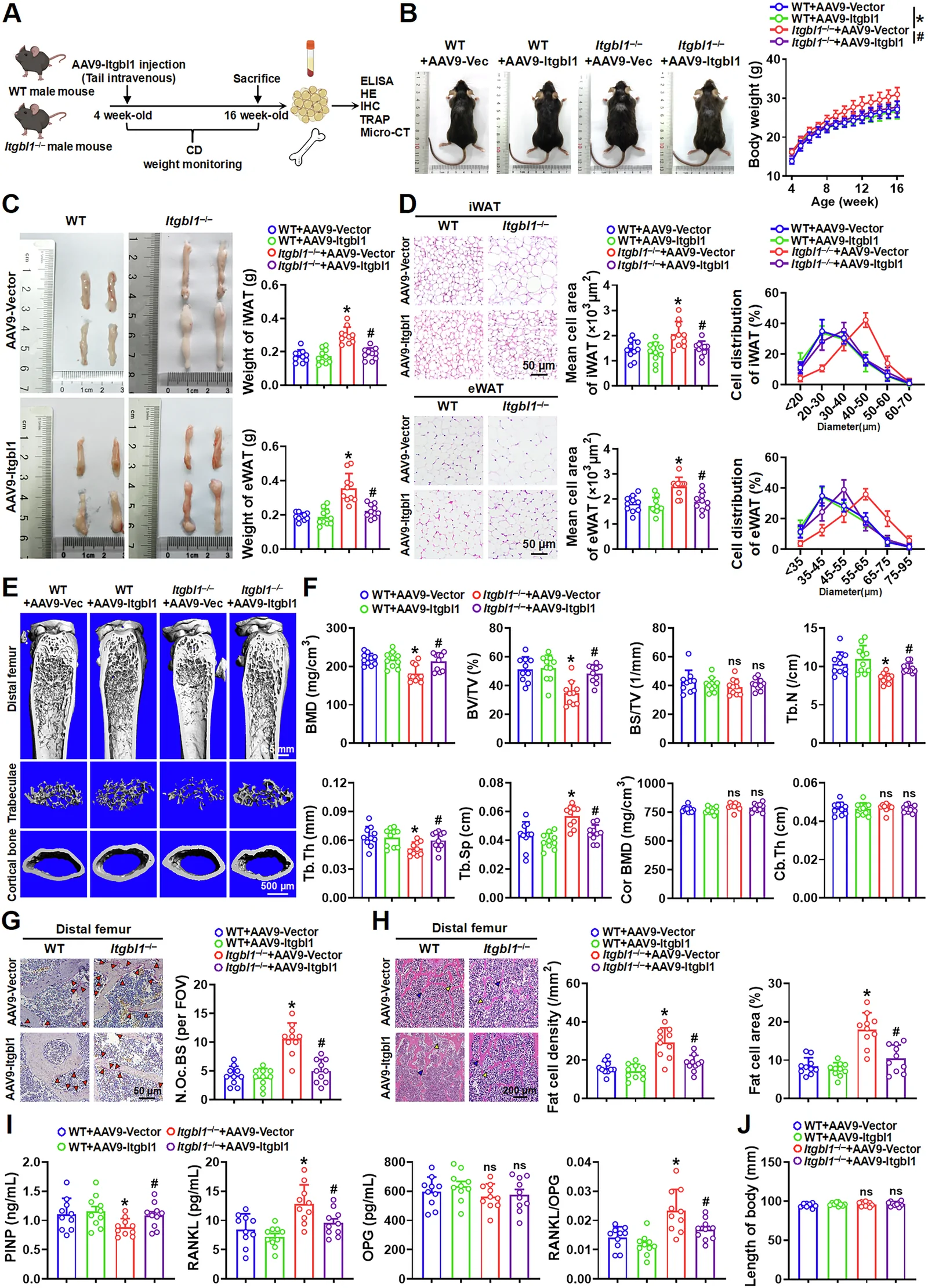
ITGBL1 treatment inhibits obesity and osteoporosis caused by ITGBL1 deficiency. WT and *Itgbl1*^*–/*–^ male C57BL/6 J mice fed a CD were injected with AAV9-Vector or AAV9-Itgbl1 via the tail vein (2 × 10^11^ vg) at 4 weeks of age and allowed to grow to 16 weeks of age (*n*  =  10 per group). **A** Schematic representation of the experimental design of AAV9-mediated ITGBL1 treatment in mice. **B** Representative images and body weight growth curves of the mice. **C** Representative images and weight quantification of iWAT and eWAT collected from the mice. **D** Representative H&E staining images and quantification of the adipocyte area and diameter distribution of iWAT and eWAT. **E** Representative micro-CT images of distal femurs, trabeculae and midshaft cortical bone. **F** Quantification of the BMD, BV/TV, Tb.N, Tb.Th, BS/TV, Tb.Sp, Cor-BMD, and Cb.Th at the distal femur and midshaft cortical bone by micro-CT analysis. **G** Representative TRAP staining images and quantification of osteoclast numbers in the femoral bone marrow. **H** Representative images and quantification of the number and area of adipocytes in the distal femoral bone marrow. Blue arrows indicate trabecular bone, and yellow arrows indicate BMAT. **I** Serum levels of PINP, RANKL, and OPG were measured by ELISA, and the RANKL/OPG ratio was calculated. **J** Body length of the mice in each group. The data are shown as the mean ± SD. Statistical analyses were performed with the unpaired Student’s t test or two-way ANOVA (**B**). *P < 0.05 compared with the control mice. ^#^*P* < 0.05 compared with *Itgbl1*^*–/–*^ mice. ns, not statistically significant.

Since ITGBL1 supplementation can alleviate diet-induced and oestrogen deficiency-induced obesity and/or osteoporosis, we sought to determine whether systemic ITGBL1 supplementation could reduce body weight and increase bone mass in *Itgbl1*^*–/–*^ mice. To test this hypothesis, 4-week-old WT and *Itgbl1*^*–/–*^ mice were treated with AAV9 carrying *Itgbl1* or vector control via tail vein injection and were fed a standard rodent CD until 16 weeks of age (Fig. 9A, and Supplementary Fig. 9A, B). As expected, AAV9-Itgbl1 treatment slowed weight gain in *Itgbl1*^*–/–*^ mice and even restored their body weight to a level comparable to that of control mice (Fig. 9B). Histological analysis revealed that *Itgbl1*^*–/–*^ mice treated with AAV9-Itgbl1 had lower iWAT and eWAT weights (Fig. 9C) and smaller unilocular lipid droplets in WAT (Fig. 9D). Correspondingly, the reduction in femoral bone mass in *Itgbl1*^*–/–*^ mice was largely reversed by AAV9-Itgbl1 treatment, with increases in the BMD, BV/TV, Tb.N, and Tb.Th and a decrease in the Tb.Sp of trabecular bone (Fig. 9E, F). Histological analysis further revealed that the increased osteoclast numbers and BMAT accumulation in the primary and secondary ossification centres of *Itgbl1*^–/–^ mice were significantly reduced following AAV9-Itgbl1 treatment (Fig. 9G, H). In addition, after AAV9-Itgbl1 treatment, the serum RANKL concentration and the RANKL/OPG ratio were decreased and the PINP concentration was increased (Fig. 9I). Importantly, AAV9-Itgbl1 treatment did not noticeably affect the above phenotypes in WT mice or the body length of either WT or *Itgbl1*^*–/–*^ mice (Fig. 9J). Additionally, compared with that in WT mice, PPARγ protein expression was increased in iWAT and bone marrow tissue, while RUNX2 protein expression was decreased in bone marrow tissue but not in iWAT in *Itgbl1*^*–/–*^ mice. Consistently, ITGBL1 treatment restored PPARγ and RUNX2 expression in these tissues (Supplementary Fig. 9A, B). Taken together, these results suggest that ITGBL1 has therapeutic effects on obesity and osteoporosis caused by ITGBL1 deficiency.

## Discussion

ITGBL1, also referred to as osteoblast-specific cysteine-rich protein (OSCP) or ten integrin EGF-like repeat domain-containing protein (TIED), shares structural similarity with the stalk-like region of integrin β-subunits [33]. Our group previously reported that ITGBL1 positively regulates the expression of bone-related genes and that RUNX2 activates *ITGBL1* transcription by directly binding to its proximal promoter region in breast cancer cells [34]. The coexpression of ITGBL1 with RUNX2 and the transcriptional activation of *ITGBL1* by RUNX2 have been verified in various cellular contexts [35–38]. In this study, we reveal that ITGBL1 is a key regulator of the adipogenic and osteogenic lineage commitment of MSCs, and is markedly downregulated under obesity- and osteoporosis-related conditions. ITGBL1 deficiency promotes the adipogenic differentiation of BMSCs, ADMSCs, and adipogenic precursor cells while inhibiting the osteogenic differentiation of BMSCs and osteogenic precursor cells, leading to systemic lipid accumulation and bone loss. Mechanistically, ITGBL1 deficiency biases MSC lineage commitment towards adipogenic differentiation by upregulating PPARγ expression and downregulating RUNX2 expression.

Accumulating evidence has shown that disrupting the balance between the adipogenic and osteogenic differentiation of MSCs is responsible for the epidemiological and physiological associations between obesity and osteoporosis. Fat infiltration in the bone marrow further aggravates low bone mass [39, 40]. Multiple members of the integrin family are involved in regulating the balance between osteogenic and adipogenic differentiation, as well as bone homoeostasis. ITGB1 promotes osteogenesis and inhibits adipogenesis through activating the FAK–AKT signalling pathway [41]. ITGB1 also drives osteogenesis via activation of the cAMP‒PKA signalling pathway [42]. High-integrin αV human MSCs have greater osteogenic potential [43], and αV/β5 integrin mediates skeletal remodelling by acting on osteocytes and adipocytes, thereby contributing to bone loss [44]. Integrin αv/β1 plays a critical role in MSC lineage determination through the promotion of osteogenic differentiation and inhibition of adipogenic differentiation [45]. In this study, we demonstrate that the functions of ITGBL1 in promoting osteogenic differentiation and inhibiting adipogenic differentiation resemble those of several integrin family members. Since ITGBL1 lacks both a transmembrane domain and an RGD (Arg-Gly-Asp)-binding domain for extracellular matrix ligands [33], its functions may involve molecular mechanisms distinct from those of integrin proteins.

PPARγ is a core transcriptional regulator that directs the commitment of MSCs towards the adipocyte lineage. Elevated PPARγ protein levels have been linked to obesity [46–48]. PPARγ also inhibits osteoblast differentiation [20] and promotes osteoclast differentiation and bone resorption [49], leading to osteoporosis [50]. Therefore, PPARγ is a key regulator of obesity and osteoporosis, two tightly linked diseases. The identification of the upstream modulators that control PPARγ levels is crucial for the prevention and treatment of these conditions. E3 ubiquitin ligase-mediated ubiquitination and proteasomal degradation is a common mechanism underlying the posttranslational regulation of PPARγ. MKRN1 [51], CRL4B [52], and NEDD4 [32, 53, 54] have been identified as mediators of PPARγ ubiquitination and proteasomal degradation. Here, we report that ITGBL1 reduces PPARγ protein levels by assembling NEDD4 to target PPARγ for ubiquitination and proteasomal degradation. Thus, the loss of ITGBL1 stabilizes PPARγ by impairing NEDD4-mediated degradation.

RUNX2 is a core transcriptional regulator that determines the commitment of MSCs to the osteoblast lineage, directing them to differentiate into preosteoblasts while inhibiting their adipogenic and chondrogenic differentiation [19]. Reciprocal repression between PPARγ and RUNX2 orchestrates the osteogenic–adipogenic differentiation equilibrium in MSCs [15]. Mutual transcriptional repression has also been observed in COS7 cells [55]. In this study, we revealed that reciprocal transrepression occurred between PPARγ and RUNX2 in a specific cellular context. RUNX2 transrepressed *Pparg* in both osteogenic and adipogenic lineage precursor cells, whereas PPARγ transrepressed *Runx2* only in osteogenic lineage precursor cells and not in adipose tissue-derived adipogenic precursor cells. RNA-seq data also revealed that the ITGBL1 deficiency-induced upregulation of PPARγ did not lead to RUNX2 downregulation in ADMSCs. A possible explanation for these findings is that adipose tissue-derived MSCs or precursor cells are not responsible for bone formation in vivo, thus, PPARγ does not regulate the transcription of RUNX2, although ADMSCs, which possess multilineage differentiation potential, are commonly used for bone tissue repair and regeneration in vitro [56, 57]. Additionally, PPARγ and RUNX2 have opposite effects on the transcription of *Itgbl1* in both osteogenic and adipogenic lineage precursor cells. Therefore, ITGBL1, PPARγ, and RUNX2 constitute an interconnected regulatory network‌ that governs the lineage commitment of MSCs.

Osteoporosis is caused by the dysregulation of bone homoeostasis that depends on the precise regulation of the equilibrium between bone formation and resorption [58]. Osteogenesis and osteoclastogenesis, both of which are involved in bone remodelling, act together to regulate bone homoeostasis [59]. The disruption of bone remodelling decreases osteogenesis and increases osteoclastogenesis, driving the pathological destruction of bone microarchitecture in osteoporosis [60]. In addition to the inherent decrease in bone formation during the adipogenic differentiation of BMSCs, BMAT further stimulates osteoclast differentiation [23–25]. Bone marrow adipogenic lineage precursor cells have also been shown to promote osteoclast differentiation and bone resorption [21, 22]. In our study, we revealed that the loss of ITGBL1 not only plays crucial roles in promoting the adipogenic differentiation and inhibiting the osteogenic differentiation of BMSCs but also promotes osteoclast differentiation by inducing the production of osteoclastogenesis-inducing factors. Therefore, ITGBL1 deficiency leads to osteoporosis not only by reducing osteogenesis but also by increasing BMAT mass, thereby promoting osteoclast differentiation and bone resorption.

It has been reported that ITGBL1 is markedly reduced in the damaged articular cartilage of patients with osteoarthritis, loss of ITGBL1 leads to cartilage damage, and overexpression of ITGBL1 prevents knee joint degeneration in a mouse model of surgically induced osteoarthritis [61]. In our study, we reveal that *Itgbl1* expression is significantly decreased in ADMSCs, BMSCs, iWAT, and eWAT from DIO mice, as well as in BMSCs from OVX mice. Our results indicate that in addition to hereditary deficiency of ITGBL1 leading to obesity and osteoporosis, lipid uptake and oestrogen deficiency significantly downregulate ITGBL1 expression, which in turn cause obesity and osteoporosis. These findings demonstrate that ITGBL1 is critical for maintaining the balance between the osteogenic and adipogenic differentiation of MSCs and has notable medicinal effects for the prevention and treatment of obesity and osteoporosis.

Existing treatments for obesity include lifestyle changes (such as diet and exercise), liposuction surgery, and medications. However, the compliance of patients with lifestyle changes is often poor, and liposuction surgery is associated with high risks [62]. Clinically, only a limited number of drugs are available, and most of these small-molecule compounds act by reducing appetite, increasing satiety, or slowing gastric emptying, but they are associated with significant side effects [63]. Current treatments for osteoporosis primarily involve medications such as bisphosphonates and calcitonin, which inhibit osteoclast activity, and teriparatide, which stimulates osteoblast activity and bone formation [64]. However, these drugs can also cause adverse effects such as hypocalcaemia and osteonecrosis. In recent years, the humanized anti-RANKL monoclonal antibody denosumab has been approved for clinical use. However, this treatment is reversible, with a rapid increase in bone turnover and subsequent bone loss after drug discontinuation [65]. To date, there is a lack of safe and effective drugs that jointly target the aetiology and pathogenesis of both obesity and osteoporosis.

AAV9-mediated gene delivery has been widely applied in the clinical treatment of multiple diseases [66, 67]. AAV9 has been employed as a vehicle for one-time administration, and its safety and efficacy have been demonstrated in clinical trials [68, 69]. In this study, we generated an AAV9 delivery system for *Itgbl1* gene therapy to protect against obesity and osteoporosis in mice with systemic ITGBL1 deficiency and in DIO mice, and to attenuate bone loss in OVX mice. Neither the preventive administration of AAV9-Itgbl1 via the tail vein nor the knee joint cavity was associated with observable side effects, including effects on body development or the structure and function of the liver and kidney. Therefore, our findings reveal a novel aetiology and pathogenesis of obesity and osteoporosis and shed light on the development of prevention and treatment strategies for these tightly linked public health concerns.

Several limitations of this study should be acknowledged. Although our ex vivo experiments in primary ADMSCs/BMSCs and precursor cell lines support the key role of ITGBL1 in regulating mesenchymal lineage commitment and show that AAV9-mediated ITGBL1 supplementation significantly alleviates obesity and/or osteoporosis in HFD-fed mice, OVX mice and systemic ITGBL1-deficient mice, future studies using MSC-specific *Itgbl1* knockout models will be required to definitively establish the MSC-autonomous role of ITGBL1. Moreover, AAV9 can mediate transgene expression in liver and brain tissues after systemic administration, which is consistent with the known biology of systemic AAV9 delivery [28, 70]. Although the results of this study support the therapeutic potential of ITGBL1 supplementation, future studies using dose-escalation and tissue-specific promoters will be necessary before translational conclusions can be drawn.

In conclusion, we have demonstrated that ITGBL1 is a key regulator of MSC lineage differentiation, maintaining a balance between osteogenic and adipogenic differentiation to coordinate systemic skeletal and lipid homoeostasis. Its mechanism involves regulating NEDD4-mediated PPARγ ubiquitination and degradation and forming an environment-dependent repressive loop with RUNX2, thereby guiding MSC differentiation. More importantly, our results indicate that ITGBL1 has significant medicinal effects for the simultaneous prevention and treatment of obesity and osteoporosis. The development of ITGBL1 or its agonists for the combined treatment of obesity and osteoporosis would be an exciting and worthwhile research direction.

## Supplementary information

Supplementary Materials

Western source data

## Data Availability

All the data in this study are available from the corresponding author upon reasonable request. All the data generated or analysed in this study are included within the article and its Supplemental material.
