# Supplementary material for: ITGBL1 inhibits obesity and osteoporosis by maintaining adipogenic and osteogenic differentiation balance via the NEDD4–PPARγ–RUNX2 axis
Source: Cell Death Dis. 2026 Sep 2;17(1):926. doi: 10.1038/s41419-026-09220-z (PMC13722705; doi:10.1038/s41419-026-09220-z)

# Source data of Fig. 3

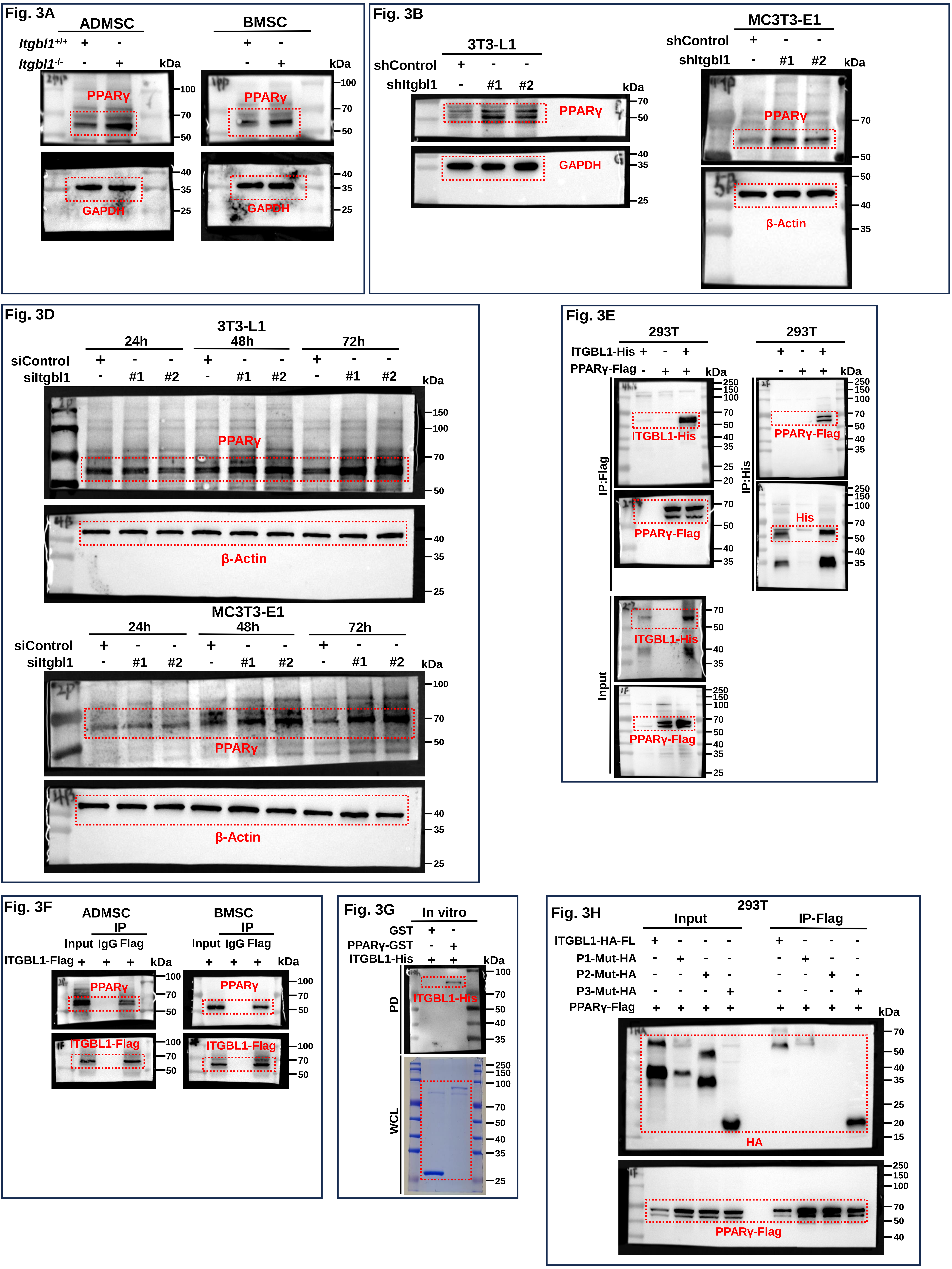

# Source data of Fig. 3

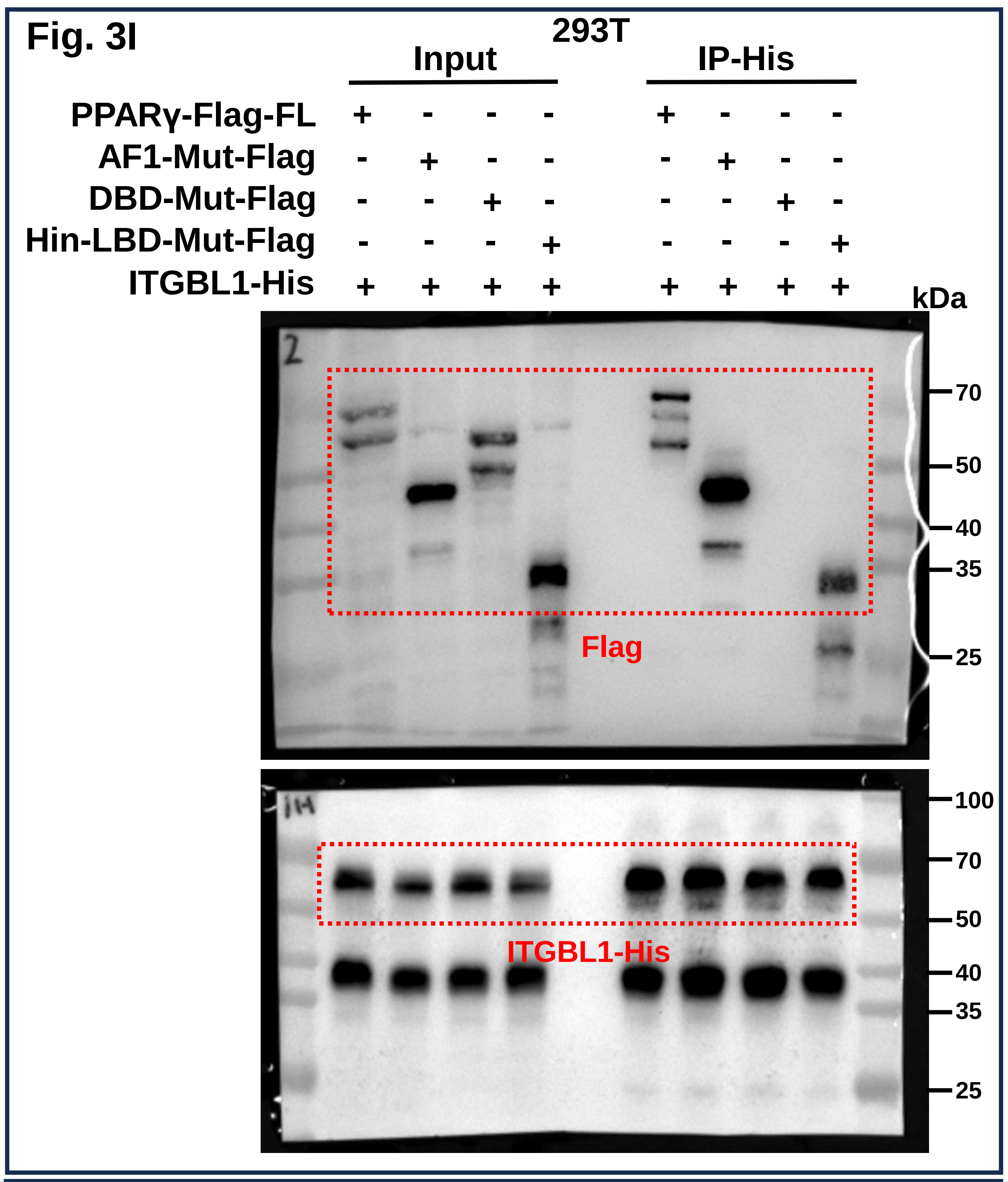


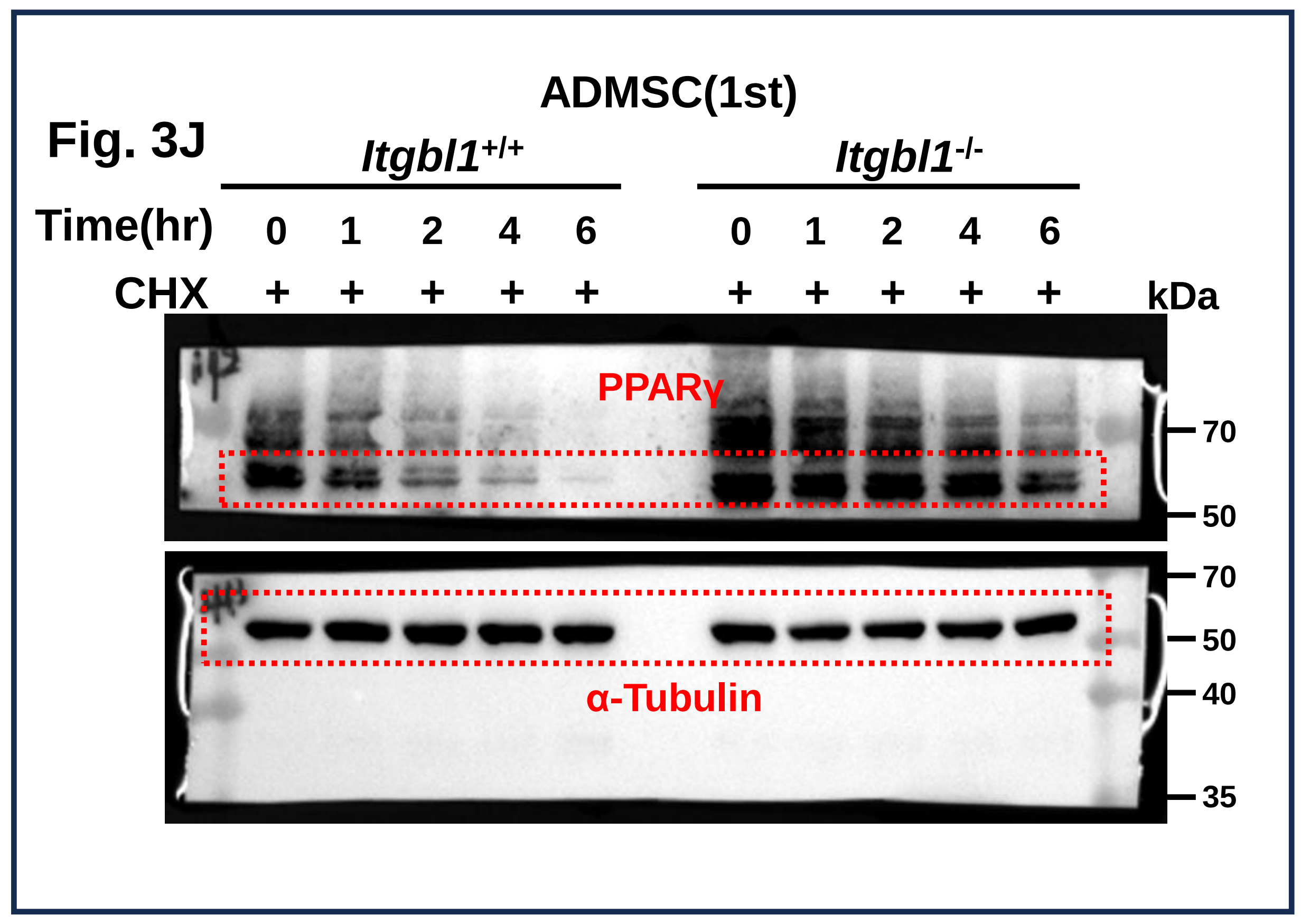


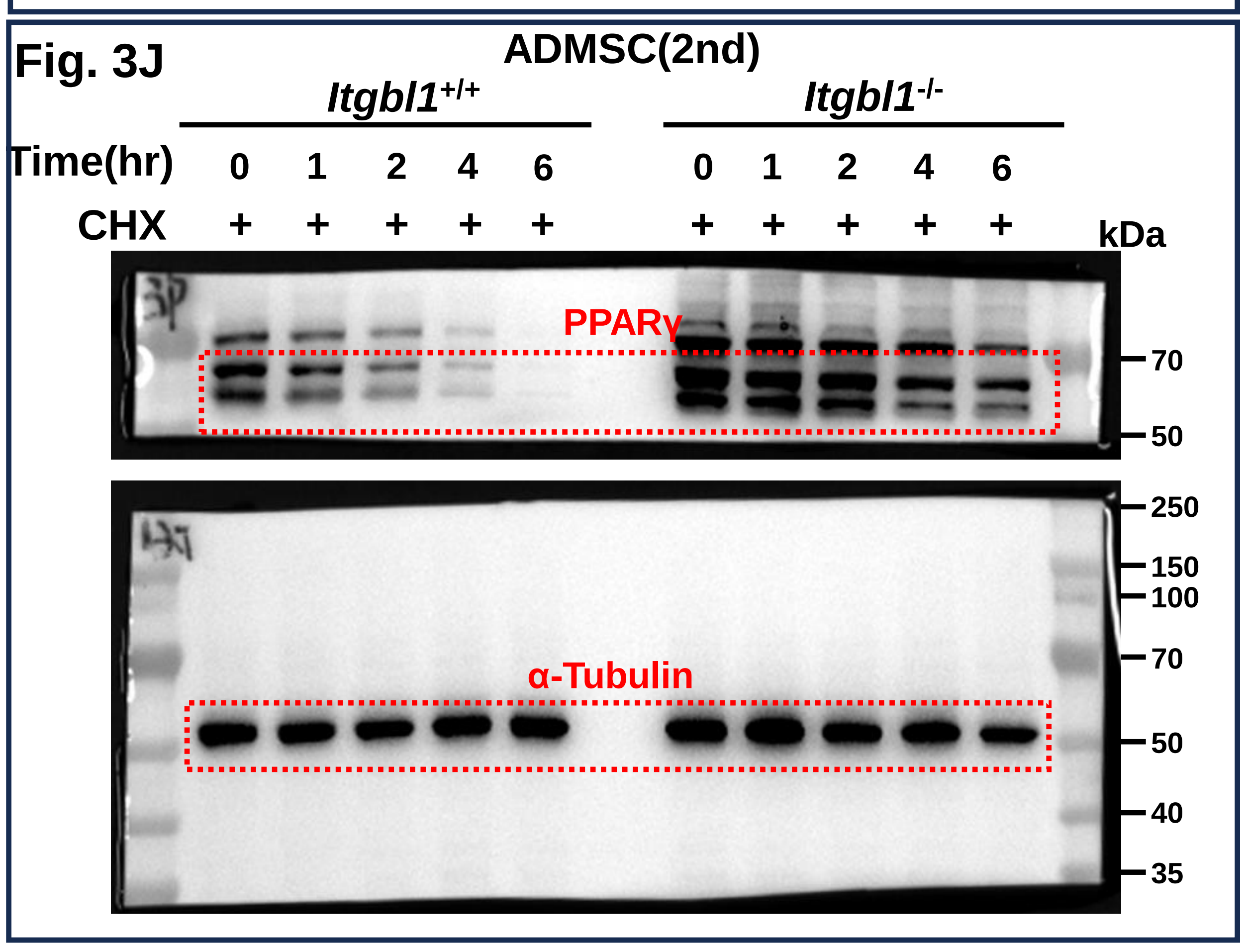


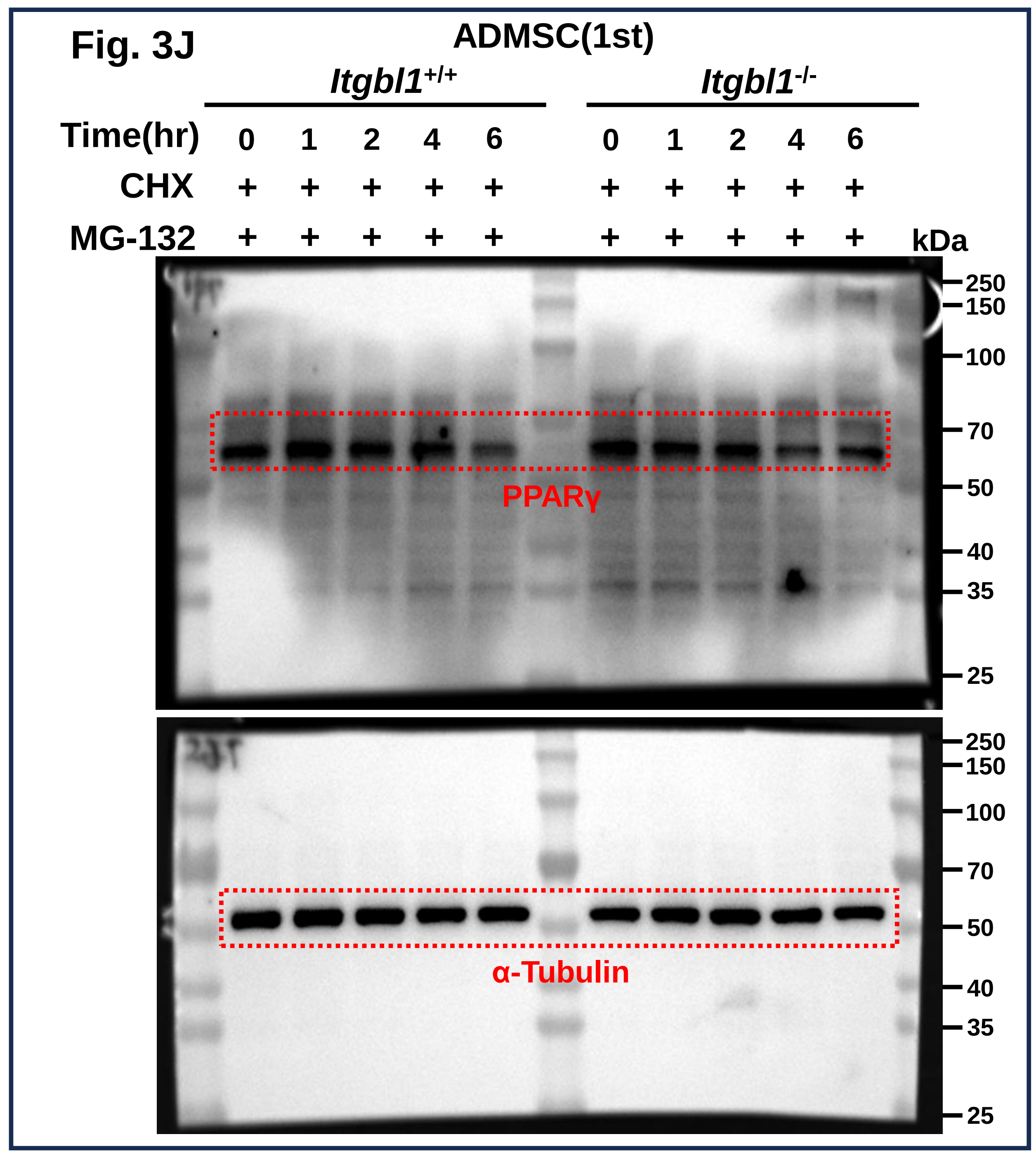


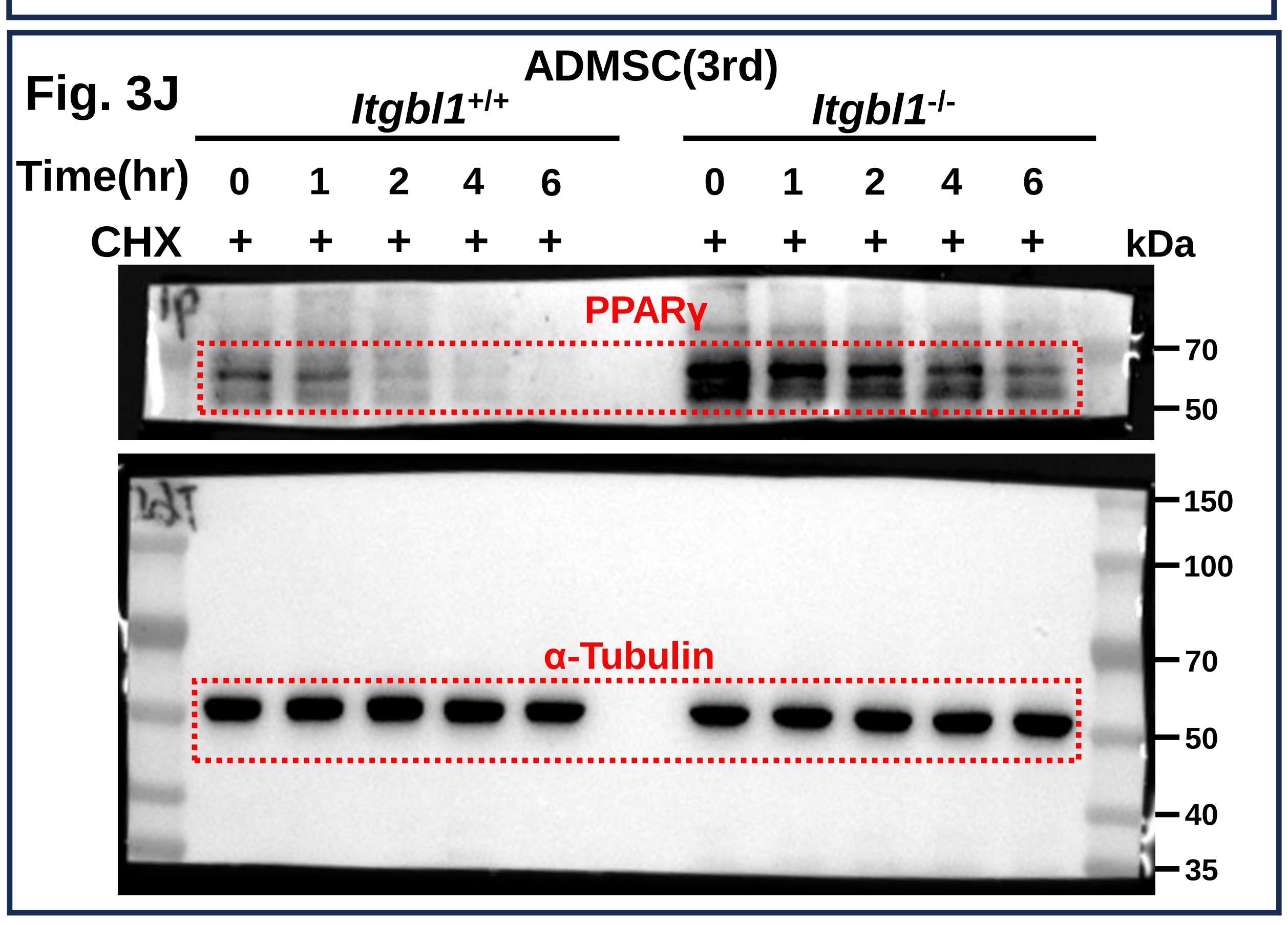


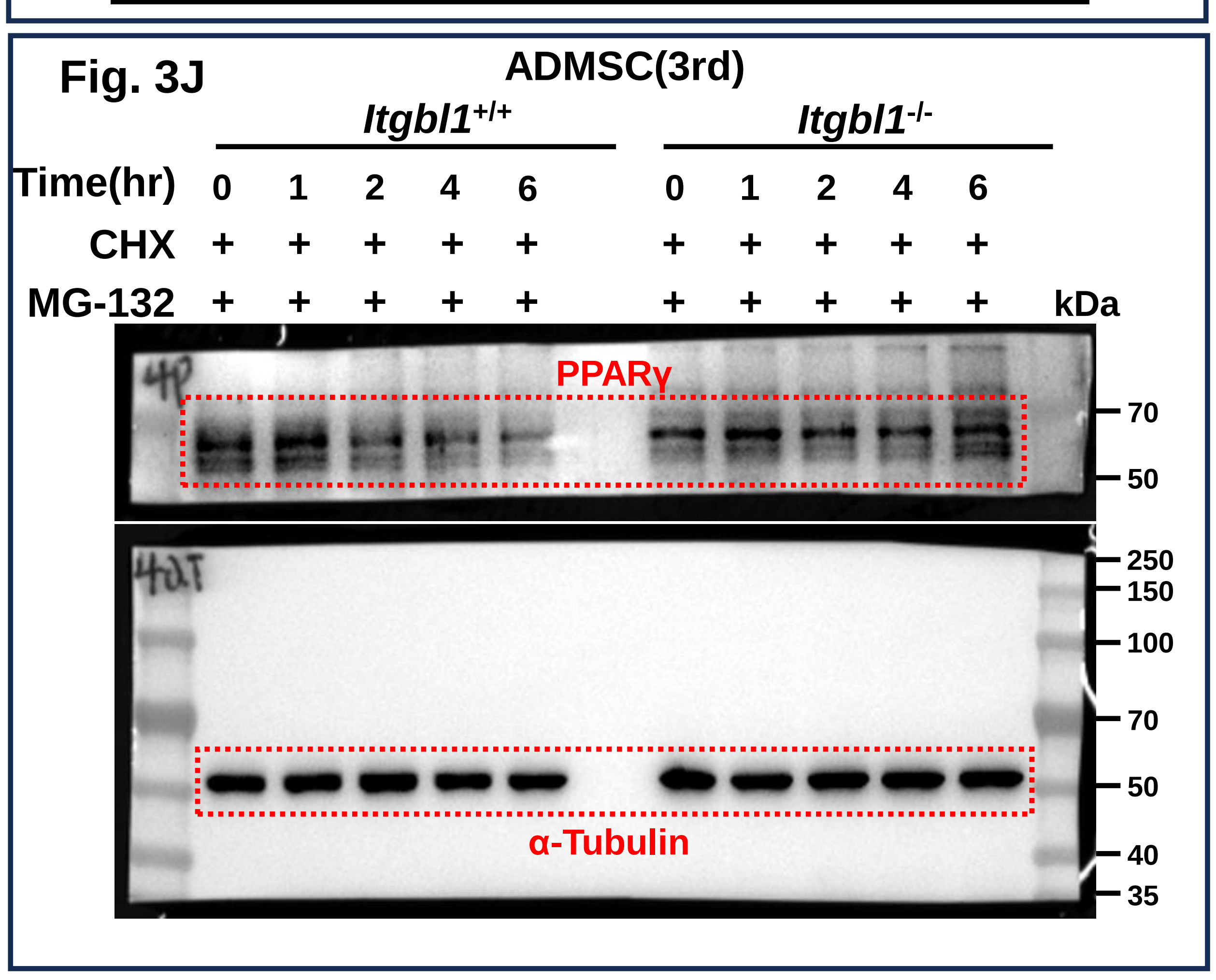


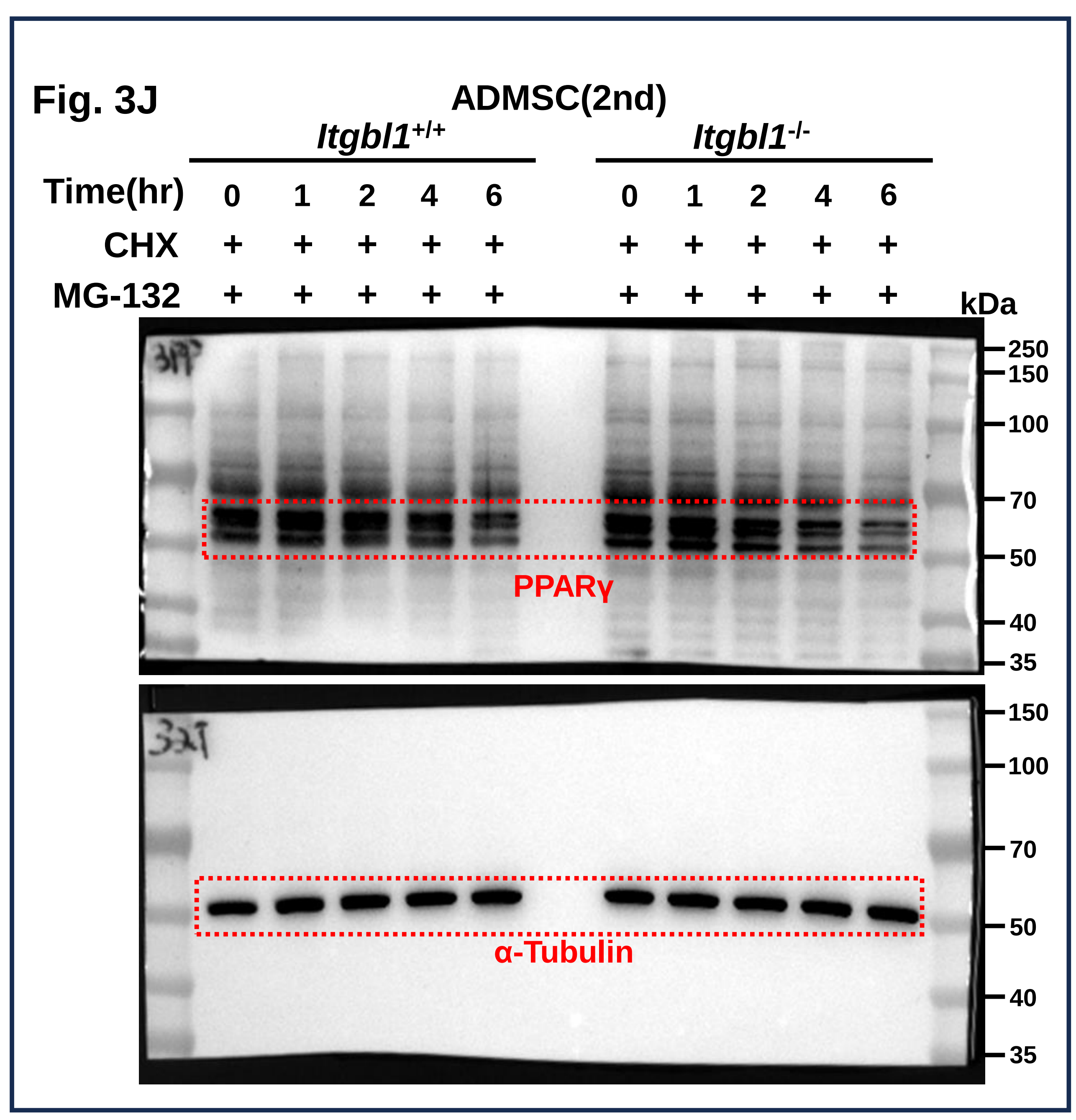

# Source data of Fig. 3

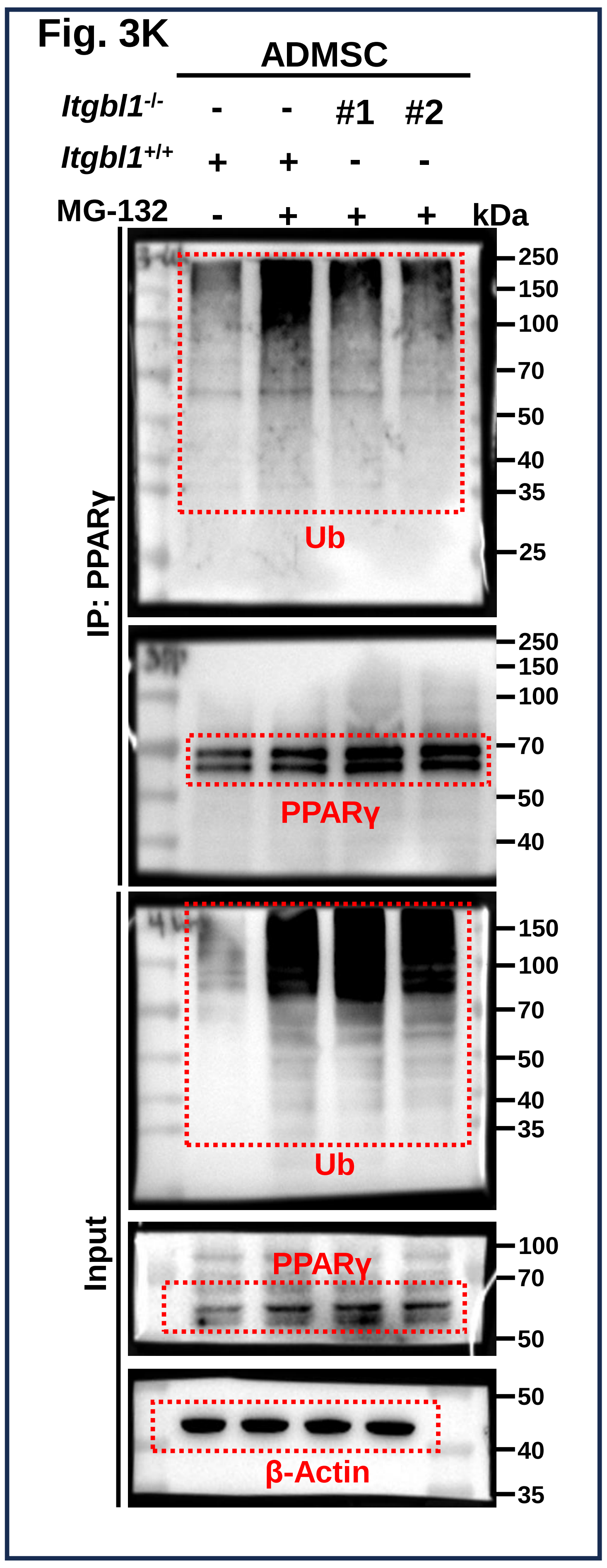


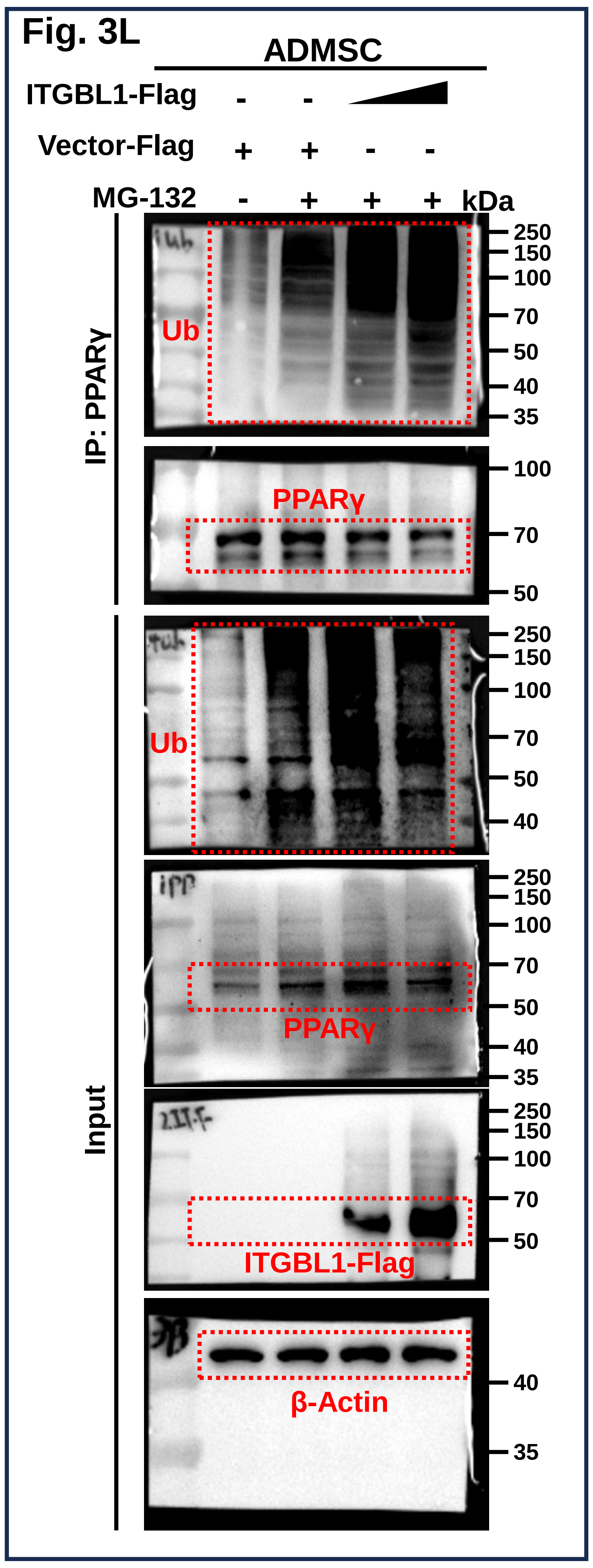

# Source data of Fig. S3

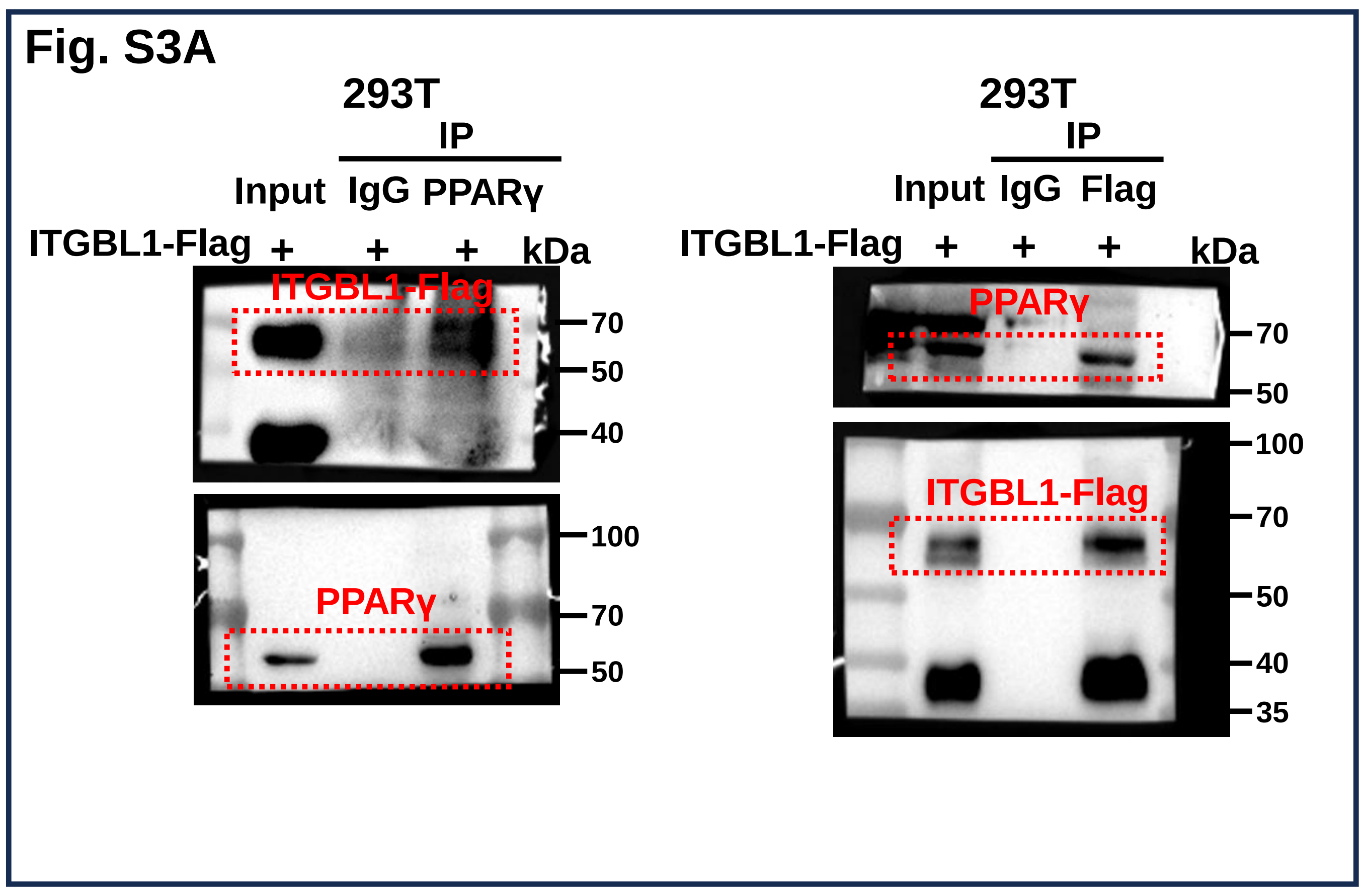


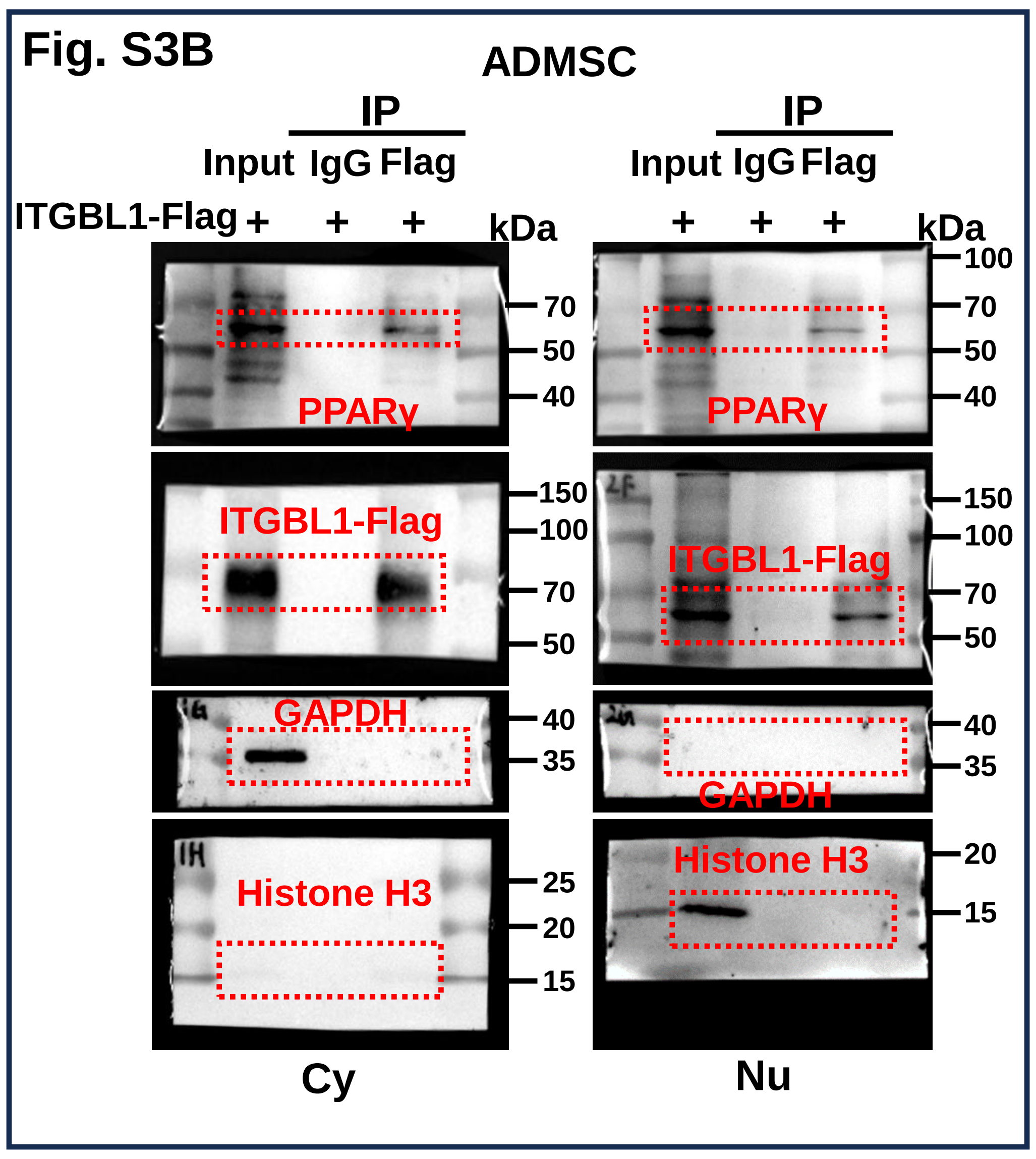


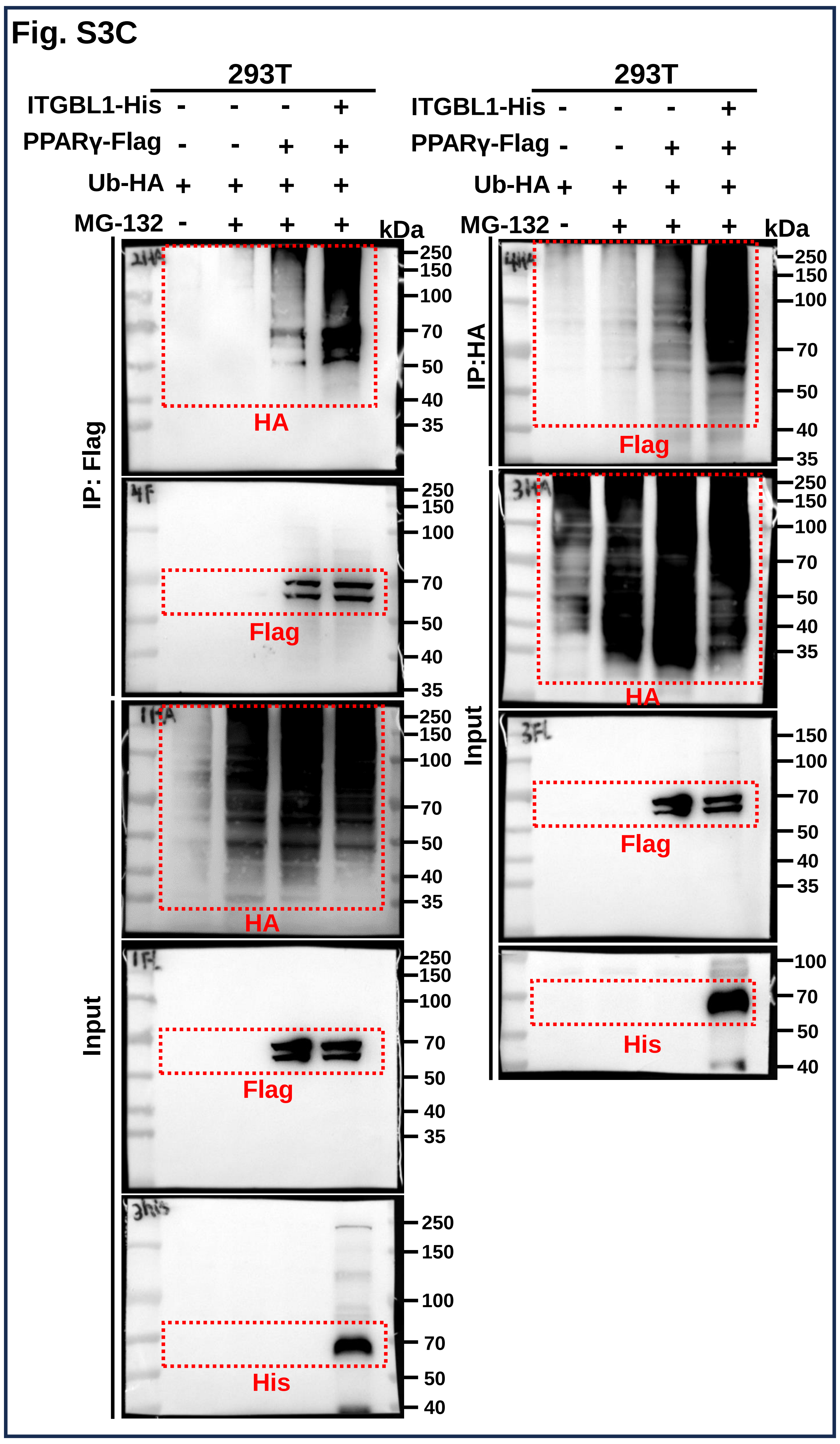


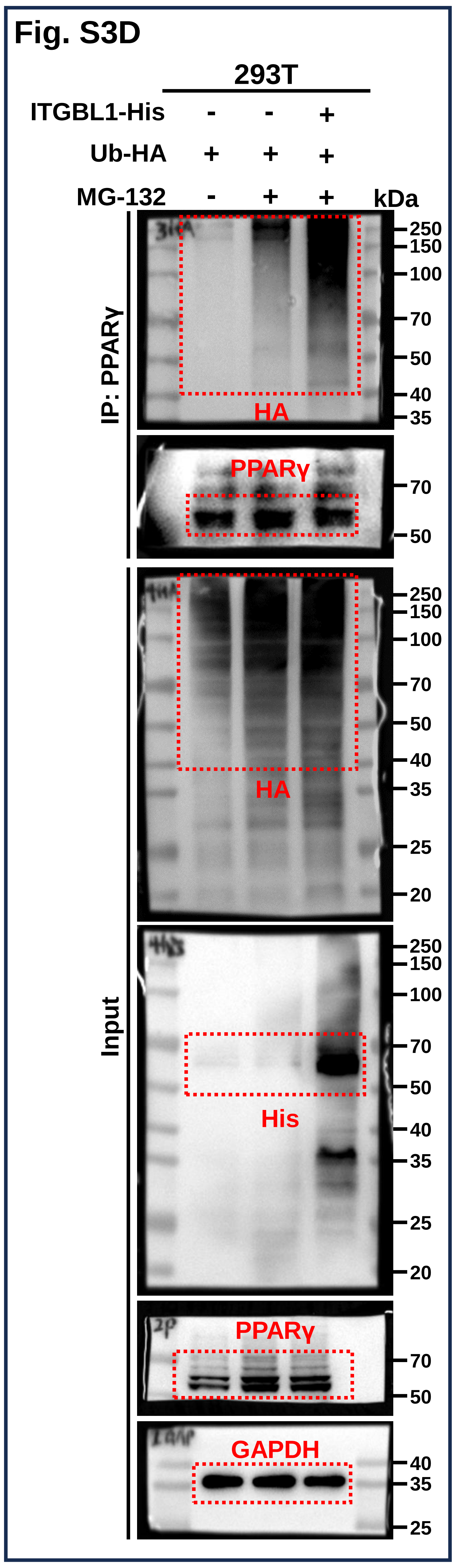

# Source data of Fig. 4

Fig. 4B

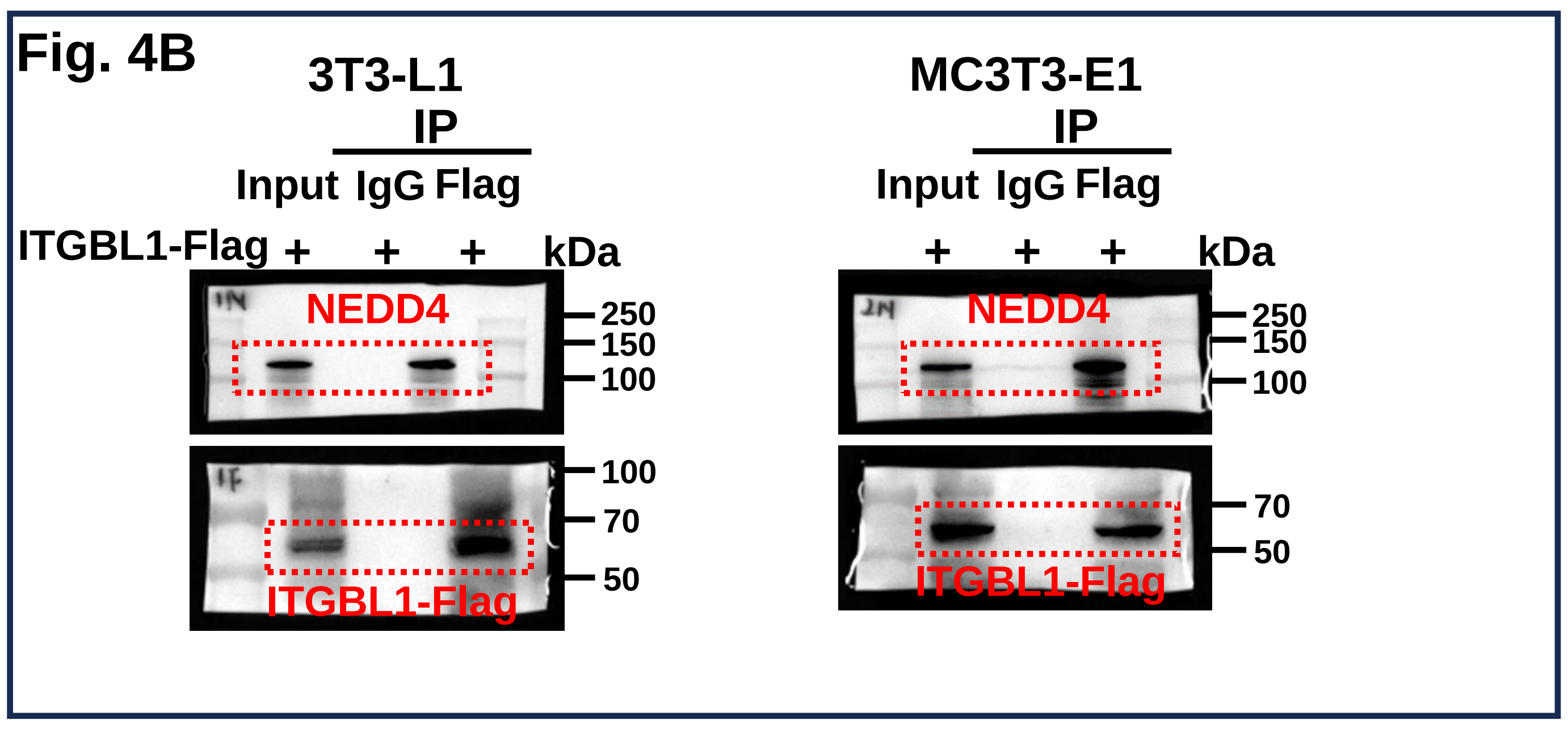


Fig. 4C

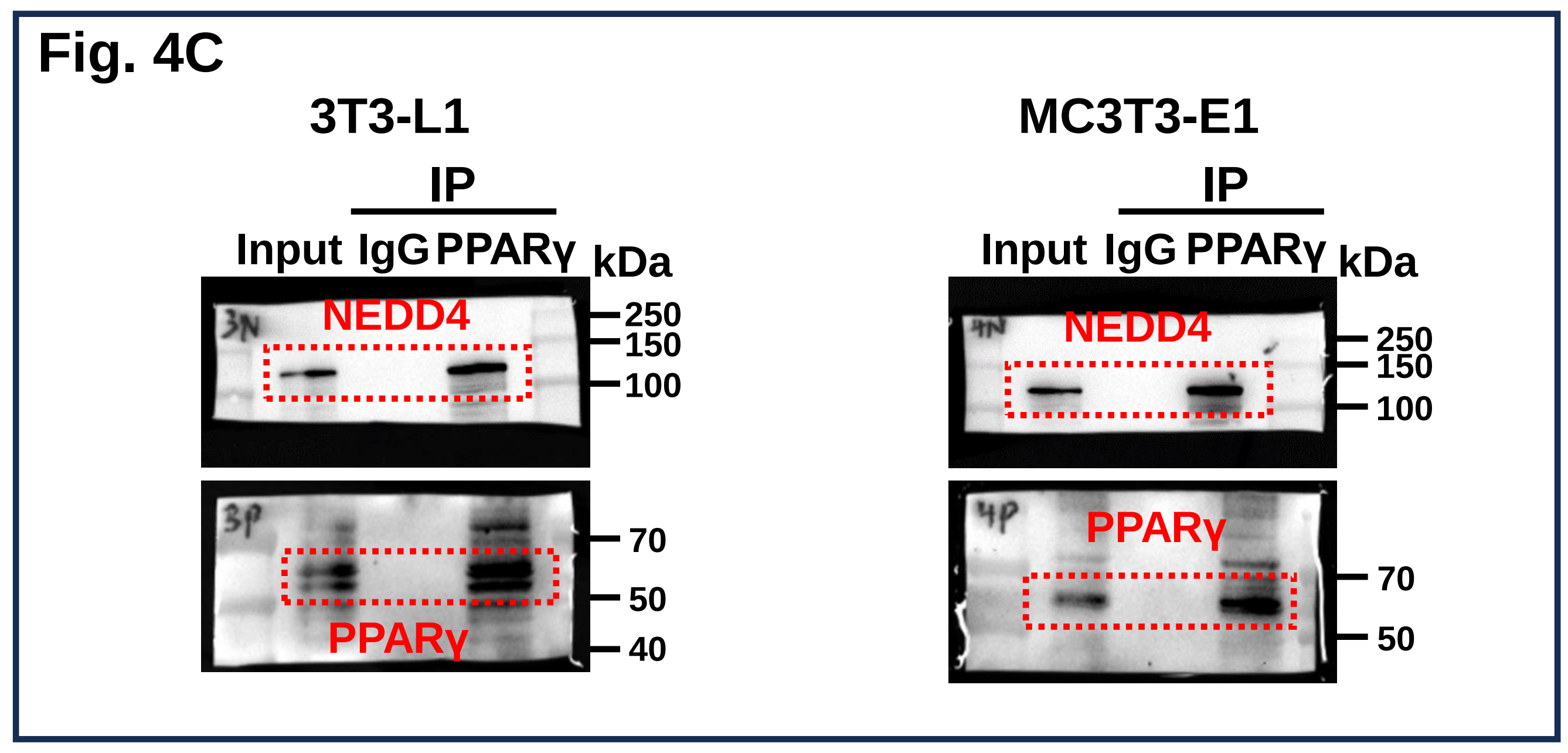


Fig. 4D

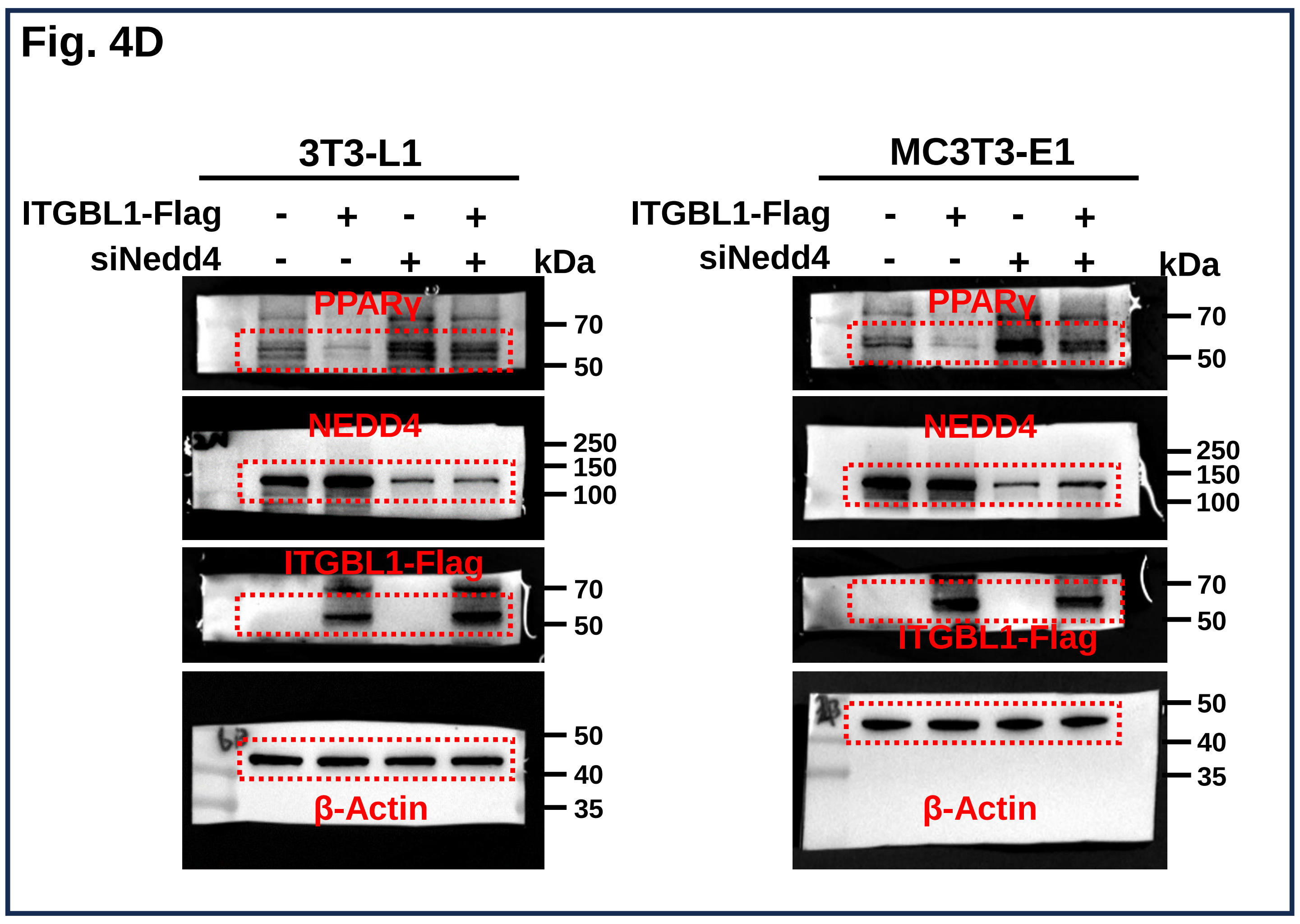


Fig. 4E

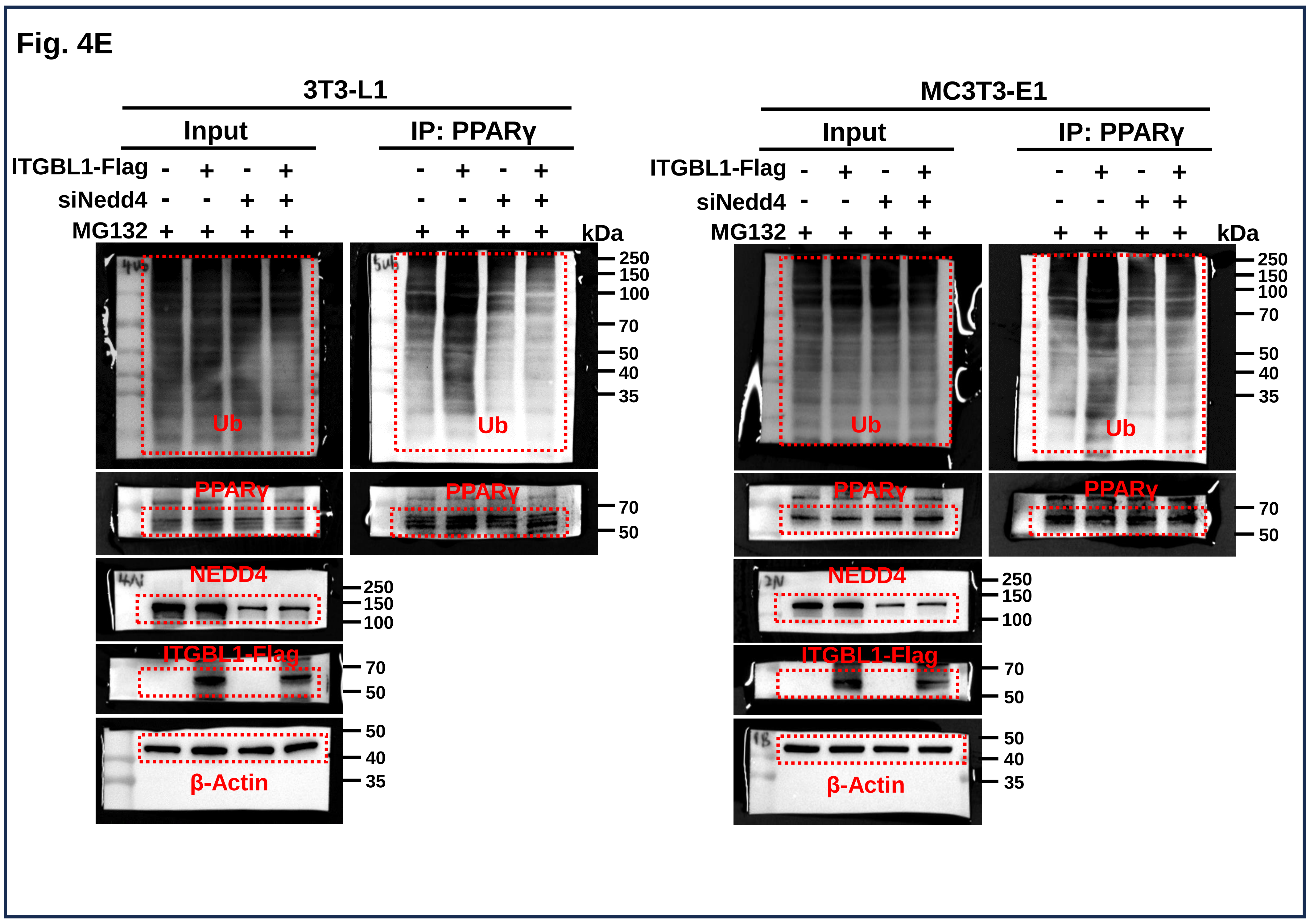


Fig. 4F

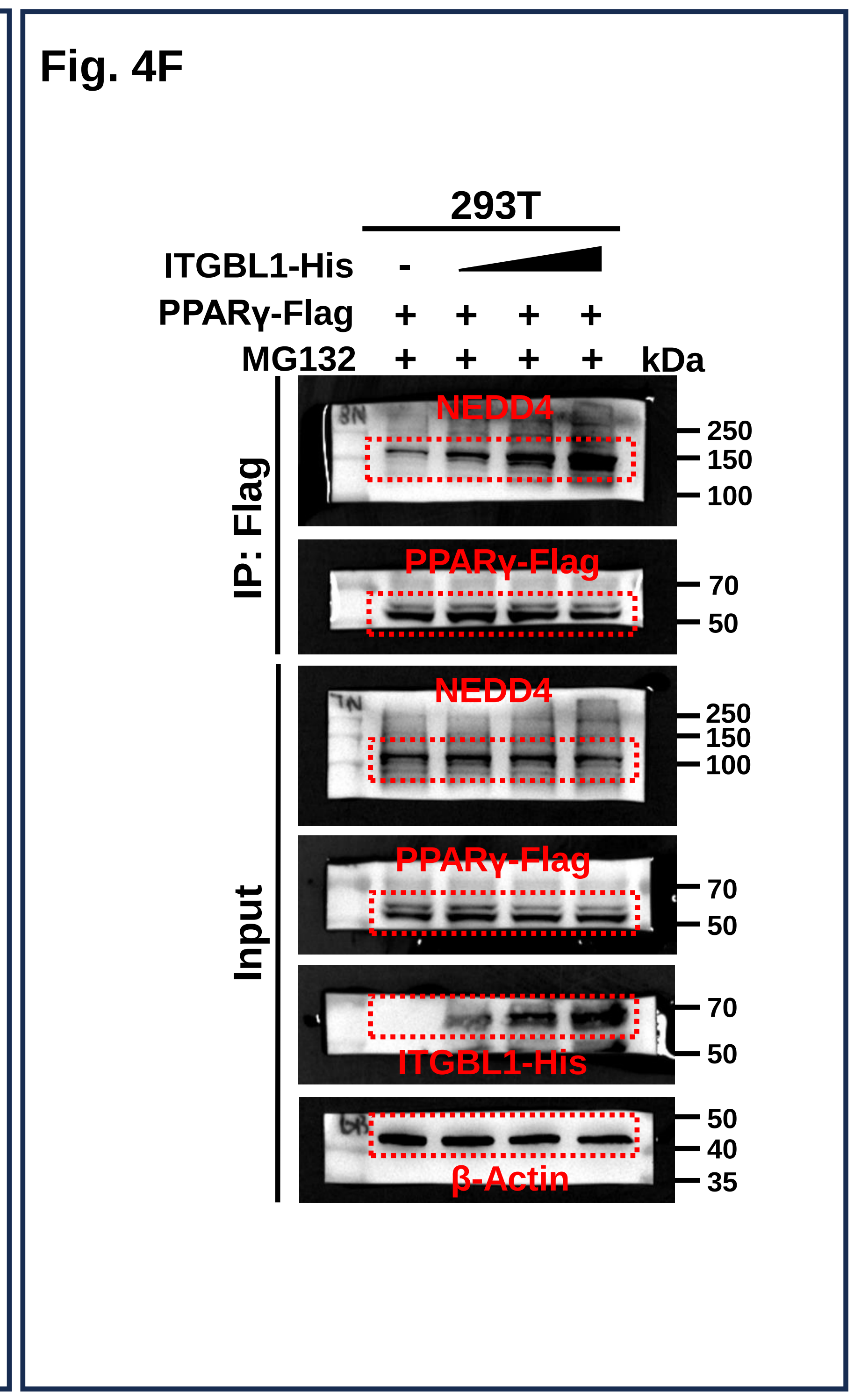

# Source data of Fig. S4

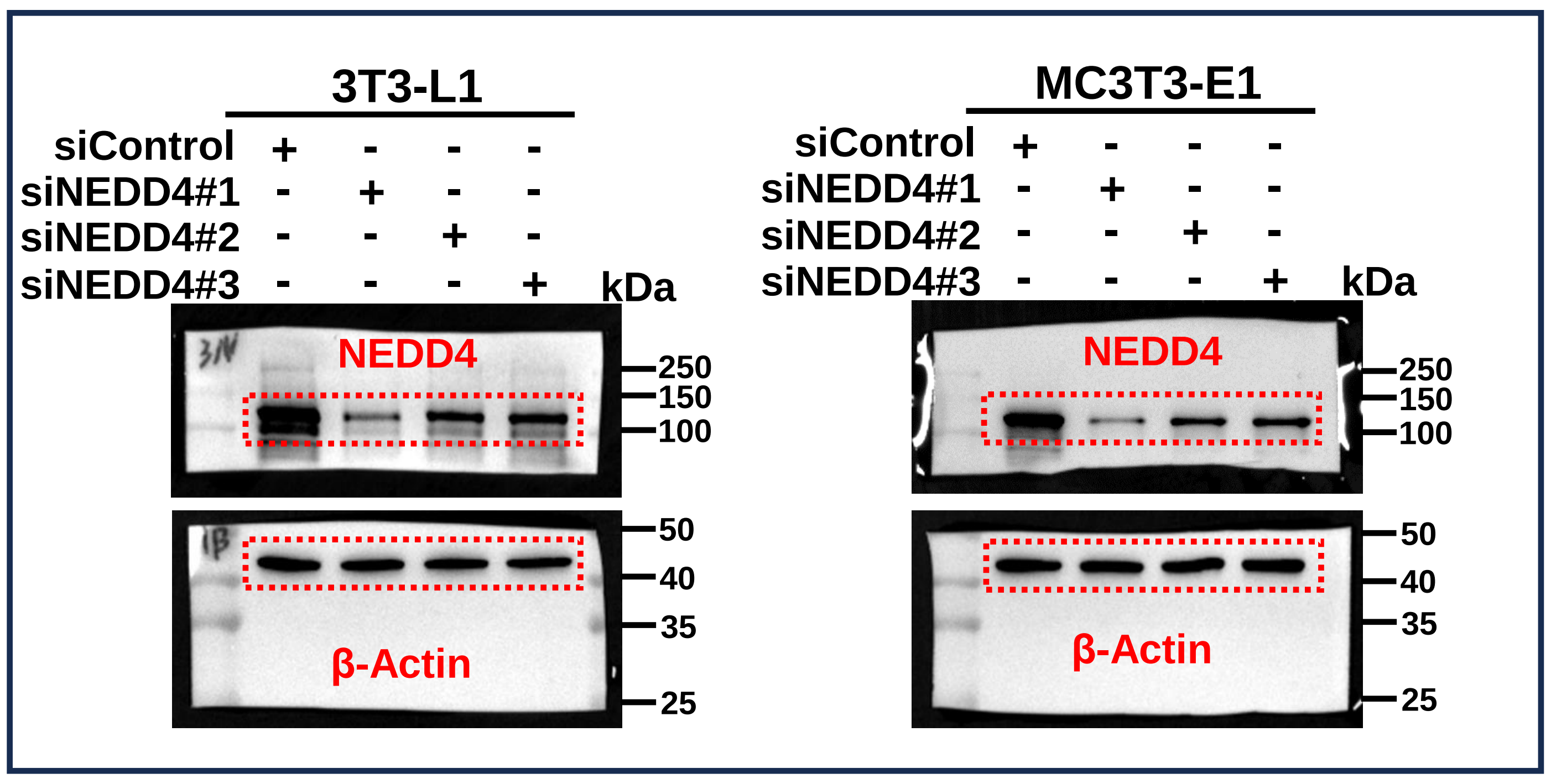

# Source data of Fig. 5

Fig. 5A

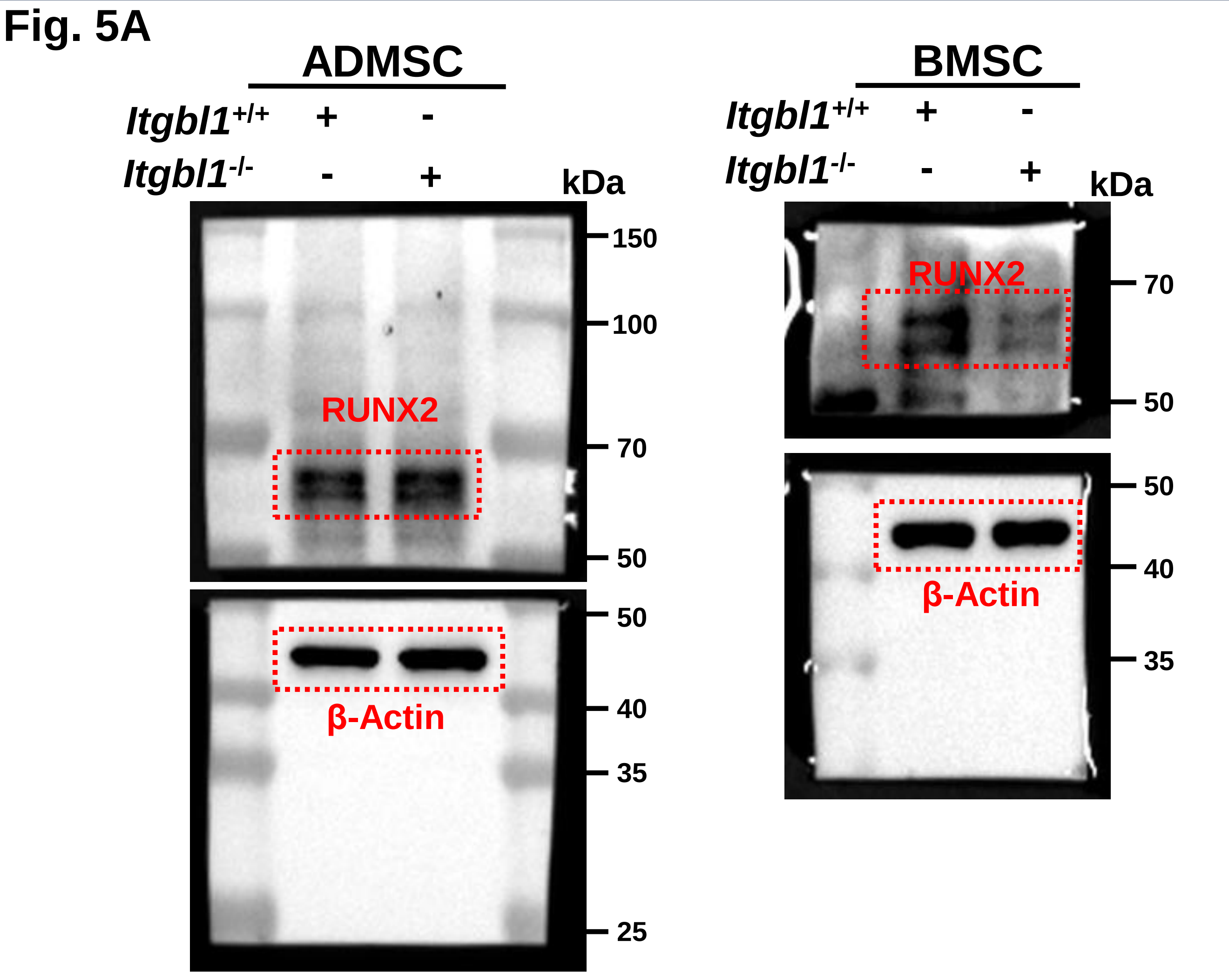


Fig. 5B

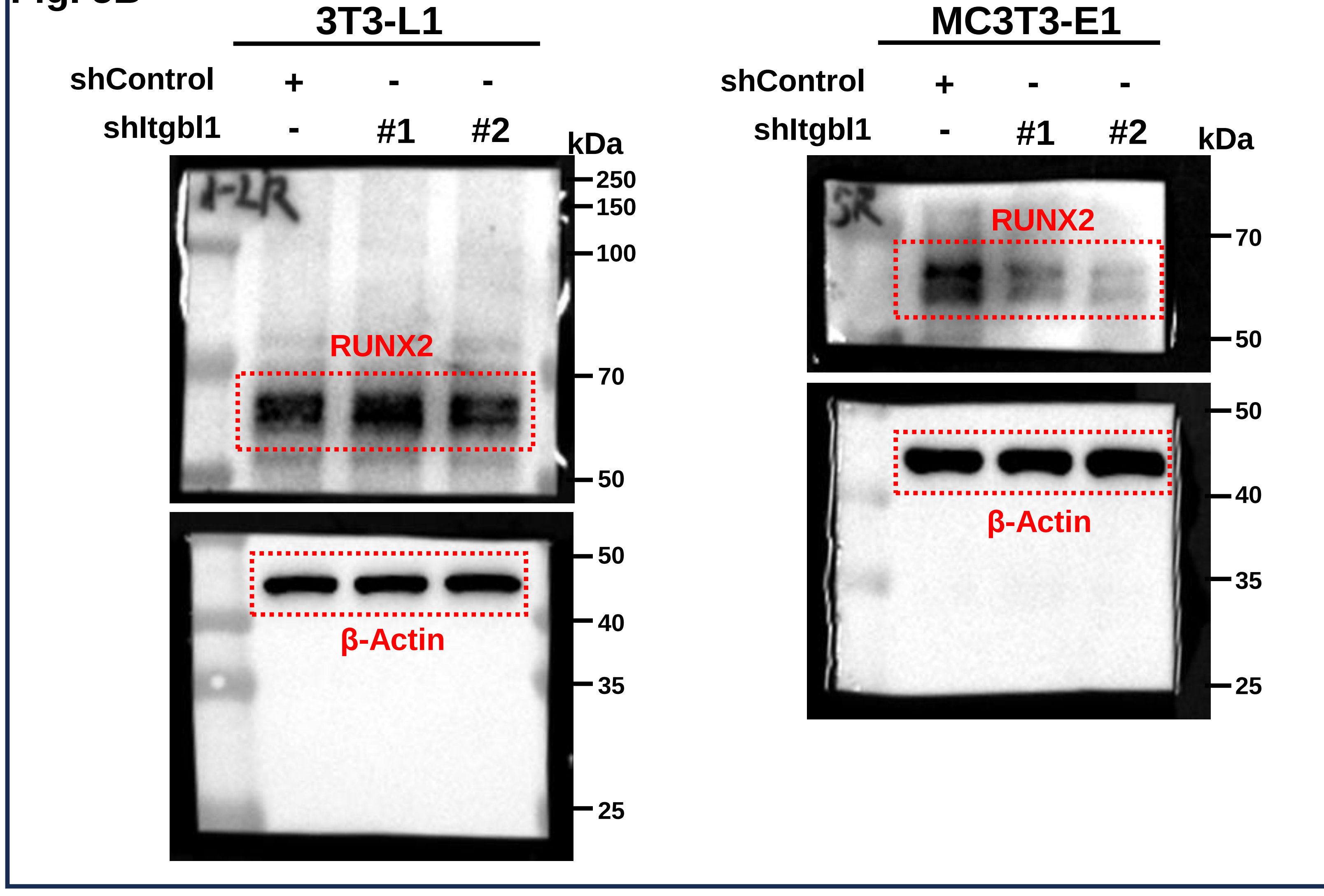


Fig. 5E

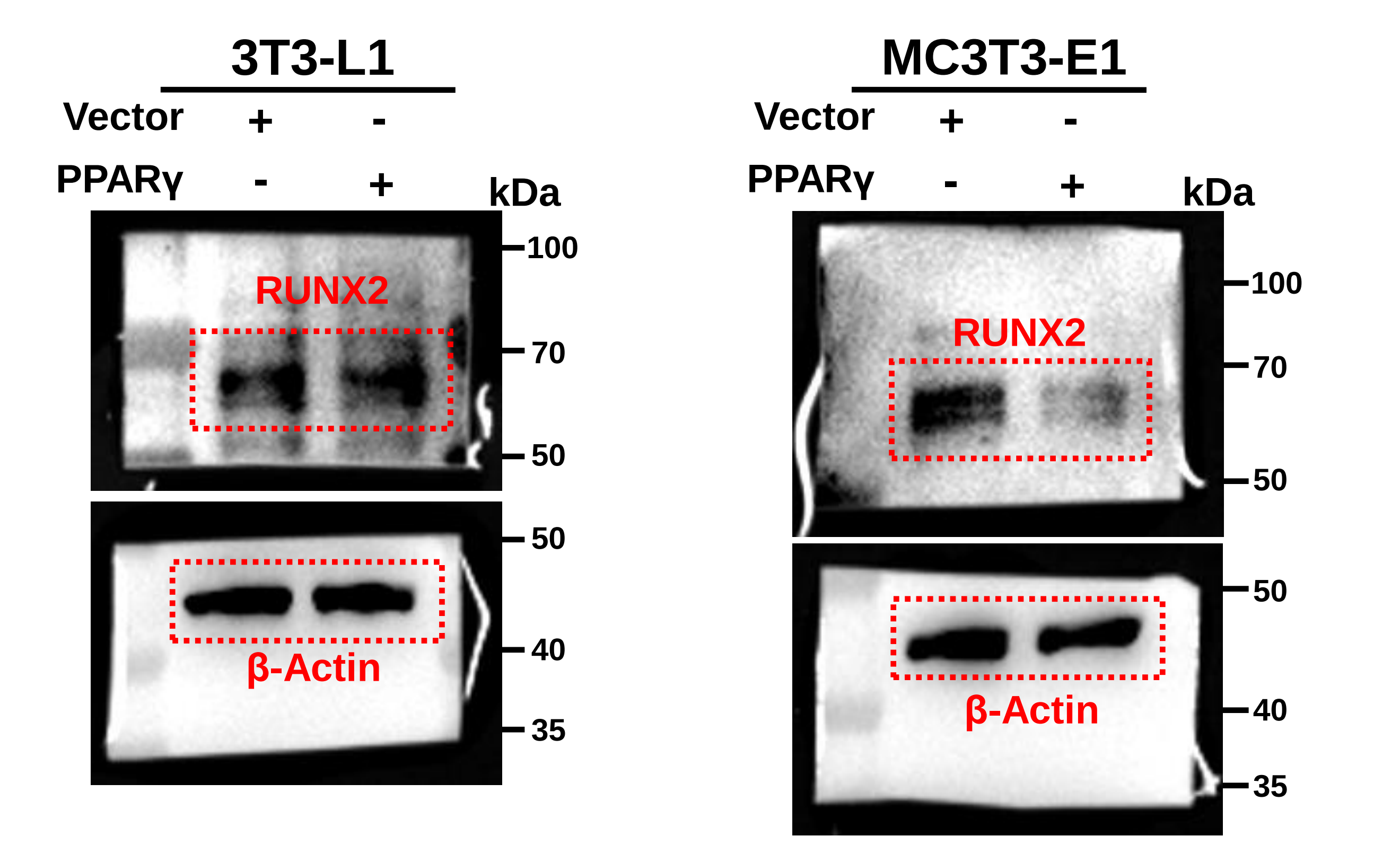


Fig. 5H

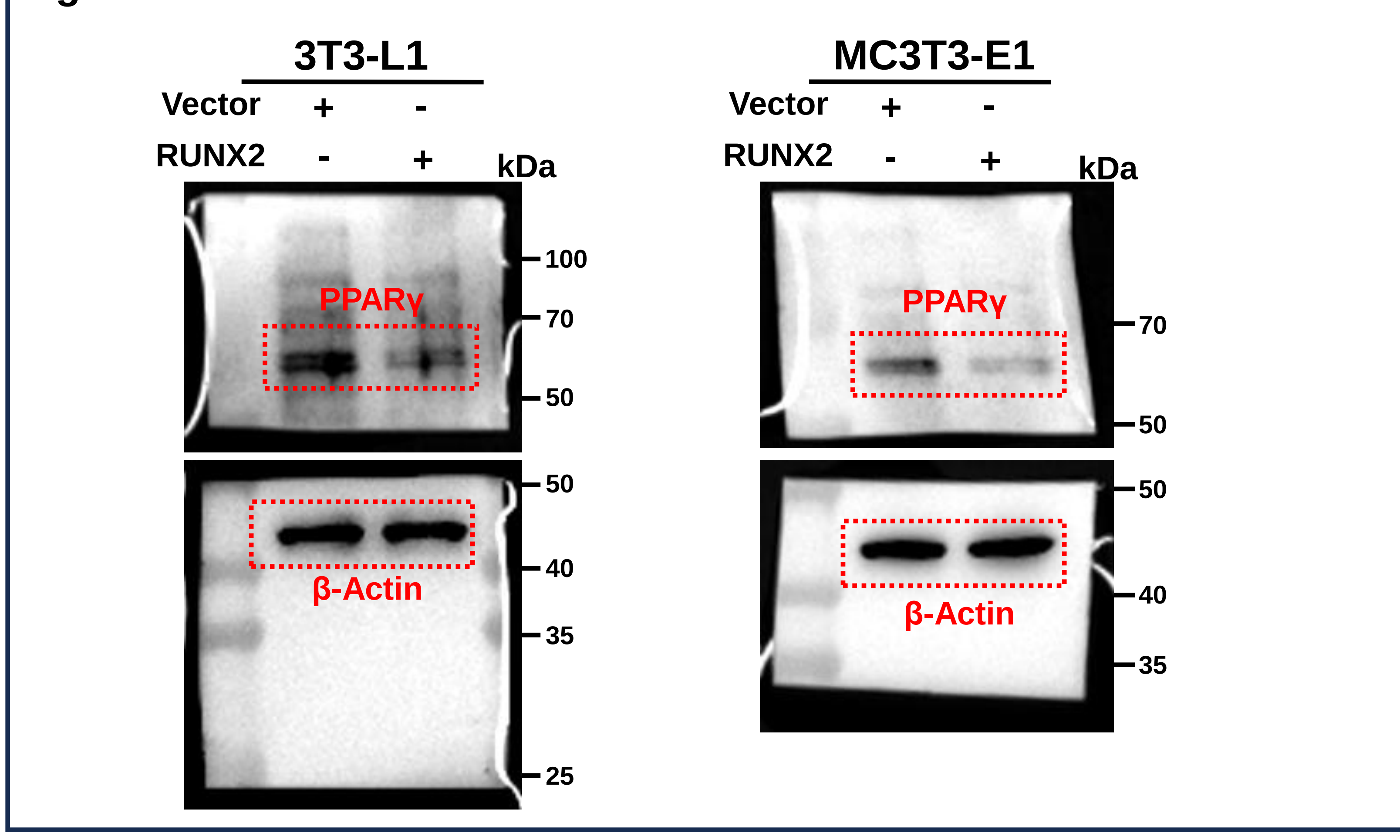

# Source data of Fig. S5

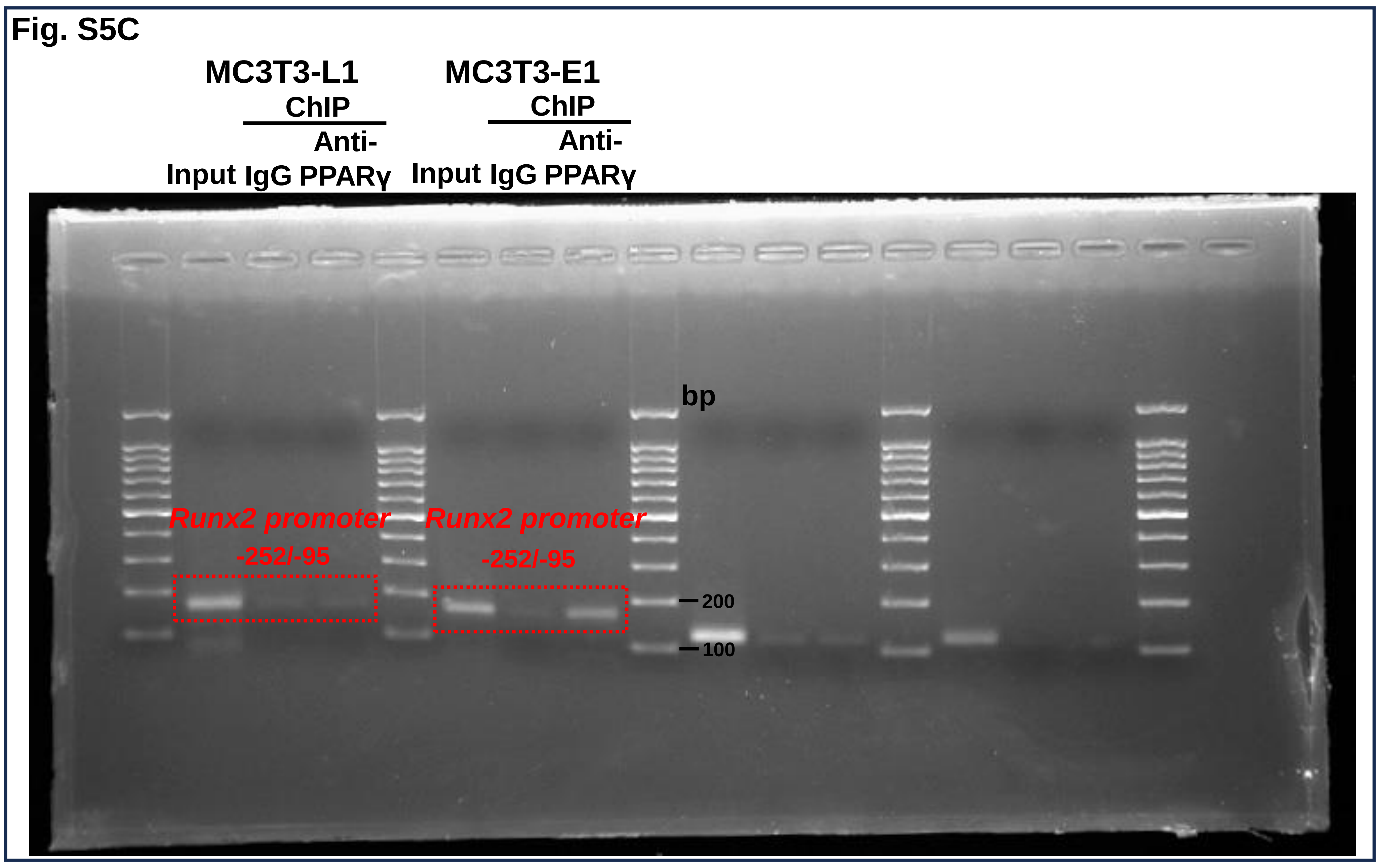


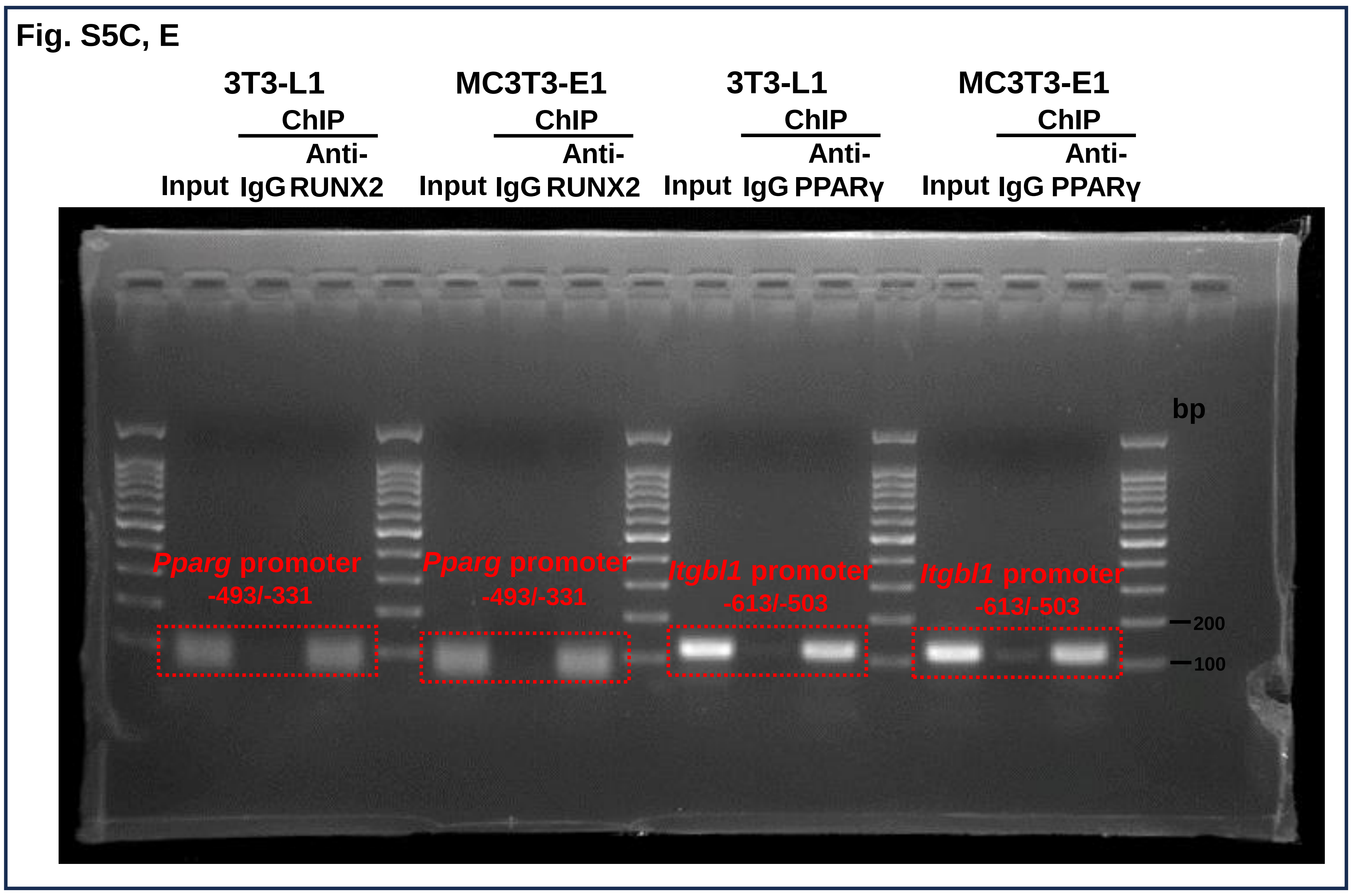


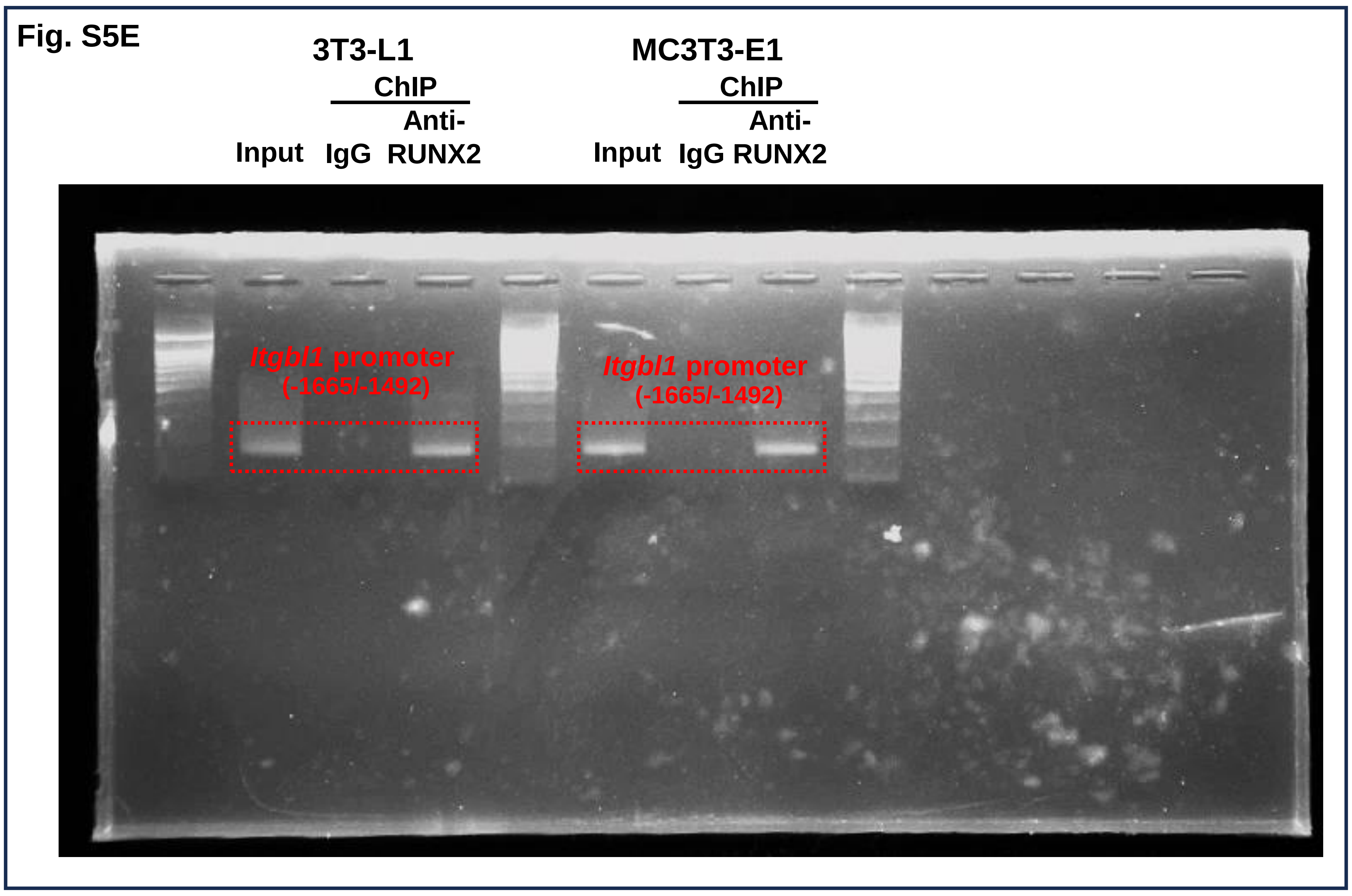


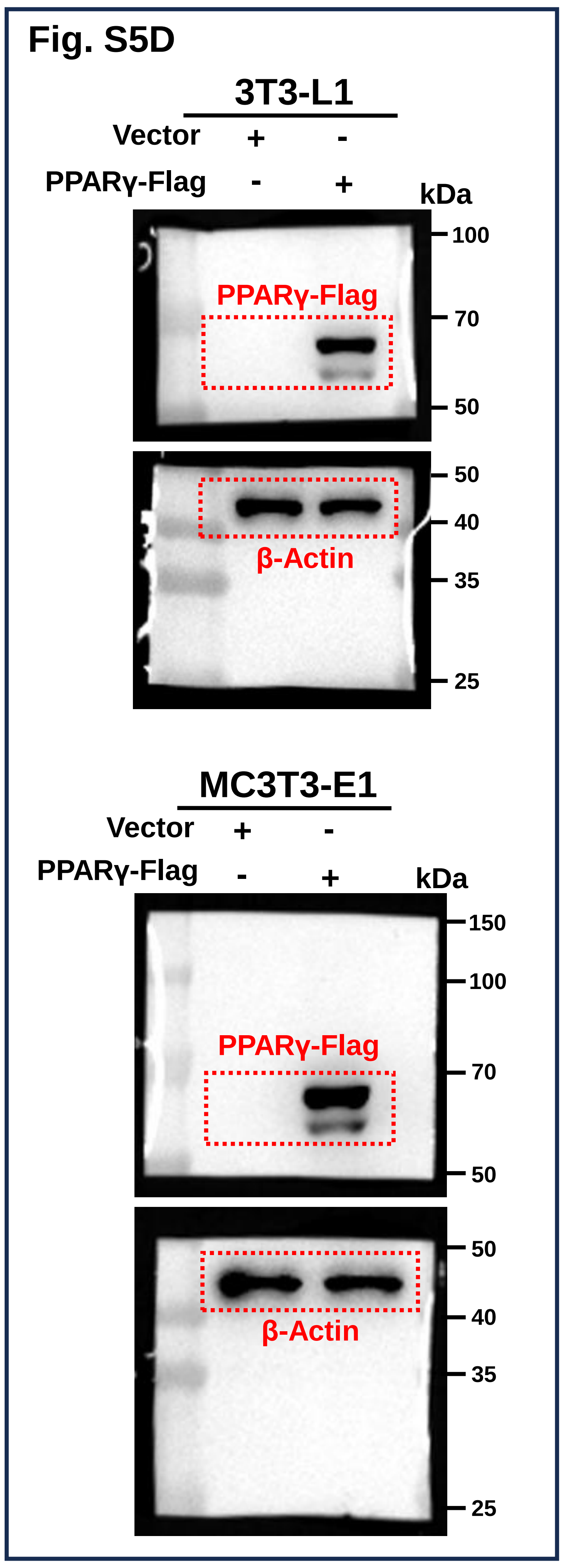


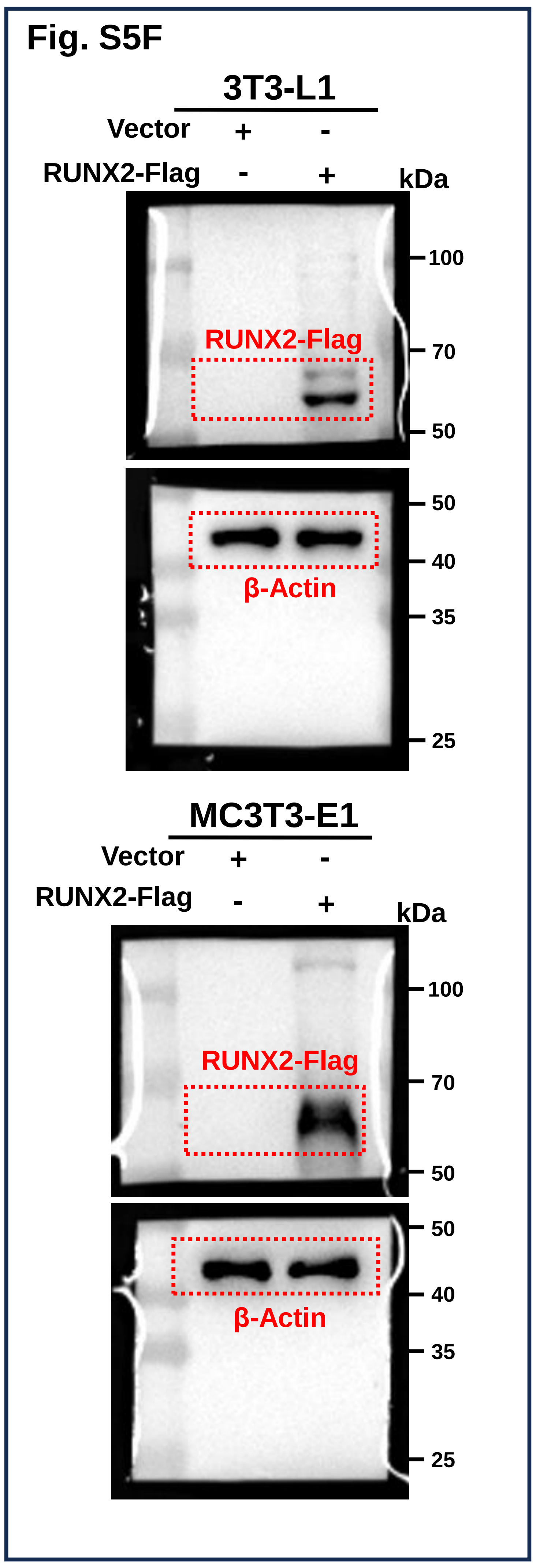

Supplement: Supplementary file 1 [file 41419_2026_9220_MOESM2_ESM.pdf]
