# Supplementary material for: ITGBL1 inhibits obesity and osteoporosis by maintaining adipogenic and osteogenic differentiation balance via the NEDD4–PPARγ–RUNX2 axis
Source: Cell Death Dis. 2026 Sep 2;17(1):926. doi: 10.1038/s41419-026-09220-z (PMC13722705; doi:10.1038/s41419-026-09220-z)
Supplement: Supplementary file 2 [file 41419_2026_9220_MOESM1_ESM.docx]

Supplementary Materials for

# ITGBL1 Inhibits Obesity and Osteoporosis by Maintaining Adipogenic and Osteogenic Differentiation Balance via the NEDD4–PPARγ–RUNX2 Axis

Hao Tan, Jia-Shuai Zhong, Lin-Sen Li, Rui Zhang, Wen-Xin Yan,

Tian-Hao Zhou, Qing-Shan Wang, Yu-Mei Feng*

*Corresponding author. Yu-Mei Feng, ymfeng@tmu.edu.cn

**This file includes:**

Supplementary Tables

Supplementary Figures and Figure Legends

**Supplementary Tables**

**Supplementary Table 1 Sequences of primers for genotyping, RT‒qPCR and ChIP**–**qPCR**

| **Genotype**  **or gene** | **Forward primer** | **Reverse primer** |
| --- | --- | --- |
| **Primers for genotyping of *Itgbl1*** | | |
| WT | ATTTAACTGTGGCTATGAGCCTGC | GTATGGTTACAGACTGGGTGATTG |
| *Itgbl1*^–/–^ | GATGGCCCTGTCACACCTTG | CTCCAGTGTCGATGTGGCTG |
| **Primers for RT‒qPCR assays** | | |
| *Bmp2* | CAAAGCAGGACCAGTGGGAA | AGCCCCCTGGAAGGGATTAT |
| *Cd36* | ATGGGCTGTGATCGGAACTG | GTCTTCCCAATAAGCATGTCTCC |
| *Cebpa* | CAAGAACAGCAACGAGTACCG | AGGCGGTCATTGTCACTGGT |
| *Fabp5* | ATGGCAACAACATCACGGTC | CTGCCATCAGCTGTCGTTTC |
| *Fabp7* | TGGGAAACGTGACCAAACCA | AGCTTGTCTCCATCCAACCG |
| *Gapdh* | ATTGTCAGCAATGCATCCTG | ATGGACTGTGGTCATGAGCC |
| *Itgbl1* | GAGTCCGAACGCAGATGTCG | CACTCACACAGTGGACCGAA |
| *Lpl* | AGGCATACAGGTGCAACTCC | TAGGGCATCTGAGAGCGAGT |
| *Nedd4* | GTACTCTCGGAGGACGAGGTAT | TTGTCTGCACGCTGGTAAGG |
| *Pparg* | GAGCACTTCACAAGAAATTACC | GAACTCCATAGTGGAAGCCT |
| *Runx2* | CTCTGCACCAAGTCCTTTTAATC | AGGAGGGGTAAGACTGGTCATAG |
| *Spp1* | AAGCATCCTTGCTTGGGTTTG | TGGTCGTAGTTAGTCCCTCAGAA |
| *Tgfb1* | GGCTCATTGGGCAGCTTTTG | AAGCGGAAGACCCTGAACTC |
| *Tgfb2* | TCTCTGTCCACTTGCACCAC | TGATAGGGGACGTGGGTCAT |
| *Tnfsf11* | CCTGTACTTTCGAGCGCAGA | GCATTGATGGTGAGGTGTGC |
| **Primers for ChIP**–**qPCR assays** | | |
| *Runx2* | ACAGAGGAACACCCATAA | AAAGTAAAGTGGGACTGC |
| *Pparg* | TTCTTCCAGAGGACCCTTGTTC | GCTGGAGTTACAGGTGGTTGGT |
| *Itgbl1* | CCTGGTCCTCTGGAATTGGT | TGCCAGATGGAAGAGGCATA |
|  | GGGAAGGAATGTAATGCTAACG | CTTCGGGCTTCTTCATCAAAC |

**Supplementary Table 2 Antibodies used in immunoreaction-based assays**

| **Name** | **Company** | **Catalogue number** | **RRID** | **Dilution** |
| --- | --- | --- | --- | --- |
| **Primary antibody** | | | | |
| α-Tubulin | Cell signaling technology | 2125 | AB_2619646 | IB 1:1000 |
| β-Actin | Abclonal | AC026 | AB_2768234 | IB 1:10000 |
| CD73 | Biolegend | 127205 | AB_1089065 | FC 1:200 |
| CD90.2 | Biolegend | 140303 | AB_10642686 | FC 1:200 |
| CD34 | Biolegend | 128607 | AB_1279222 | FC 1:100 |
| CD14 | Biolegend | 123315 | AB_10641133 | FC 1:100 |
| CD45 | Biolegend | 103116 | AB_312981 | FC 1:200 |
| Flag | Cell signaling technology | 14793 | AB_2572291 | IP 1:50  IB 1:1000  ChIP 1:50 |
| GFP | Abcam | ab183734 | AB_2732027 | IHC 1:200 |
| GAPDH | Abclonal | AC002 | AB_2736879 | IB 1:5000 |
| HA | Cell signaling technology | 3724 | AB_1549585 | IB 1:1000 |
| His | Cell signaling technology | 2365 | AB_2115720 | IB 1:1000 |
| HLA-DR | Biolegend | 107632 | AB_2650896 | FC 1:200 |
| ITGBL1 | ATLAS | HPA005676 | AB_1851948 | IHC 1:200 |
| Mouse-IgG | Abclonal | AC011 | AB_2770414 | IP 1:50 |
| NEDD4 | Cell signaling technology | 2740 | AB_2149312 | IB 1:1000 |
| PPARγ | Cell signaling technology | 2435 | AB_2166051 | IB 1:1000  IHC 1:400  ChIP 1:100 |
|  | Cell signaling technology | 2443 | AB_823598 | IP 1:50 |
| RUNX2 | Santa Cruz  Abcam | sc-390351  ab236639 | AB_2892645  AB_2937078 | IB 1:1000  ChIP 1:50 |
|  |  |  |  | IHC:1:2000 |
| Rabbit-IgG | Proteintech | 30000-0-AP | AB_2819035 | IP 1:50 |
| Ubiquitin | Cell signaling technology | 3933 | AB_2180538 | IB 1:1000 |
| **Secondary antibody** | | | | |
| Anti-Mouse | Abclonal | AS003 | AB_2769851 | IB 1:2000 |
| Anti-Rabbit | Abclonal | AS014 | AB_2769854 | IB 1:2000 |
| Anti-Rabbit (L) | Abclonal | AS061 | AB_2864055 | IB 1:5000 |

IB: immunoblot; IP: immunoprecipitation; ChIP: chromatin immunoprecipitation; IHC: immunohistochemistry; FC: Flow cytometry

**Supplementary Table 3 Sequences of siRNAs and shRNAs**

| **siRNA/shRNA** | **Sequence** |
| --- | --- |
| siItgbl1#1 | Sense: GGAUAUGCGAGACCUACGA(dT)(dT) |
|  | Antisense: UCGUAGGUCUCGCAUAUCC(dT)(dT) |
| siItgbl1#2 | Sense: GGUAGUUCUGAUCUUCCUU(dT)(dT) |
|  | Antisense: AAGGAAGAUCAGAACUACC(dT)(dT) |
| siNedd4 | Sense: GUCACAAAUCAAGAGUUAA(dT)(dT) |
|  | Antisense: UUAACUCUUGAUUUGUGAC(dT)(dT) |
| shItgbl1#1 | GTTCTGGAGAAGAGTGGTATA |
| shItgbl1#2 | CGGAAGTGTAATATGACAGAA |

**Supplementary Figures and Figure Legends**

**Supplementary Fig. 1 ITGBL1 deficiency enhances‌ adipogenic differentiation and inhibits osteogenic differentiation potential of MSCs and precursor cells. A** Representative morphological image of ADMSCs and BMSCs derived from WT and *Itgbl1^–/–^* mice. **B** Identification of MSCs by flow cytometry analysis of CD90^+^, CD73^+^, CD34^+^, CD45^+^, CD14^+^, and HLA-DR^+^ cell populations. **C** Morphological images of the indicated 3T3-L1 and MC3T3-E1 cells. **D** Efficiency of *Itgbl1* knockdown or overexpression in 3T3-L1 and MC3T3-E1 cells via lentivirus infection. **E** Expression of Bone Remodeling, Osteoclast Differentiation, and Osteoblast Differentiation gene sets in ADMSCs from WT and *Itgbl1*^–/–^ mice was compared by GSEA, and the corresponding representative genes were plotted as heat maps. The data are presented as the mean ± SD. Statistical analyses were performed with the unpaired Student’s t test. **P* < 0.05 compared with the control. Related to Fig. 2.

**Supplementary Fig. 2 ITGBL1 deficiency enhances‌ adipogenic differentiation and inhibits osteogenic differentiation potential of MSCs and precursor cells. A** and **B** Expression of adipogenesis-associated genes (*Pparg*, *Cebpa*, *Cd36*, *Fabp5*, *Fabp7*, and *Lpl*) in ADMSCs (**A**) and BMSCs (**B**) was measured by RT‒qPCR. **C** and **D** Expression of osteogenesis-related genes (*Bmp2*, *Runx2*, and *Spp1*; **C**) and bone remodeling-related genes (*Tnfsf11, Tgfb1*, and *Tgfb2*; **D**) in BMSCs was measured by RT‒qPCR. **E** and **F** Expression of adipogenesis-associated genes (*Pparg*, *Cebpa*, and *Cd36*) in 3T3-L1 cells (**E**) and osteogenesis-related genes (*Bmp2*, *Runx2*, and *Spp1*) in MC3T3-E1 cells (**F**) was measured by RT‒qPCR. The data are presented as the mean ± SD. Statistical analyses were performed with the unpaired Student’s t test. **P* < 0.05 compared with the control. Related to Fig. 2.

**Supplementary Fig. 3 ITGBL1 facilitates the ubiquitin-mediated degradation of PPARγ through interactions with PPARγ. A** HEK293T cells were transfected with plasmids encoding ITGBL1-Flag. The interaction between ITGBL1 and PPARγ was detected by Co-IP and IB with the indicated antibodies. **B** ADMSCs derived from C57BL/6J mice were transfected with plasmids encoding ITGBL1-Flag, after which nuclear and cytoplasmic fractions were prepared. The interaction between ITGBL1 and PPARγ in the nucleus or cytoplasm was detected by Co-IP and IB with the indicated antibodies. **C** and **D** HEK293T cells were co-transfected with Ub-HA, PPARγ-Flag, and/or ITGBL1-His plasmids, and treated with 10 μM MG132 for 6 h. Ubiquitination assays for exogenous (**C**) or endogenous (**D**) PPARγ protein in the indicated cells were performed by IP and IB with the indicated antibodies. Related to Fig. 3.

**Supplementary Fig. 4 Validation of siRNA-mediated NEDD4 knockdown efficiency in 3T3-L1 and MC3T3-E1 cells.** 3T3-L1 and MC3T3-E1 cells were transiently transfected with siNedd4 or siControl and collected at 48 or 72 h after transfection. The mRNA and protein levels of NEDD4 in the indicated cells were detected by RT–qPCR and IB. The data are presented as the mean ± SD. Statistical analyses were performed with the unpaired Student’s t test. **P* < 0.05 compared with the control. Related to Fig. 4.

**Supplementary Fig. 5 Mutual transcriptional regulation among ITGBL1, PPARγ and RUNX2 in cells of osteogenic lineage and adipogenic lineage. A** Schematic diagram of candidate PPARγ-binding sites in the proximal promoter region of *Runx2* or *Itgbl1* within 2 kb upstream of the TSS. **B** Schematic diagram of candidate RUNX2-binding sites in the proximal promoter region of *Pparg* or *Itgbl1* within 2 kb upstream of the TSS. **C** Enrichment of PPARγ on the *Runx2* or *Itgbl1* promoter in the indicated cells was assessed by ChIP–qPCR. **D** 3T3-L1 and MC3T3-E1 cells were infected with lentivirus carrying *Pparg* cDNA. The mRNA and protein levels of PPARγ in the indicated cells were detected by RT–qPCR and IB. **E** Enrichment of RUNX2 on the *Pparg* or *Itgbl1* promoter in the indicated cells was assessed by ChIP–qPCR. **F** 3T3-L1 and MC3T3-E1 cells were infected with lentivirus carrying *Runx2* cDNA. The mRNA and protein expression levels of RUNX2 in the indicated cells were detected by RT–qPCR and IB. The data are presented as the mean ± SD. Statistical analyses were performed with the unpaired Student’s t test. **P* < 0.05 compared with the control. Related to Fig. 5.

**Supplementary Fig. 6 Biodistribution and persistence of AAV9-mediated transgene expression after systemic administration.** WT male C57BL/6J mice were injected with AAV9-GFP (2 × 10^11^ vg) or PBS via tail vein at age of 4 weeks. Tissues were collected after 2 and 16 weeks of AAV9-GFP injection. Representative IHC staining images of GFP in the indicated organs of mice. Related to Fig. 7.

**Supplementary Fig. 7 ITGBL1 treatment alleviates HFD-induced obesity and osteoporosis.** WT male C57BL/6J mice fed a CD or HFD were injected with AAV9-Itgbl1 or AAV9-Vector (2 × 10^11^ vg) via tail vein at 4 weeks of age and allowed to grow to 20 weeks of age (n = 10 per group). **A** and **B** Representative IHC staining images of GFP, ITGBL1, PPARγ and RUNX2 in iWAT (**A**) and distal femur marrow tissue (**B**) of mice. GFP staining indicates the efficiency of AAV9 infection. Related to Fig. 7.

**Supplementary Fig. 8 ITGBL1 treatment alleviates osteoporosis in OVX mice.** WT female C57BL/6J mice fed a CD underwent OVX or sham surgery and were injected with AAV9-Itgbl1 or AAV9-Vector (5 × 10^10^ vg) via the knee joint cavity at 12 weeks of age, and then allowed to grow to 28 weeks of age (n = 10 per group). **A** Representative IHC staining images of GFP, ITGBL1, PPARγ and RUNX2 in the distal femur marrow tissue. GFP staining indicates the efficiency of AAV9 infection. **B** Representative images and quantification of uteri collected from the indicated mice. The data are presented as the mean ± SD. Statistical analyses were performed with the unpaired Student’s t test. **P* < 0.05 compared with the control mice. Related to Fig. 8.

**Supplementary Fig. 9 ITGBL1 treatment inhibits obesity and osteoporosis caused by ITGBL1 deficiency.** WT and *Itgbl1^–/–^* male C57BL/6J mice fed with CD were injected with AAV9-Itgbl1 or AAV9-Vector via tail vein (2 × 10^11^ vg) at 4 weeks of age and allowed to grow to 16 weeks of age (n = 10 per group). **A** and **B** Representative IHC staining images of GFP, ITGBL1, PPARγ and RUNX2 in iWAT (**A**) and distal femur marrow tissue (**B**). GFP staining indicates the efficiency of AAV9 infection. Related to Fig. 9.
